# Adaptive Cinchona Alkaloid-Derived Organoboron Crystalline Sponges Enable Enantioseparation of Weakly Interacting Molecules

**DOI:** 10.1021/jacs.6c09766

**Published:** 2026-10-02

**Authors:** Maximilian Rajski, Michał Terlecki, Iwona Justyniak, Arkadiusz Kornowicz, Michał K. Leszczyński, Mikołaj Cieślak, Janusz Lewiński

**Affiliations:** † Faculty of Chemistry, 49566 Warsaw University of Technology, Noakowskiego 3, 00-664 Warsaw, Poland; ‡ 119463 Institute of Physical Chemistry, Polish Academy of Sciences, Kasprzaka 44/52, 01-224 Warsaw, Poland

## Abstract

The enantioseparation of small, weakly interacting organic molecules remains a fundamental challenge, as they lack the functional groups required for efficient chiral recognition. Consequently, their enantiopure forms are typically accessed *via* asymmetric synthesis rather than resolution. Herein, we demonstrate that adaptive supramolecular crystalline sponges assembled from air- and moisture-stable cinchona alkaloid-derived organoboron complexes enable efficient enantiodiscrimination of small ethers, lactones, and cyclic ketones through guest inclusion within porous crystalline frameworks. Structural analysis reveals that enantioselectivity arises from adaptive confinement within homochiral cavities, where subtle framework rearrangements and modulation of cavity volume amplify cooperative weak host–guest interactions. Systematic tuning of the molecular building blocks affords crystalline sponges capable of resolving challenging substrates such as the biobased green solvent 2-methyltetrahydrofuran, γ-valerolactone, and methylcyclohexanone isomers with enantiomeric excesses of up to 76%. These findings establish adaptive confinement in supramolecular crystalline sponges as a general mechanism for chiral discrimination in the solid state and provide a conceptual framework for enantioseparation beyond conventional crystallization-based approaches.

## Graphical abstract

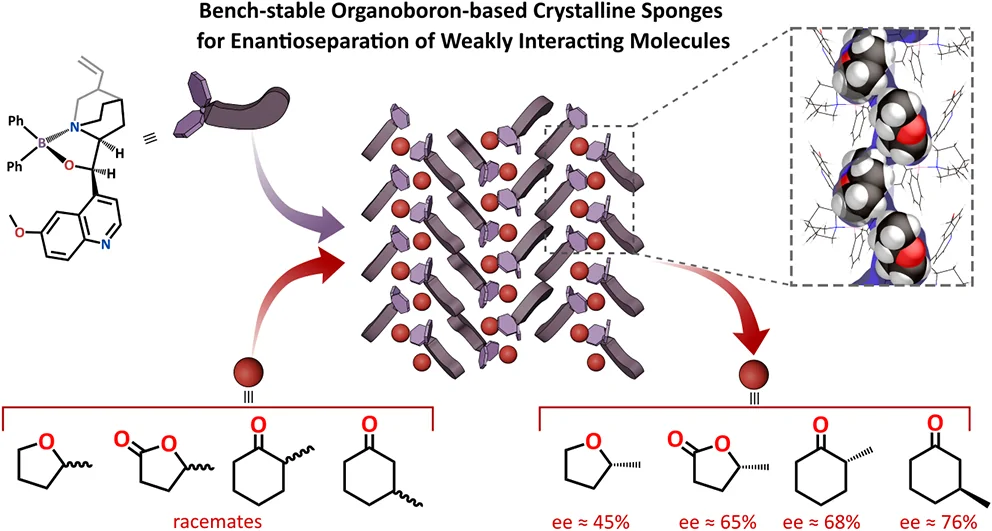

## Introduction

Enantioseparation remains a fundamental challenge in chemistry, as access to enantiopure compounds underpins advances in asymmetric catalysis, pharmaceuticals, and functional materials.^1–4^ While numerous strategies enable efficient resolution of molecules containing strong hydrogen-bond donors, charged functionalities, or aromatic groups, the resolution of small, weakly interacting organic molecules, such as ethers, lactones, and simple ketones, remains particularly demanding and largely unexplored.^5,6^ Crystallization-based resolution has long served as a cornerstone of enantioseparation and typically relies on the formation of diastereomeric multicomponent crystals, including salts or cocrystals with chiral resolving agents.^6,7^ In such systems, enantioselectivity usually arises from the formation of strong directional intermolecular interactions between coformers, most commonly hydrogen bonding or ionic contacts. Subtle variations in crystallization conditions, including solvent or solvate composition, can significantly influence chiral discrimination and may even induce chirality switching in the resulting crystalline phases.^8^

Despite their effectiveness, crystallization-based strategies generally require functional groups capable of forming strong intermolecular interactions and are, therefore, poorly suited for resolving small, weakly interacting molecules.^7^ This limitation is particularly evident for chiral ethers, lactones, and cyclic ketones,^8–10^ for which no general crystallization-based enantioseparation strategies have been established. Among these, 2-methyltetrahydrofuran (MeTHF) represents a particularly compelling target: as a biobased solvent derived from lignocellulosic biomass and increasingly adopted as a sustainable alternative to THF in pharmaceutical and fine chemical synthesis,^11^ its enantiopure forms would be of considerable practical value yet remain inaccessible by resolution. As a result, access to enantiopure forms of such molecules typically relies on asymmetric synthesis, most commonly organocatalytic approaches rather than separation of racemic mixtures.^12–14^ In contrast, crystalline porous materials provide a fundamentally different platform for the enantioselective uptake of organic molecules by accommodating them in an ordered manner within the voids of preorganized host frameworks.^15–17^ The encapsulation within such crystalline sponges not only enables structural determination of guest molecules but also allows the specific, often chiral, environments of the confined pores to facilitate selective molecular inclusion with high spatial precision. Prominent classes of chiral crystalline sponges include metal–organic frameworks (MOFs)^4,18,19^ and metal–organic cages (MOCs),^20,21^ which feature modular coordination-driven architectures with tunable pore environments and have demonstrated considerable success in enantioselective recognition and separation through precisely engineered host–guest interactions.^19,22,23^ More recently, supramolecular crystalline sponges assembled through noncovalent interactions have emerged as complementary alternatives, offering enhanced structural adaptability and framework flexibility that can be advantageous for discriminating subtle differences in the molecular size, shape, and chirality.^24,25^ However, crystalline sponges capable of resolving small, weakly interacting molecules remain rare. Such systems present a particular challenge, because these guests usually lack strong or highly specific recognition handles. Their selective uptake must therefore arise from the cooperative interplay of geometric confinement, dispersion, weak electrostatic interactions, and framework adaptation rather than from a single dominant binding interaction.

Building on our earlier demonstration that organoaluminum complexes of cinchonine (*CN*-H) can serve as versatile chiral building blocks for the construction of helical and nanotubular supramolecular architectures,^26–28^ we sought to develop related systems capable of the practical enantioseparation of small organic molecules. While the development of a crystalline sponge based on the prototypical [MeAl­(*CN*)_2_] building unit provided proof of concept for this approach, displaying preferential incorporation of (S)-MeTHF with an enantiomeric excess (*ee*) of approximately 7%, its sensitivity to air and moisture significantly limits practical applications.^26^ Herein, we report a new family of cinchona alkaloid-derived organoboron complexes that constitute air- and moisture-stable building blocks for adaptive, bench-stable crystalline sponges capable of the enantioselective uptake of small ethers, lactones, and cyclic ketones. The noncovalent character of the resulting crystalline frameworks affords high structural adaptability, enabling enhanced complementarity with chiral guest molecules and thereby promoting effective enantiodiscrimination through geometric confinement and cooperative weak interactions without the need for strong or highly specific binding motifs. The developed materials are chemically robust and readily processable, allowing for facile guest recovery and reuse of the host matrix. Together, these features position adaptive crystalline sponges as particularly promising platforms for the enantioseparation of small organic molecules that are otherwise challenging to resolve by using conventional crystallization-based approaches.

## Results and Discussion

### Tailoring the Structure of Cinchona Alkaloid-Based Molecular Building Blocks

In our previous work, we utilized organoaluminum derivatives of cinchona alkaloids as building blocks for homochiral supramolecular frameworks.^26–28^ In this context, we demonstrated that complex [MeAl­(*CN*)_2_] (*CN*-H = cinchonine) self-assembles *via* noncovalent interactions into a supramolecular right-handed double-helical architecture featuring homochiral 1D tubular channels of approximately 8 Å, in diameter, filled with solvent molecules (Figure 1e,d,g).^26^ This noncovalent porous material functions as a crystalline sponge for THF and 2-methyltetrahydrofurane (MeTHF) without disrupting the host framework. Furthermore, for the chiral solvent, the material exhibits preferential incorporation of (S)-MeTHF over the (R)-enantiomer, corresponding to an enantiomeric excess (*ee*) of approximately 7% (Figure 1j). Although this approach laid the foundation for the challenging enantioseparation of MeTHF, the resulting *ee* remained low, and the organoaluminum-based building blocks exhibit sensitivity to air and moisture, significantly limiting their practical applications. These limitations motivated us to develop alternative systems with improved stability and enhanced host–guest complementarity. To address these challenges, we turned our attention to organoboron derivatives of cinchona alkaloids as potential building blocks for supramolecular crystalline sponges. In contrast to their organoaluminum counterparts, the organoboron compounds exhibit good stability toward water and air, enabling the processing of the resulting materials under ambient conditions.^29^ Moreover, the lower atomic weight of boron relative to aluminum may favor higher loading in comparable crystalline sponge materials.

**Figure 1:**
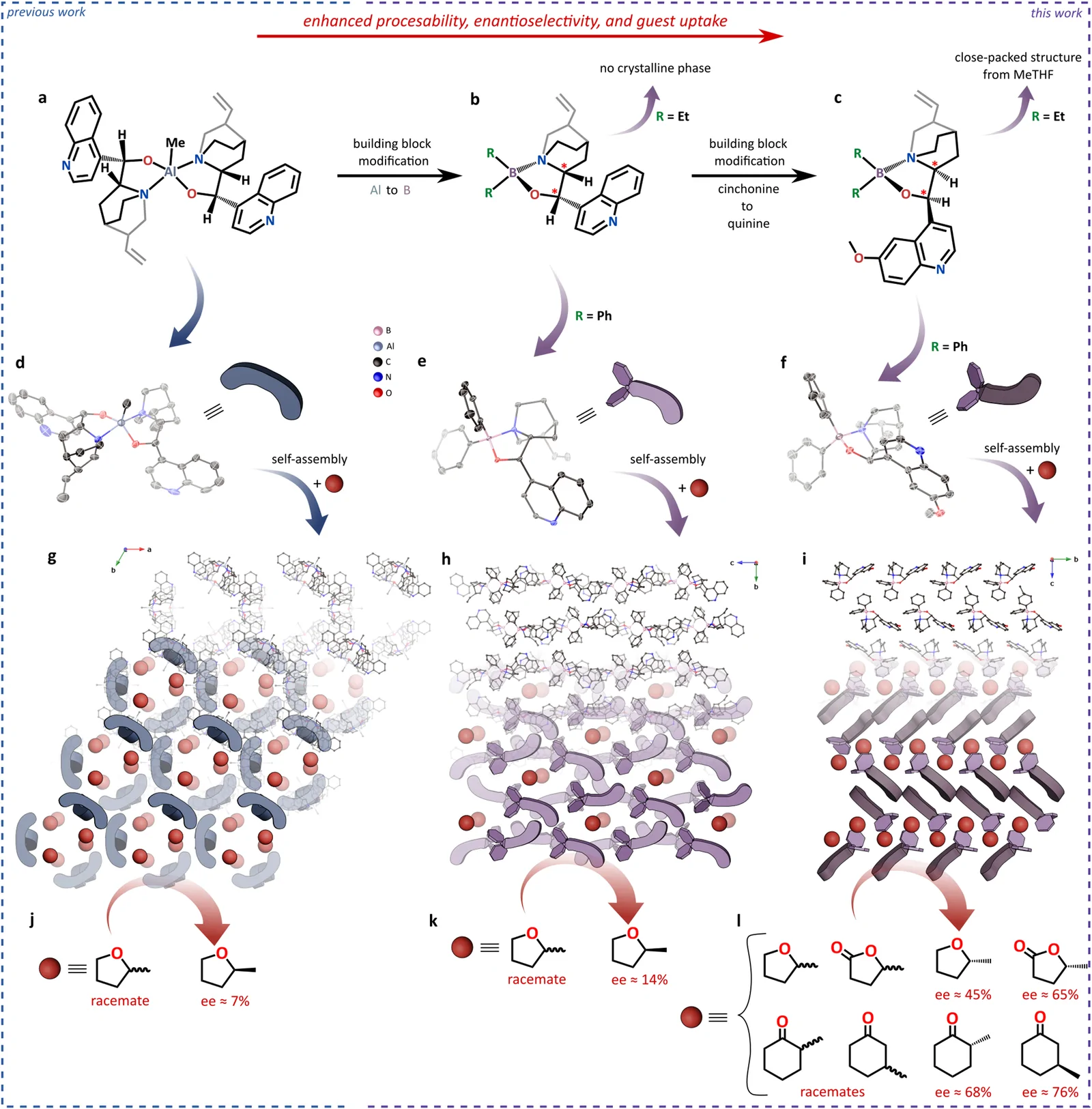
Successive structural tailoring of chiral cinchona alkaloid-based building blocks toward enhanced enantioseparation performance of crystalline sponges: (a–c) schematic representation of *CN*- and *QN*-stabilized organometallic complexes; (d–f) crystal structures of the molecular building blocks; (g–i) supramolecular architectures of the chiral crystalline sponges; (j–l) enantioseparation performance of the chiral crystalline sponges.

Accordingly, in reaction of *CN*-H with BEt_3_, we obtained a new complex [Et_2_B­(*CN*)] (**1**), representing a mononuclear aliphatic organoboron derivative of cinchonine. Although spectroscopic analysis confirmed the formation of **1** (*vide infra*), all attempts at crystallization yielded amorphous powders or slurries, preventing their use as a crystalline sponge. Consequently, we explored aromatic organoboron analogues containing phenylboron fragments. Incorporation of Ph_2_B units was expected to influence supramolecular assembly in two ways. First, phenyl substituents possess reduced conformational flexibility relative to aliphatic groups, which may promote more ordered packing arrangements. Second, aromatic rings can participate in weak intermolecular interactions such as CH−π and π–π contacts, potentially stabilizing supramolecular architectures.^30^ Furthermore, tetrahedral Ph_2_B fragments are structurally analogous to the “magic” Ph_2_C motif present in diarylhydroxymethyl groups known to enhance stereoselectivity in asymmetric transformations,^31–33^ suggesting that the specific steric and geometric properties of this unit may play a similarly beneficial role in chiral discrimination within the crystalline sponge framework. This strategy led us to the synthesis of the diphenylboron cinchonine complex [Ph_2_B­(*CN*)] (**2**), which readily forms crystalline materials exhibiting supramolecular channel structures (Figure 1b,e,h). Notably, this framework displays significantly improved enantioselectivity toward MeTHF inclusion compared to the previously reported aluminum system, affording approximately 14% *ee* of the (S)-enantiomer (Figure 1j) (*vide infra*).

The stereochemical features of cinchona alkaloids likely play a crucial role in the formation of chiral supramolecular assemblies. Thus, we next investigated the effect of replacing cinchonine with its pseudoenantiomeric analogue quinine (*QN*). Accordingly, diethyl- and diphenylboron derivatives of quinine were synthesized and examined (Figure 1c) (*vide infra*). While the diethylboron complex [Et_2_B­(*QN*)] (**3**) forms inclusion **3**·THF crystals in the presence of THF, its recrystallization from a racemic mixture of MeTHF produces a close-packed structure, lacking accessible cavities and therefore unsuitable for the role of an enantioselective crystalline sponge. In contrast, the aromatic analogue [Ph_2_B­(*QN*)] (**4**) forms a robust supramolecular framework that behaves as an efficient crystalline sponge for a wide range of chiral molecules, including MeTHF, γ-valerolactone (GVL), 2-methylcyclohexanone (2-MCH), and 3-methylcyclohexanone (3-MCH) (Figure 1f,i). Notably, the supramolecular framework of **4** provides significantly tighter encapsulation of guest molecules compared to [MeAl­(*CN*)_2_]- and [Ph_2_B­(*CN*)]-based sponges, leading to a marked enhancement in enantioselectivity, reaching an *ee* of 45% for MeTHF and 65–76% for other challenging chiral substrates (Figure 1l) (*vide infra*).

### Synthesis and Characterization of Organoboron Complexes

New mononuclear organoboron complexes [Et_2_B­(*CN*)] (**1**), [Ph_2_B­(*CN*)] (**2**), [Et_2_B­(*QN*)] (**3**), and [Ph_2_B­(*QN*)] (**4**) were synthesized *via* direct reactions of BEt_3_ or BPh_3_ with the respective cinchona alkaloid, *CN*-H or *QN*-H (Figure 2b). The synthesis of ethylboron derivatives was carried out in toluene at 110 °C, ensuring complete conversion. For complex **1**, all attempts at crystallization from various organic solvents (toluene, THF, MeTHF) resulted in amorphous white solids or slurries. In contrast, the *QN* analogue forms high-quality crystals of **3**·THF with high yield (89%) upon recrystallization from a THF solution at −20 °C. Reactions with BPh_3_ proceeded efficiently at 60 °C in THF, and postsynthetic crystallization of the parent solution at −20 °C afforded well-defined crystals of **2**·0.5THF and **4**·THF in high yields (92% and 88%, respectively). All resulting complexes were characterized by ^1^H and ^11^B NMR and Fourier transform infrared (FTIR) spectroscopy. The crystal structures of **2**·0.5THF, **3**•THF, and **4**·THF were determined by single-crystal X-ray diffraction (SC-XRD).

**Figure 2:**
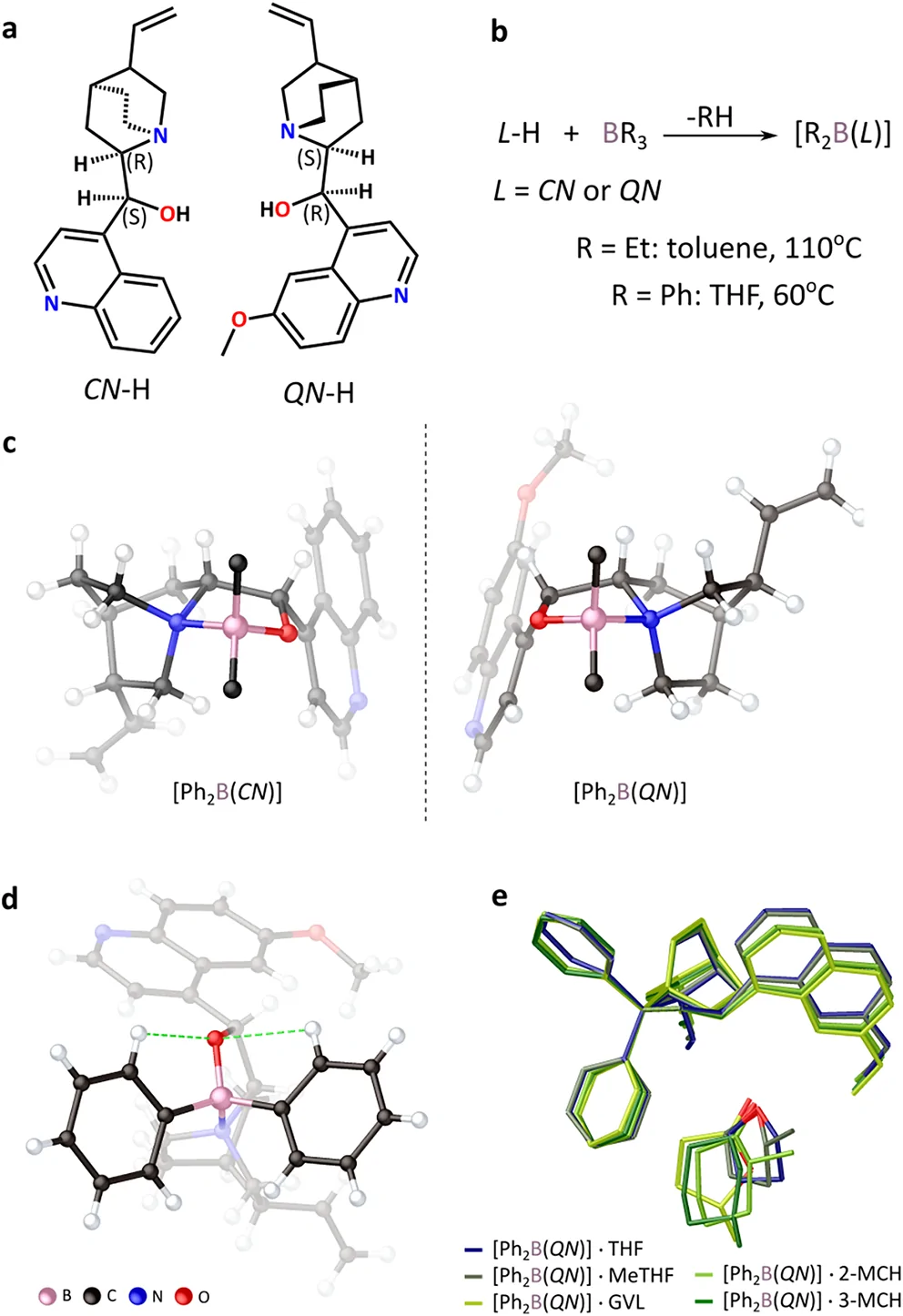
(a) Schematic representation of *CN*–H and *QN*–H proligands; (b) synthesis of ethyl- and phenylboron derivatives of cinchona alkaloids; (c) representation of the pseudoenantiomeric molecular structures of compounds **2** and **4** (phenyl rings omitted for clarity); (d) stabilization of the phenyl ring conformation *via* intramolecular C–H···O interactions in the molecular structure of **4**; (e) conformational variations of **4** in different solvates.

Spectroscopic analysis confirms that complexes **1–4** contain a single BR_2_ unit chelated by a deprotonated O,N-donor cinchona alkaloid ligand (Figure 1b,c). The ^1^H NMR spectra exhibit signals consistent with the corresponding cinchona backbone and B-bound ethyl or phenyl substituents (Figures S12–S15). The ^11^B NMR spectra display single resonances at 9.03–9.34 ppm and 6.43–6.84 ppm for Et- and Ph-derivatives, respectively, indicating the presence of a four-coordinate boron environment (Figure S16).^34^ SC-XRD analysis of complexes **2, 3** and **4** confirms the formation of a five-membered {BOCCN} chelate ring that adopts an envelope conformation typical of organoboron complexes with chelating ligands.^35,36^ Cinchonine and quinine represent pseudoenantiomers that differ in the configuration of the alcohol backbone, but share the same configuration at the quinuclidine moiety stereocenters (Figure 2a). This pseudoenantiomeric relationship is preserved in their diphenylboron derivatives, resulting in molecular structures that are approximate mirror images of one another, featuring inverted envelope conformations of the {BOCCN} rings (Figure 2c). The aromatic rings of the Ph_2_B moieties in **2** and **4** adopt similar cone-like conformations, likely stabilized by weak intramolecular C–H···O interactions involving the alkoxide oxygen atom of the O,N-donor ligand (Figure 2d). These interactions likely restrict conformational movements within the organoboron molecules, and, consequently, their molecular structures remain essentially unchanged in other solvated crystal forms (Figure 2e, *vide infra*).

Organoboron complexes **2–4** crystallize from THF solution as solvates exhibiting markedly different crystal packing arrangements. Complex **2** forms inclusion crystals with **2**·0.5THF stoichiometry in the orthorhombic, noncentrosymmetric space group *P*2_1_2_1_2_1_. In the crystal structure, molecules of organoboron complex **2** are arranged into 2D supramolecular grids that stack along the crystallographic *a* direction, forming tight, gated chiral channels filled with guest molecules (Figure 1h). These channels constitute approximately 12.1% of the unit cell volume, corresponding to ca. 159.7 Å^3^ per THF molecule (Table S9). Complex **3** crystallizes from THF solution as a **3**·THF solvate in the triclinic space group *P*1. The molecules of **3** form an extended 3D network with guest molecules encapsulated in closed, isolated cavities (Figure S4). The void volume constitutes approximately 20.3% of the unit cell volume, corresponding to ca. 130.2 Å^3^ per THF molecule (Table S9). Complex **4** crystallizes in the same *P*2_1_2_1_2_1_ space group as **2**·0.5THF, but differs substantially in both supramolecular arrangement and solvate stoichiometry. In the crystal structure of **4**·THF, the molecules adopt a “tail-to-tail” arrangement, forming supramolecular 2D bilayers parallel to the *ab* plane (Figure 1i). The guest molecules are tightly encapsulated within narrowly interconnected cavities formed between these bilayers. The void volume constitutes approximately 14.4% of the unit cell volume, corresponding to only ca. 106.1 Å^3^ per THF molecule.

### Separation of Racemic Mixtures of Organic Solvents *via* Inclusion in Crystal Matrices

The homochiral nature of the confinement spaces formed in the crystal lattices of organoboron complexes **2–4** offers potential for enantioselective recognition of encapsulated small chiral guests. With this in mind, we performed recrystallization experiments from a racemic mixture of MeTHF as a model chiral guest structurally close to THF. Notably, due to the absence of strongly interacting functional groups, the chiral resolution of this promising green solvent remains highly challenging.^11^ All attempts to obtain crystalline MeTHF solvates with complex **3** resulted in a microcrystalline powder, likely corresponding to an unsolvated form of this complex. PXRD analysis of the product revealed a new crystalline phase, distinct from that of **3**·THF (Figure S21), while Gas chromatographic (GC) and TGA experiments indicated no significant inclusion of MeTHF (Figures S18b and S23). In contrast, the phenylboron derivatives **2** and **4** readily form inclusion crystals with MeTHF while preserving the porous host framework of the corresponding THF solvates, thereby acting as robust crystalline sponges. The new solvates, **2**·0.5MeTHF and **4**·MeTHF, were characterized by SC-XRD, PXRD, and TGA.

TGA profiles of **2**·0.5MeTHF and **4**·MeTHF show sharp stepwise mass losses around 167 and 101 °C, respectively, indicating relatively tight encapsulation of MeTHF molecules and their thermally induced release (Figure S17b and S19b). Notably, the guest uptake is approximately 2-fold higher in the QN-derived system than in the CN analogue (14.9% vs 8.6%, Table S11), which is consistent with the stoichiometry of the corresponding solvated crystals. The encapsulated chiral guest molecules can be recovered from the crystal matrices by gentle heating at 120–160 °C under reduced pressure and collected in a liquid nitrogen trap. GC analysis of the released MeTHF revealed enantioselective uptake within the crystalline sponges, with *ee* values of approximately 14% and 45% for **2**·0.5MeTHF and **4**·MeTHF, respectively, representing a significant improvement over the previously reported [MeAl­(*CN*)_2_]-based system (*vide supra*) (Figure 3a-c). Interestingly, the pseudoenantiomeric relationship between complexes **2** and **4** translates into opposite enantioselectivities: **2** preferentially encapsulates (S)-MeTHF, whereas **4** favors the (R)-enantiomer. Thermal desorption of the guest molecules induces a rearrangement of the host supramolecular framework, accompanied by fragmentation of the single crystals or sample amorphization, as evidenced by PXRD analysis (Figures S20 and S22). The resulting unsolvated forms of the phenylboron complexes can be easily reused in subsequent crystallization processes (Figure 6a).

**Figure 3:**
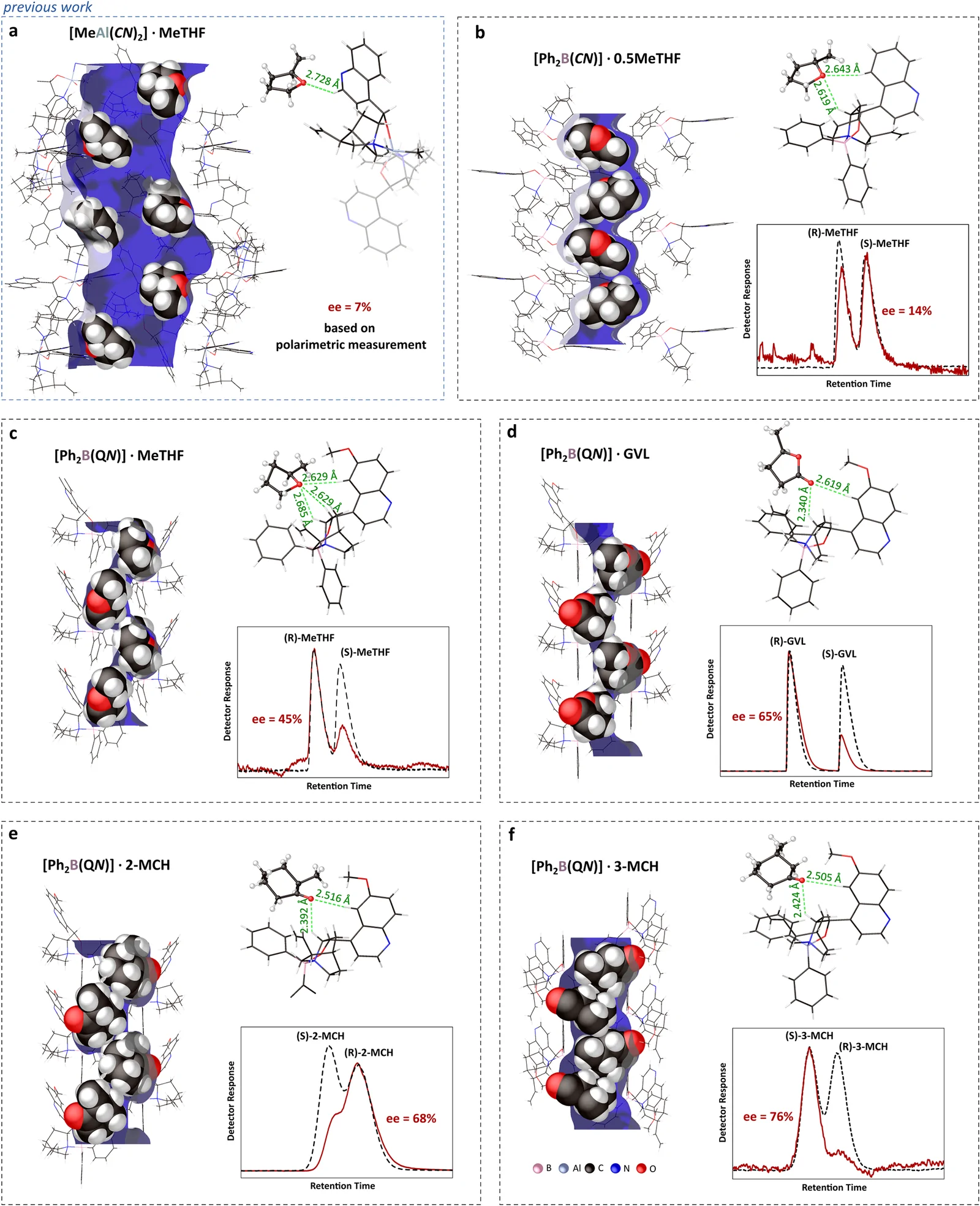
Representation of guest occupied voids [calculated as solvent-accessible voids (*r* = 1.4 Å); blue and gray colors denote the interior and exterior void surfaces, respectively], short intermolecular O···H contacts between guest and host molecules, and GC chromatograms of the released guest [red—released guest; black dashed—parent racemate] for [MeAl­(*CN*)_2_]·MeTHF (a), **2**·0.5MeTHF (b), **4**·MeTHF (c), **4**·GVL (d), **4**·2-MCH (e), and **4**·3-MCH (f).

The enhanced enantioselectivity observed along the series of crystalline sponges [MeAl­(*CN*)_2_] < **2** < **4** is likely associated with tighter encapsulation of the guest molecules within homochiral crystalline matrices with improved host–guest complementarity. In the crystal lattices of both organoboron complexes, the chiral guest molecules are confined within significantly smaller cavities than in the [MeAl­(*CN*)_2_]-based system (Figure 3a-c). The corresponding void volumes per guest molecule are 170 and 127 Å^3^ (Table S9) for **2**·0.5MeTHF and **4**·MeTHF, respectively, gradually approaching the van der Waals volume of a single MeTHF molecule (91 Å^3^, calculated according to^37^). In contrast, the analogous value for [MeAl­(*CN*)_2_]·MeTHF is as high as 427 Å^3^. This trend is further reflected in the lattice deformations induced upon inclusion of the more sterically demanding MeTHF compared to THF.

The MeTHF solvate of **4** exhibits an approximately 3.2% increase in unit cell volume relative to its THF analogue, whereas the corresponding expansion for **2** is limited to only 0.6% (Table S9). In contrast, the unit cell volume of [MeAl­(*CN*)_2_]·MeTHF is approximately 1% smaller than that of [MeAl­(*CN*)_2_]·THF, reflecting the relatively loose confinement of the guest molecules within the pores. The above observations demonstrate the substantial adaptability of the noncovalent frameworks formed by the chiral cinchona alkaloid complexes, with the supramolecular structure of **4** likely exhibiting the best geometric complementarity with MeTHF. Furthermore, all structures feature short intermolecular CH···O contacts involving the ether oxygen atoms of THF or MeTHF and the quinoline and/or α-amine protons of the cinchona alkaloid, which likely govern the orientation of guest molecules within the crystal lattices (Figure 3a–c). The combination of these directional interactions and the homochiral shape of the cavities is most likely responsible for the observed enantioselectivity in guest inclusion.

Encouraged by the promising results obtained for **4**·MeTHF, we extended our investigation to other structurally related small chiral organic molecules known to be challenging targets for enantioseparation, including γ-valerolactone (GVL), 2-methylcyclohexanone (2-MCH), and 3-methylcyclohexanone (3-MCH), for which conventional crystallization-based approaches are largely ineffective or unavailable.^8–10^ Recrystallization of **4** from racemic mixtures of these compounds afforded crystals of **4**·GVL, **4**·(2-MCH), and **4**·(3-MCH) in high yields, which were characterized by SC-XRD. Structural analysis revealed that, while **4**·GVL and **4**·(2-MCH) adopt crystal structures analogous to those of **4**·THF and **4**·MeTHF, the 3-MCH solvate forms a related supramolecular framework with lower *P*2_1_ symmetry and an approximately 2-fold smaller unit cell (see SI). Consequently, the supramolecular architecture of **4**·(2-MCH) differs slightly in the stacking arrangement of 2D bilayers formed by “tail-to-tail” arranged molecules of **4** (Figure S10), suggesting that while the formation of these bilayer motifs is primarily governed by intermolecular interactions between organoboron molecules, their relative stacking is modulated by the encapsulated guest.

The high flexibility of the crystal framework of **4** is reflected in changes in unit cell volumes across different solvates. The GVL solvate exhibits an approximately 3.4% larger unit cell than **4**·THF, and this difference increases to ca. 10% in the case of the solvates including larger 2-MCH and 3-MCH guests (Table S9). Notably, the relative expansion of the unit cell occurs mainly along the *c* direction, i.e., perpendicular to the 2D bilayers formed by molecules of **4**, reflecting the adaptive inclusion of guest molecules between the supramolecular bilayers, which likely supports geometrical discrimination of enantiomers. Indeed, GC analysis of the released guests revealed that the crystalline sponge based on complex **4** preferentially uptakes the (R)-GVL, (R)-2-MCH, and (S)-3-MCH enantiomers, with *ee* values of 65%, 68%, and 76%, respectively, acting analogously to the case of MeTHF (Figure 3d–f). These results highlight the importance of proper selection of a complementary host–guest system for effective enantioseparation.

Notably, the present system addresses a class of enantioseparation targets that remain rarely explored in chiral porous materials: small, weakly interacting guests. Unlike substrates equipped with strong or highly specific recognition handles, these molecules are discriminated through subtle differences in geometric fit and the cooperative interplay of weak intermolecular interactions. A numerical comparison of ee values across systems therefore compares different recognition regimes rather than different degrees of performance, and the values reached here—up to 76%—show that adaptive confinement alone can discriminate guests for which strong directional binding is unavailable. represent substantial discrimination within a substrate class for which strong directional binding is unavailable.

### Mechanistic Considerations

The crystallographic series itself provides the first indication that enantiodiscrimination is coupled to guest-responsive framework adaptation. Relative to **4**·THF, inclusion of MeTHF and GVL expands the unit cell by approximately 3.2 and 3.4%, respectively, whereas the larger 2-MCH and 3-MCH guests induce expansions approaching 10%, predominantly along the direction perpendicular to the supramolecular bilayers (Table S10). These changes demonstrate substantial structural plasticity of the noncovalent framework, but they do not by themselves explain why one enantiomer is preferentially incorporated. We therefore complemented the crystallographic analysis with quantitative host–guest interaction and docking calculations to determine how the guest-adapted cavity differentiates between enantiomeric orientations.

For the representative **4**/MeTHF system, host–guest interaction energies were evaluated with CrystalExplorer 21.5 at the B3LYP/6–31G­(d,p) level of theory (for details, see SI).^38,39^ To compare the two enantiomeric arrangements within the experimentally adapted host environment, optimized (R)- and (S)-MeTHF molecules were fitted to the electron-density maxima of the **4**·MeTHF crystal structure, affording models A^R^ and A^S^, respectively. Hirshfeld Surface (HS) and pairwise interaction energy analyses show that guest binding is dominated by van der Waals contacts, with dispersion providing the largest attractive contribution (Table 1) and H···H/H···C contacts (87–93% of the HS) accounting for most intermolecular contacts. Weak O···H contacts involving the ether oxygen provide an additional orientational component (Figures 3c and 4a,b). The electrostatic potential (ESP) maps on HS of **4** show two chemically distinct recognition regions: a predominantly negative ESP region between the phenyl rings of the Ph_2_B group and a strongly positive ESP region associated with the QN backbone (Figure 5b). Consistently, the experimentally observed guest orientation places the negative ESP around the ether oxygen atom toward a positively polarized region of the latter QN-derived host cavity (Figure 4a,b). Thus, recognition does not arise from a single strong interaction, but from the cooperative action of dispersion, weak directional electrostatics, and steric confinement within the chiral cavity. The energetic decomposition further shows that replacing (R)-MeTHF by the (S)-enantiomer in the same experimentally derived binding geometry (model A) increases the repulsive contribution (152.8 vs 196.8 kJ·mol^–1^) while leaving the attractive terms comparatively similar (Table 1). Consequently, the total interaction energy of A^R^ is more favorable than that of A^S^ by approximately 12.4 kJ·mol^–1^. This difference is therefore traced primarily to superior steric and shape complementarity of the preferred (R)-enantiomer within the adapted cavity, while electrostatic interactions help define the guest orientation. Molecular docking was then used to determine whether the less-favored enantiomer could avoid this steric penalty by adopting alternative poses. Simulations employing the CCDC GOLD software^40^ revealed that, for (R)-MeTHF, the predicted poses clustered around the experimentally observed arrangement represented by model A^R^. In contrast, docking of (S)-MeTHF resulted in a broader distribution of guest orientations (Figure S28). The highest-ranked pose for (S)-MeTHF (model B^S^) preserves a similar overall shape and methyl-group position as (R)-enantiomer but reverses the orientation of the ether oxygen atom (Figure 4c). This orientation decreases steric repulsion, but at the cost of disrupting electrostatic complementarity and the favorable O···H contacts; its total host–guest interaction energy therefore remains less favorable than for the preferred (R)-guest (Table 1). The calculations thus reveal an adaptive-fit recognition process in which the flexible crystalline environment accommodates the guest, but enantiodiscrimination emerges only when steric fit and electrostatic orientation can be satisfied simultaneously.

**Figure 4:**
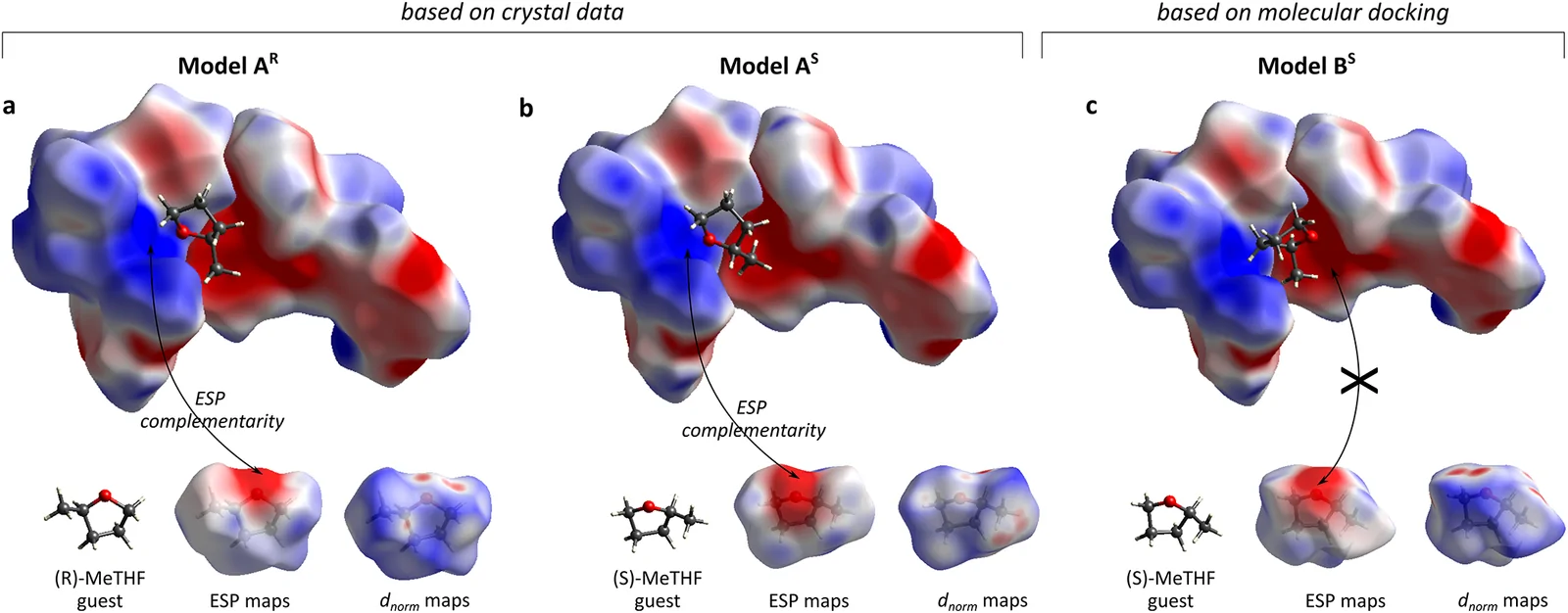
Steric and electrostatic complementarity in the **4**/MeTHF host–guest system. (a, b) Models A^R^ and A^S^ derived from the experimental **4**·MeTHF structure; (c) highest-ranked docking pose B^S^ for (S)-MeTHF. Electrostatic potential (ESP) [mapped from −0.05 au (red) to + 0.05 au (blue)] and *d*_norm_ maps on Hirshfeld surfaces illustrate the balance between electrostatic complementarity and steric fit.

**Figure 5:**
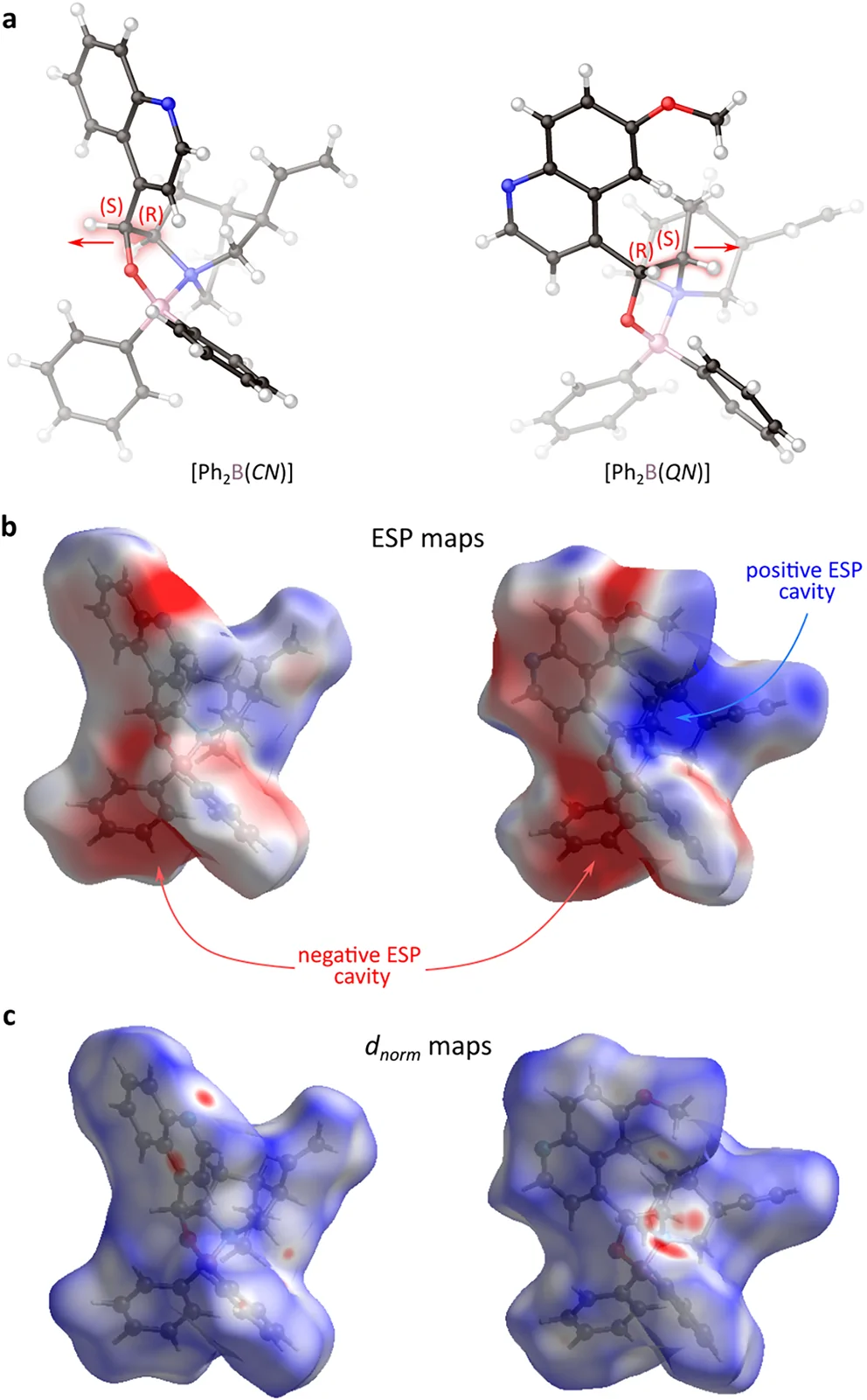
Comparison of the molecular recognition environments of the CN- and QN-derived diphenylboron hosts **2** and **4**. (a) Molecular structures highlighting the different orientations of the polarized C–H bonds at the stereogenic alkoxy backbone; (b) electrostatic potential (ESP) maps on Hirshfeld surface showing the more localized positive cavity in complex **4**; (c) corresponding *d*_norm_ maps on Hirshfeld surface highlighting differences in the guest recognition environment.

**Table 1:** Calculated Host–Guest Interaction Energies (*E*_tot_) and Their Electrostatic (*E*_ele_), Polarization (*E*_pol_), Dispersion (*E*_dis_), and Exchange-Repulsion (*E*_rep_) Components for Models A^R^, A^S^, and B^S^. Energies are given in kJ mol^–1^

Model A^R^					Model A^S^					Model B^S^				
*E*_ele_	*E*_pol_	*E*_dis_	*E*_rep_	***E***_**tot**_	*E*_ele_	*E*_pol_	*E*_dis_	*E*_rep_	***E***_**tot**_	*E*_ele_	*E*_pol_	*E*_dis_	*E*_rep_	***E***_**tot**_
–52.4	–11.8	–150.9	152.8	**–101.3**	–61.5	–12.0	–156.8	196.8	**–88.9**	–42.3	–10.1	–166.9	189.5	**–78.7**
Total binding:		–215.1			Total binding:		–230.3			Total binding:		–219.3		

To explore the respective effects of host architecture and guest structure on the enentiodiscrimination, similar calculations were also performed for the **2**·0.5MeTHF and **4**·(3-MCH) systems. Comparison of the CN-derived host **2** with the QN-derived host **4** shows that inversion of the α- and β-carbon configurations of the alkoxy backbone changes the orientation of the polarized C–H bonds at these stereogenic centers (Figure 5). In **4**, these C–H vectors point toward the same side of the molecule as the quinoline and ethylene fragments, generating a compact hollow region with a pronounced positive ESP. In **2**, the corresponding C–H bonds are directed oppositely, producing a more diffuse positive potential and a more diffuse, less well-defined recognition environment. This molecular difference is amplified by crystal packing: the larger voids in the framework of **2** provide looser confinement and therefore weaker steric exclusion. Accordingly, the calculated interaction energies for (R)- and (S)-MeTHF in **2** differ by only 2.6 kJ mol^–1^ (Tables S16 and S17), compared with 12.4 kJ mol^–1^ in the QN-derived host **4**, consistent with the much lower experimental enantioselectivity of the CN-derived sponge (*vide supra*). The superior performance of **4** therefore reflects not simply inversion of molecular chirality, but the cooperative consequence of molecular electrostatic topology and tighter supramolecular confinement.

Guest identity provides a second level of control. Relative to MeTHF, the carbonyl group of 3-MCH is more strongly polarized and sterically accessible, strengthening the orientational O···H/electrostatic interactions with the host. At the same time, the larger size of 3-MCH makes accommodation within the cavity more configuration-sensitive and increases the difference in repulsive contributions between the two enantiomers (Tables S20 and S21). Thus, the higher enantioselectivity observed in the uptake of 3-MCH compared with MeTHF arises from the simultaneous amplification of electrostatic orientation and steric discrimination, rather than from either factor alone. The resulting calculated energy discrimination is approximately 27.8 kJ mol^–1^ for **4**·(3-MCH), compared with 12.4 kJ mol^–1^ for **4**·MeTHF, paralleling the increase in experimental *ee* from 45% to 76% (*vide supra*). However, it should be emphasized that these are static interaction energies evaluated for fixed, crystallographically derived geometries of the two enantiomeric arrangements, and not free energies of the crystallization process, which additionally involves entropic contributions, solvation, and lattice dynamics. They are accordingly larger in absolute terms than the differences implied by the observed enantiomeric ratios (2.4 and 4.9 kJ mol^–1^ at 298 K for *ee* 45% and 76%, respectively)^41^ and should be read as a measure of the relative discrimination imposed by the adapted cavity rather than as a predictor of *ee*.

Taken together, the structural and computational results define a qualitative design framework for the substrate scope of the present crystalline sponge. Efficient enantiodiscrimination requires a guest large enough to experience meaningful confinement, yet compatible with adaptive adjustment of the cavity and whose two enantiomeric orientations differ sufficiently in steric fit. A weakly polar and sterically accessible heteroatom can further reinforce orientation through directional electrostatic contacts, while strong hydrogen bonding or ionic recognition motifs are not required. Conversely, guests that are too small to be effectively confined, too large to be accommodated without major framework disruption, or insufficiently differentiated in shape and electrostatic surface are expected to show a reduced selectivity for a given host architecture. Importantly, these boundaries define a tunable design space rather than an intrinsic limitation of the concept. Systematic variation of the chiral host, including other cinchona alkaloid derivatives and structurally diverse chiral O,N-donor ligands with modified carbon frameworks, should allow cavity dimensions, shape, and electrostatic topology to be matched to different guest classes. Thus, the present work is best viewed not as defining a single universal sponge but as establishing design principles for families of adaptive chiral hosts targeted to weakly interacting substrates.

### Guest Recovery and Host Recyclability

The recovery of enantiomerically enriched guests from crystalline sponges by thermal vacuum-assisted desorption may pose challenges for large-scale separation processes, particularly in the case of higher-boiling guests such as 2- and 3-MCH. Therefore, we developed an alternative, potentially scalable batch recovery approach based on the dissolution of solvated crystals in MeCN, followed by extraction of the enantiomerically enriched guest into hexane (Figure 6b). This approach enables the efficient recovery of MeTHF, 2-MCH, and 3-MCH from the host matrices while maintaining the enantiomeric excess observed in the initial studies (*vide supra*). Note that the procedure was ineffective for GVL because of its immiscibility with hexane. The enantioenriched guests are obtained directly as easy-to-handle solutions in hexane. The scalability of this approach was demonstrated by the preparation of approximately 10 mL of a 0.14 M solution of the enantiomerically enriched 2-MCH (*ee* = 68%) (Table 2 and Figure S24).

**Figure 6:**
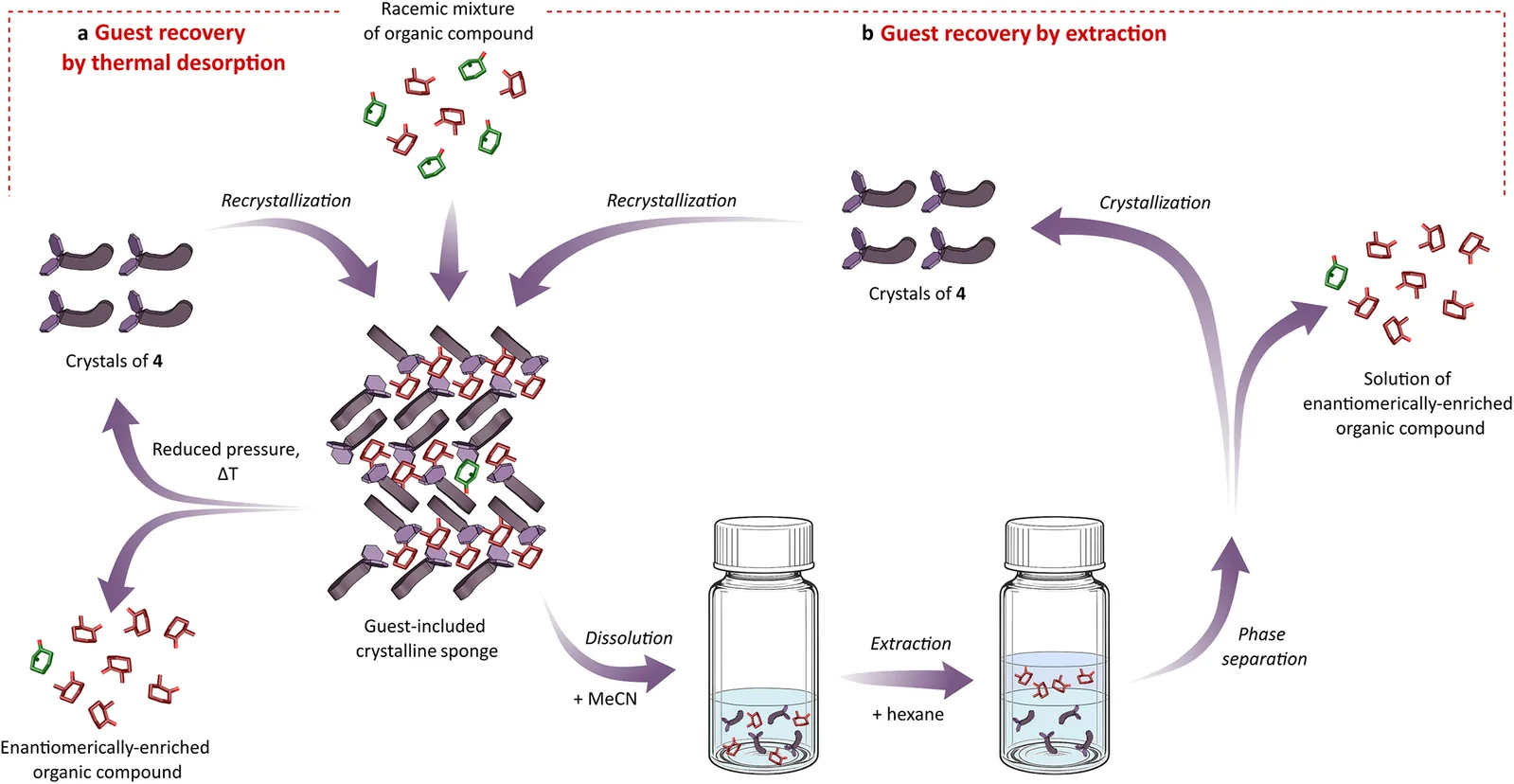
Schematic representation of two pathways for the recovery of an enantiomerically enriched guest: (a) thermal desorption and (b) extraction.

**Table 2:** Summary of Guest Recovery Experiments Using the Extraction Procedure

Host–guest system	Guest loading [%]^a^	Initial sample mass [mg]	Crystal mass [mg]	Crystallization efficiency [%]	Extract volume	Guest concentration in extract [mol·dm^–3^]	Guest recovery [%]	Guest *ee* [%]
**2**·0.5MeTHF	8.6	200	183	92	3 × 1 mL	0.05	82	14
**4**·MeTHF	14.9	200	175	88	3 × 1 mL	0.09	84	45
**4**·(2-MCH)	20.8	200	178	89	3 × 1 mL	0.07	64	68
**4**·(3-MCH)	19.8	200	164	82	3 × 1 mL	0.08	82	76
Scale-up experiment								
**4**·(2-MCH)	20.8	1200	1000	83	2 × 5 mL	0.14	78	68
Reproducibility experiment								
**4**·MeTHF								
Cycle 1	14.9	500	468	94	2 × 1 mL	0.31	77	45
Cycle 2	14.9		293	59	2 × 1 mL	0.20	78	38
Cycle 3	14.9		382	76	2 × 1 mL	0.28	84	31
Cycle 4	14.9		450	90	2 × 1 mL	0.26	66	35
Cycle 5	14.9		213	43	2 × 1 mL	0.15	81	45
Cycle 6	14.9		312	62	2 × 1 mL	0.21	77	43
Cycle 7	14.9		289	58	2 × 1 mL	0.19	75	46
Cycle 8	14.9		295	59	2 × 1 mL	0.19	74	45

a Based on TGA, see Table S11.

Importantly, this approach allows the host molecules to be readily recovered from the MeCN phase and reused in subsequent cycles of the separation process. To directly assess host recyclability, we performed a multicycle experiment using **4**·MeTHF. Following the initial crystallization of **4**·MeTHF from a racemic MeTHF mixture, the guest was recovered using the extraction procedure described above. The resulting MeCN phase containing the host was then dried under vacuum, and the recovered host was reused by returning it to the parent crystallization solution. In this way, the host material can be continuously recycled and kept in circulation throughout successive separation cycles, without the need for replenishment. The process was repeated for eight consecutive cycles, with crystallization efficiency ranging from 43% to 94%, guest recovery from 66% to 84%, and MeTHF *ee* maintained in the range of 31–46% (Table 2 and Figure S25). These results demonstrate that the enantioseparation performance remains reproducible over multiple recovery and recrystallization cycles, highlighting the potential of the approach for repeated use rather than being limited to a single batch of crystalline host material.

During these experiments, we also isolated single crystals of a new **4**·MeCN solvate suitable for SC-XRD analysis. The crystal structure revealed the same host framework observed in the other solvates, with MeCN molecules occupying the available voids (for details, see SI). The MeCN molecules adopt an arrangement analogous to that of the guests in the other **4**-based solvates and are directed by similar weak intermolecular C–H···N interactions. This structure not only confirms that the organoboron building block remains chemically intact following guest recovery but also suggests that nitrile groups may contribute to guest recognition in a manner analogous to the carbonyl and ether functionalities observed for other guests. The isolation of structurally well-defined **4**·MeCN crystals also provides further direct crystallographic evidence for both the robustness of the molecular building block and the adaptive character of its supramolecular assembly. Together with the recycling experiments, these results demonstrate that the host can withstand the extraction procedure and subsequently re-enter the guest inclusion and separation cycle.

Although the present implementation relies on a batch crystallization process, the successful scale-up and recyclability experiments demonstrate the potential of the approach for larger-scale operations. Continuous processing should be feasible in principle, although its implementation would require further development and optimization of nucleation, crystal growth, and residence time control, which are beyond the scope of the present study.

## Conclusions

In conclusion, we demonstrate that adaptive supramolecular crystalline sponges assembled from cinchona alkaloid-derived organoboron complexes provide an effective platform for the enantioseparation of small, weakly interacting organic molecules. Unlike conventional crystallization-based approaches, which rely on strong directional interactions, the present system operates through guest inclusion within homochiral cavities, where adaptive confinement and cooperative weak host–guest interactions enable efficient enantiodiscrimination. The ability to resolve challenging liquid substrates such as γ-valerolactone and methylcyclohexanone isomers, which are typically accessed in enantiopure form only *via* asymmetric synthesis, highlights the unique capability of this approach to address a long-standing limitation in enantioseparation. Structural analysis reveals that enhanced selectivity arises from a combination of geometric complementarity, framework adaptability, and modulation of cavity volume, providing mechanistic insight into chiral recognition in weakly interacting systems.

These findings establish adaptive confinement in supramolecular crystalline sponges as a general strategy for enantioseparation beyond conventional crystallization paradigms and open new opportunities for the resolution of otherwise inaccessible chiral molecules in the solid state. More broadly, this work suggests that dynamic supramolecular environments can be harnessed to achieve selective molecular recognition, even in the absence of strong binding motifs.

## Supplementary Material



## Notes

Materials, synthesis and characterization methods, experimental procedures, SC-XRD, NMR, TGA, PXRD, and GC data (PDF)

## ∥

I.J. Deceased on July 29, 2026
