# Supplementary material for: Adaptive Cinchona Alkaloid-Derived Organoboron Crystalline Sponges Enable Enantioseparation of Weakly Interacting Molecules
Source: J Am Chem Soc. 2026 Oct 2;148(42):45445–56. doi: 10.1021/jacs.6c09766 (PMC13721035; doi:10.1021/jacs.6c09766)
Supplement: Supplementary file 1 [file ja6c09766_si_001.pdf]

Supporting Information

Adaptive Cinchona-Alkaloid-derived Organoboron Crystalline Sponges Enable Enantioseparation of Weakly Interacting Molecules

Maximilian Rajski,[1‡] Michał Terlecki,[1‡] Iwona Justyniak,[2] Arkadiusz Kornowicz,[2] Michał K. Leszczyński,[1,2] Mikołaj Cieślak,[1] and Janusz Lewiński*[1,2]

[1]Faculty of Chemistry, Warsaw University of Technology, Noakowskiego 3, 00-664 Warsaw, Poland.

[2]Institute of Physical Chemistry, Polish Academy of Sciences, Kasprzaka 44/52, 01-224, Warsaw, Poland.

*Correspondence to Janusz Lewiński (janusz.lewinski@pw.edu.pl)

Table of contents

1. Experimental Section S2
2. Single Crystal X-Ray Diffraction S5
3. NMR Spectra S18
4. TGA S21
5. PXRD S23
6. Gas Chromatography S25
7. Hirshfeld surface analysis and interaction energy calculations S27

1. Experimental Section

General remarks. The synthesis of organoboron complexes was conducted using the standard Schlenk technique or in a glovebox under argon atmosphere. Reagents: BEt_3 (ABCR), BPh_3 (Thermo-Scientific), CN-H (Sigma-Aldrich), and QN-H (Sigma-Aldrich) and racemic mixtures of Me-THF (Sigma-Aldrich), GVL (Sigma-Aldrich), 2-MCH (Acros Organics), and 3-MCH (TCI chemicals) were purchased from commercial vendors and used as received. Solvents were degassed with nitrogen, dried over activated aluminium oxide (MBraun SPS) and stored over 3Å molecular sieves. Deuterated solvents were dried over Na/K alloy and distilled under argon atmosphere.

IR spectroscopy. Solid state FTIR spectra were measured with a Bruker Tensor II spectrometer using the ATR technique.

Elemental analysis. Elemental CHN analyses were performed using a UNICUBE elemental analyzer (Elementar Analysensysteme GmbH).

TGA analysis. TGA was carried out with a TA Instruments Q600 instrument under an Ar flow (flow rate of 100 mL min^{-1}) to 600 °C at a heating rate of 5 °C min^{-1}.

Powder X-ray diffraction. PXRD data were collected with an Empyrean diffractometer (PANalytical) by employing Ni-filtered CuKα radiation from a copper sealed tube charged with 40 kV voltage and 40 mA current in Bragg–Brentano geometry with a beam divergence of 1° in the scattering plane.

Gas chromatography. Enantiomeric excesses were determined by chiral gas chromatography analyses performed on a GC Shimadzu Nexis GC-2030 instrument equipped with a gas chromatography (flame ionization detector) and chiral column: Mega-Dex DMT Beta for MeTHF and GVL or Supelco Alpha DEX 120 for 2-MCH and 3-MCH.

Synthesis and characterization:

Synthesis of 1. A solution of BEt_3 in hexane (2 M, 1 mL, 2 mmol) was added to a suspension of *CN*-H (588 mg, 2 mmol) in toluene (10 mL). The reaction mixture was stirred at 110°C overnight. All attempts to obtain crystals of **1** from toluene, THF, or MeTHF solutions resulted in amorphous white precipitate or slurries. Therefore, the product was isolated by removal of the solvent under reduced pressure and characterized spectroscopically. ^{1}H NMR (d_8-DMSO, 600 MHz): δ = 8.92 (d, 1H, Ar^{CN}), 8.04 (d, 1H, Ar^{CN}), 7.92 (d, 1H, Ar^{CN}), 7.87 (d, 1H, Ar^{CN}), 7.73 (m, 1H, Ar^{CN}), 7.56 (m, 1H, Ar^{CN}), 5.72 (d, 1H, OCH), 5.16 (m, 1H, =C(R)H), 4.77 (d, 1H, $=CH_2$), 4.74 (d, 1H, $=CH_2$), 4.24 (m, 1H, quinuclidine-H), 3.06 (m, 2H, quinuclidine-H), 2.80 (m, 1H, quinuclidine-H), 2.73 (m, 1H, quinuclidine-H), 2.38 (m, 1H, quinuclidine-H), 1.63 (m, 1H, quinuclidine-H), 1.58 (m, 1H, quinuclidine-H), 1.31 (m, 1H, quinuclidine-H), 0.87 (m, 3H, CH_3), 0.81 (m, 3H, CH_3), 0.7-0.2 (m, 6H, quinuclidine-H + B-CH_2); ^{11}B NMR (d_8-DMSO, 192.5 MHz): δ = 9.03; FTIR (ATR, cm^{-1}): 3072 (w), 2946 (s), 2905 (m), 2868 (s), 2828 (w), 1633 (w), 1592 (s), 1568 (w), 1509 (s), 1455 (s), 1428 (w), 1357 (m), 1293 (m), 1269 (m), 1242 (m), 1198 (w), 1163 (w), 1113 (s), 1076 (s), 1030 (s), 1004 (m), 953 (w), 919 (s), 888 (m), 842 (s), 801 (m), 771 (s), 730 (s), 680 (m), 605 (w).

Synthesis of 2·0.5THF. BPh_3 (484 mg, 2 mmol) was added to a suspension of *CN*-H (588 mg, 2 mmol) in THF (10 mL). The reaction mixture was stirred at 60°C overnight. The product was isolated as colorless crystals after crystallization from a concentrated parent solution at -20°C (yield = 92%, 880 mg). ^{1}H NMR (d_8-DMSO, 600 MHz): δ = 9.02 (d, 1H, Ar^{CN}), 8.14 (d, 1H, Ph), 8.09 (d, 1H, Ar^{CN}), 7.96 (d, 1H, Ar^{CN}), 7.87 (m, 2H, Ph+Ar^{CN}), 7.78 (m, 2H, Ph+Ar^{CN}), 7.57 (m, 1H, Ar^{CN}), 7.37 (m, 1H, Ph), 7.30 (t, 2H, Ph), 7.26

(t, 2H, Ph), 7.15 (m, 2H, Ph), 6.35 (d, 1H, OCH), 4.91 (m, 1H, =C(R)H), 4.63 (d, 1H, $=CH_2$), 4.55 (d, 1H, $=CH_2$), 3.99 (m, 1H, quinuclidine-H), 3.40 (m, 1H, quinuclidine-H), 2.90 (m, 1H, quinuclidine-H), 2.59 (m, 1H, quinuclidine-H), 2.32 (m, 1H, quinuclidine-H), 1.65 (m, 2H, quinuclidine-H), 1.56 (m, 1H, quinuclidine-H), 1.50 (m, 1H, quinuclidine-H), 0.64 (m, 1H, quinuclidine-H), 0.41 (m, 1H, quinuclidine-H); ^{11}B NMR (d_8-DMSO, 192.5 MHz): δ = 6.43; FTIR (ATR, cm^{-1}): 3064 (m), 3041 (m), 3003 (m), 2963 (m), 2944 (m), 2873 (m), 2851 (m), 1648 (w), 1593 (s), 1570 (m), 1510 (s), 1488 (w), 1461 (m), 1432 (s), 1361 (s), 1180 (m), 1149 (s), 1112 (s), 1078 (s), 1042 (m), 1003 (m), 958 (s), 897 (m), 878 (m), 844 (m), 805 (s), 756 (s), 732 (s), 707 (s), 646 (s), 610 (s), 585 (m). Elemental analysis (%) calcd for **2**·0.3THF ($C_{32.2}H_{33.4}BN_2O_{1.3}$, M = 480.0 g·mol^{-1}): C 80.57, H 7.01, N 5.84; found: C 80.65, H 7.08, N 5.89.

Synthesis of 3·THF. A solution of BEt_3 in hexane (2 M, 1 mL, 2 mmol) was added to a suspension of Q*N*-H (648 mg, 2 mmol) in toluene (10 mL). The reaction mixture was stirred at 110°C overnight. Then, the solvent was removed under reduced pressure, and the residue was dissolved in THF. The product was isolated as colorless crystals after crystallization from a concentrated THF solution at -20°C (yield = 89%, 830 mg). ^{1}H NMR (d_8-DMSO, 600 MHz): δ = 8.75 (d, 1H, Ar^{QN}), 7.95 (d, 1H, Ar^{QN}), 7.80 (d, 1H, Ar^{QN}), 7.37 (m, 1H, Ar^{QN}), 7.13 (m, 1H, Ar^{QN}), 6.04 (m, 1H, =C(R)H), 5.70 (d, 1H, OCH), 5.09 (d, 1H, $=CH_2$), 5.07 (d, 1H, $=CH_2$), 4.28 (m, 1H, quinuclidine-H), 3.91 (s, 3H, OCH_3), 3.02 (m, 1H, quinuclidine-H), 2.94 (m, 1H, quinuclidine-H), 2.86 (m, 2H, quinuclidine-H), 2.42 (m, 1H, quinuclidine-H), 1.70 (m, 1H, quinuclidine-H), 1.63 (m, 1H, quinuclidine-H), 1.46 (m, 1H, quinuclidine-H), 1.15 (m, 1H, quinuclidine-H), 0.84 (m, 6H, CH_3), 0.6-0.3 (m, 5H, quinuclidine-H + B-CH_2); ^{11}B NMR (d_8-DMSO, 192.5 MHz): δ =9.34; FTIR (ATR, cm^{-1}): 3070 (w), 2944 (s), 2909 (m), 2862 (m), 2834 (m), 1621 (s), 1591 (m), 1502 (s), 1470 (m), 1454 (m), 1430 (s), 1355 (m), 1292 (m), 1276 (m), 1243 (m), 1227 (s), 1201 (m), 1103 (m), 1070 (s), 1054 (s), 1023 (s), 988 (w), 918 (s), 890 (m), 848 (s), 825 (m), 795 (w), 764 (w), 722 (w), 687 (w), 654 (w), 615 (m), 584 (w), 559 (m). Elemental analysis (%) calcd for **3**·1.0THF ($C_{28}H_{41}BN_2O_3$, M = 464.5 g·mol^{-1}): C 72.41, H 8.90, N 6.03; found: C 72.40, H 8.95, N 5.99.

Synthesis of 4·THF. BPh_3 (484 mg, 2 mmol) was added to a suspension of *QN*-H (648 mg, 2 mmol) in THF (10 mL). The reaction mixture was stirred at 60°C overnight. The product was isolated as colorless crystals after crystallization from a concentrated parent solution at -20°C (yield = 88%, 1000 mg). ^{1}H NMR (d_8-DMSO, 600 MHz): δ = 8.83 (d, 1H, Ar^{QN}), 8.07 (d, 1H, Ph), 8.00 (d, 1H, Ar^{QN}), 7.88 (d, 1H, Ar^{QN}), 7.78 (m, 2H, Ph), 7.41 (m, 1H, Ar^{QN}), 7.32 (m, 1H, Ph), 7.28 (t, 2H, Ph), 7.26 (t, 2H, Ph), 7.17 (m, 1H, Ar^{QN}), 7.14 (m, 2H, Ph), 6.26 (d, 1H, OCH), 5.91 (m, 1H, =C(R)H), 4.99 (d, 1H, $=CH_2$), 4.96 (d, 1H, $=CH_2$), 4.07 (m, 1H, quinuclidine-H), 3.91 (s, 3H, OCH_3), 3.77 (m, 1H, quinuclidine-H), 3.22 (m, 1H, quinuclidine-H), 2.86 (m, 1H, quinuclidine-H), 2.71 (m, 1H, quinuclidine-H), 2.53 (m, 1H, quinuclidine-H), 1.89 (m, 1H, quinuclidine-H), 1.65 (m, 1H, quinuclidine-H), 1.45 (m, 1H, quinuclidine-H), 0.89 (m, 1H, quinuclidine-H), 0.62 (m, 1H, quinuclidine-H); ^{11}B NMR (d_8-DMSO, 192.5 MHz): δ = 6.84; FTIR (ATR, cm^{-1}): 3064 (m), 3049 (m), 2990 (w), 2963 (m), 2934 (m), 2880 (m), 1622 (s), 1588 (m), 1570 (m), 1510 (s), 1473 (s), 1429 (s), 1356 (m), 1287 (w), 1263 (w), 1239 (s), 1225 (s), 1173 (w), 1149 (s), 1112 (s), 1083 (s), 1056 (s), 1022 (s), 910 (s), 897 (m), 871 (s), 800 (m), 805 (s), 734 (s), 707 (s), 617 (s), 583 (m). Elemental analysis (%) calcd for **4**·1.1THF ($C_{36.4}H_{41.8}BN_2O_{3.1}$, M = 567,7 g·mol^{-1}): C 77.01, H 7.42, N 4.93; found: C 77.01, H 7.43, N 4.92.

Enantioseparation process:

Inclusion of small chiral molecules in crystalline sponges. In a typical experiment, the organoboron complex (THF solvate of the dried material) was dissolved in a racemic mixture of a selected chiral compound (MeTHF, GVL, 2-MCH, or 3-MCH). Crystals containing the chiral guest were obtained by

crystallization from a concentrated parent solution at -20°C, then washed with hexane and gently dried under reduced pressure. This procedure enabled efficient preparation of **2**·0.5MeTHF, **4**·MeTHF, **4**·GVL, **4**·(2-MCH), and **4**·(3-MCH) in 60–95% yield. In the case of complex **3**, recrystallization from MeTHF afforded a crystalline powder unsuitable for SCXRD studies, and GC and TGA analyses indicated no inclusion of MeTHF within the framework of the resulting product (see, Fig. S17b and S22). The encapsulated guests were easily recovered either by thermal desorption or extraction, and the *ee* values were determined by GC analysis (Fig. 3).

Guest recovery by thermal desorption. In a typical experiment, crystals containing the chiral guest were loaded into a distillation setup and heated (120-200°C) under reduced pressure, while the volatiles were collected in a liquid nitrogen trap. This procedure enabled quantitative recovery of the chiral guest compounds, which were subsequently characterized by GC. The residual crystalline sponge was typically suitable for reuse; however, in the case of GVL, 2-MCH, or 3-MCH solvates, the higher temperatures required for guest removal may lead to partial decomposition of the organoboron complexes.

Guest recovery by extraction.
In a typical experiment, crystals containing the chiral guest (200 mg) were dissolved in MeCN (1 mL). The resulting solution was extracted three times with 1 mL portions of hexane, affording ca. 3 mL of an enantioenriched guest solution in hexane, which was subsequently analyzed by GC. This method was effective for **2**·MeTHF, **4**·MeTHF, **4**·(2-MCH), and **4**·(3-MCH), allowing recovery of the guest compounds with c.a. 60-85% efficiency, but is unsuitable for **4**·GVL due to the poor solubility of GVL in hexane.

Scale-up experiment. Crystals of **4**·(2-MCH) (1 g) were dissolved in MeCN (4 mL). The resulting solution was extracted twice with 5 mL portions of hexane, affording ca. 10 mL of an enantioenriched (ee ≈ 68%) solution of 2-MCH/hexane at a concentration of 0.14 M, as confirmed by GC analysis (Fig. S23).

Reproducibility experiment. Crystals of **4**·THF (500 mg) were dissolved in a racemic mixture of MeTHF (4 mL) and crystalized at -20°C. The resulting crystals were separated from the parent liquid, washed with hexane, and gently dried under reduced pressure. The as-received crystals of **4**·MeTHF were then dissolved in MeCN (1 mL) and extracted twice with 1 mL portions of hexane, affording ca. 2 mL of an enantioenriched MeTHF/hexane solution, which was analyzed by GC (Fig. Sx). The MeCN fraction was dried under reduced pressure and the recovered host material was returned to the parent racemic MeTHF solution. The cycle was repeated eight times. The results are summarized in Table 2.

2. Single Crystal X-Ray Diffraction

The crystals were selected under Paratone-N oil, mounted on the nylon loops, and positioned in the cold stream on the diffractometer. The X-ray data for complexes **2**·0.5THF, **2**·0.5MeTHF, **3**·THF, **4**·THF, **4**·MeTHF, **4**·GVL, **4**·(2-MCH), **4**·(3-MCH), and **4**·MeCN were collected at 100(2) K on a SuperNova Agilent diffractometer using graphite monochromated Cu*Kα* (λ = 1.54184 Å) or Mo*Kα* (λ = 0.71073 Å) radiation. The data were processed with CrysAlisPro.[1] The crystal structures were solved by direct methods using the SHELXS-97[2] program and were refined by full-matrix least-squares on F^2 using the program SHELXL-program[3] implemented in the Olex2[4] or WinGX[5]. All non-hydrogen atoms were refined with anisotropic displacement parameters. Hydrogen atoms were added to the structure model at geometrically idealized coordinates and refined as riding atoms.

Crystallographic data (excluding structure factors) for the structure reported in this paper have been deposited with the Cambridge Crystallographic Data Centre as a supplementary publication. Copies of the data can be obtained free of charge on application to CCDC, 12 Union Road, Cambridge CB21EZ, UK (fax: (+44)1223-336-033; e-mail: deposit@ccdc.cam.ac.uk). CCDC: 2551577 (**2_THF**), 2551576 (**2_MeTHF**), 2551578 (**3_THF**), 2551584 (**4_THF**), 2551583 (**4_MeTHF**), 2551582 (**4_GVL**), 2551579 (**4_2MCH**), 2551580 (**4_3MCH**), 2580382 (**4_MeCN**),

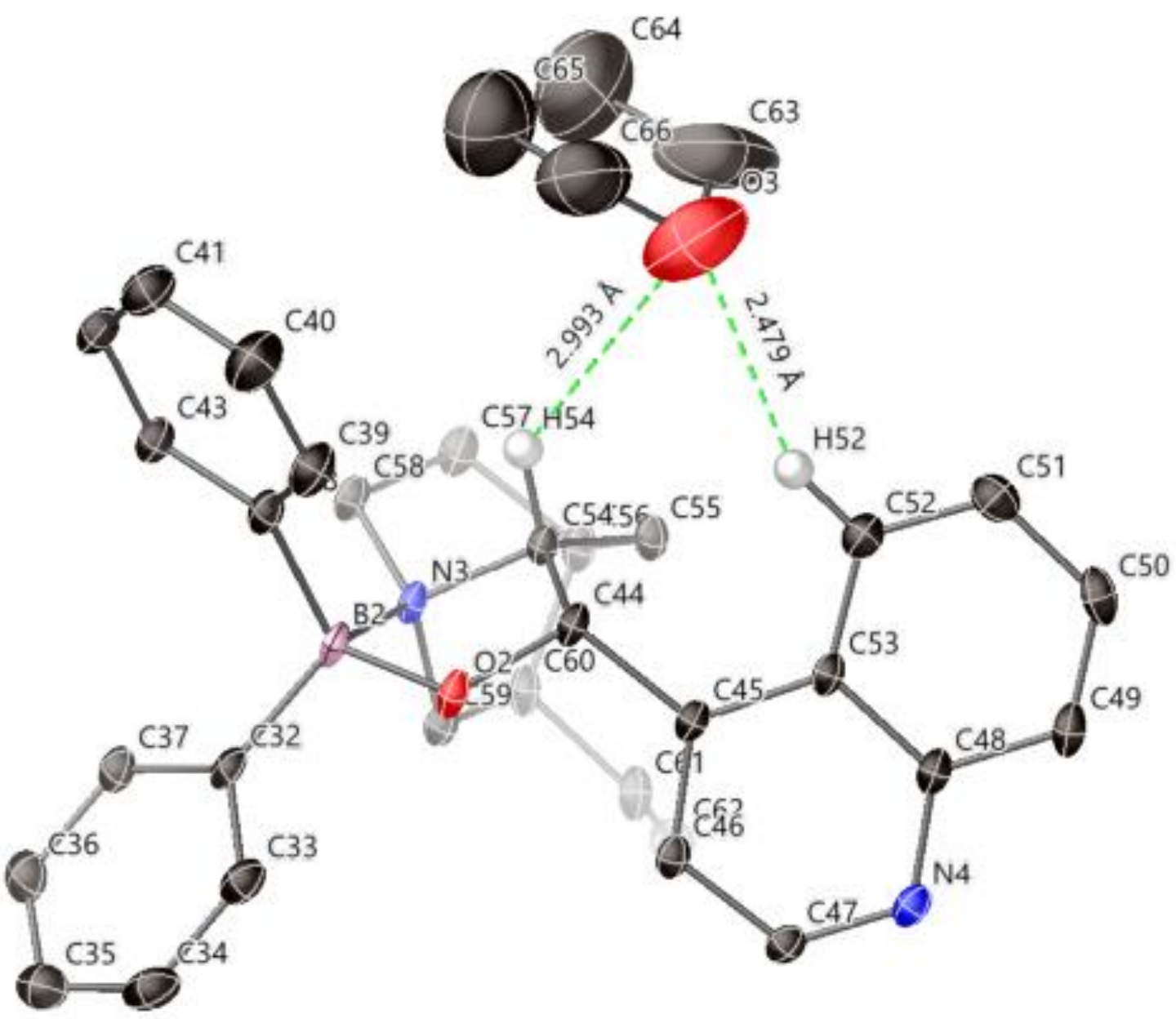

Fig. S1. The molecular structure of **2·0.5THF** with thermal ellipsoids set at 50% probability. Hydrogen atoms have been omitted for clarity.

Table S1. Crystallographic data and structure refinement parameters for **2·0.5THF.**

Empirical formula	$C_{66}H_{70}B_2N_4O_3$	
Formula weight	988.88	
Temperature	100(2) K	
Wavelength	1.54184 Å	
Crystal system	Orthorhombic	
space group	$P2_12_12_1$	
Unit cell dimensions	a = 11.42570(10) Å	α = 90°
	b = 17.8494(2) Å	β = 90°
	c = 25.8115(2) Å	γ = 90°
Volume	5264.05(9) $Å^3$	
Z	4	
Calculated density	1.248 Mg/m^3	
Absorption coefficient	0.583 mm^{-1}	
F(000)	2112	
Theta range for data collection	3.010 to 71.885 deg.	
Index ranges	-14<=h<=12, -20<=k<=21, -31<=l<=31	
Reflections collected	35384	
Independent reflections	10203 [*R(int)* = 0.0304]	
Completeness to theta = 67.684	100.0 %	
Refinement method	Full-matrix least-squares on F^2	
Data / restraints / parameters	10203 / 0 / 676	
Goodness-of-fit on F^2	1.040	
Final R indices [I>2sigma(I)]	R_1 = 0.0451, $wR2$ = 0.1208	
R indices (all data)	R_1 = 0.0459, $wR2$ = 0.1216	
Absolute structure parameter	0.03(7)	
Largest diff. peak and hole	0.538 and -0.498 e.$Å^{-3}$	

$R1 = \Sigma||Fo| - |Fc||/\Sigma|Fo|$. [b] $wR2 = [\Sigma w(Fo^2 - Fc^2)^2/\Sigma w(Fo^2)^2]^{1/2}$

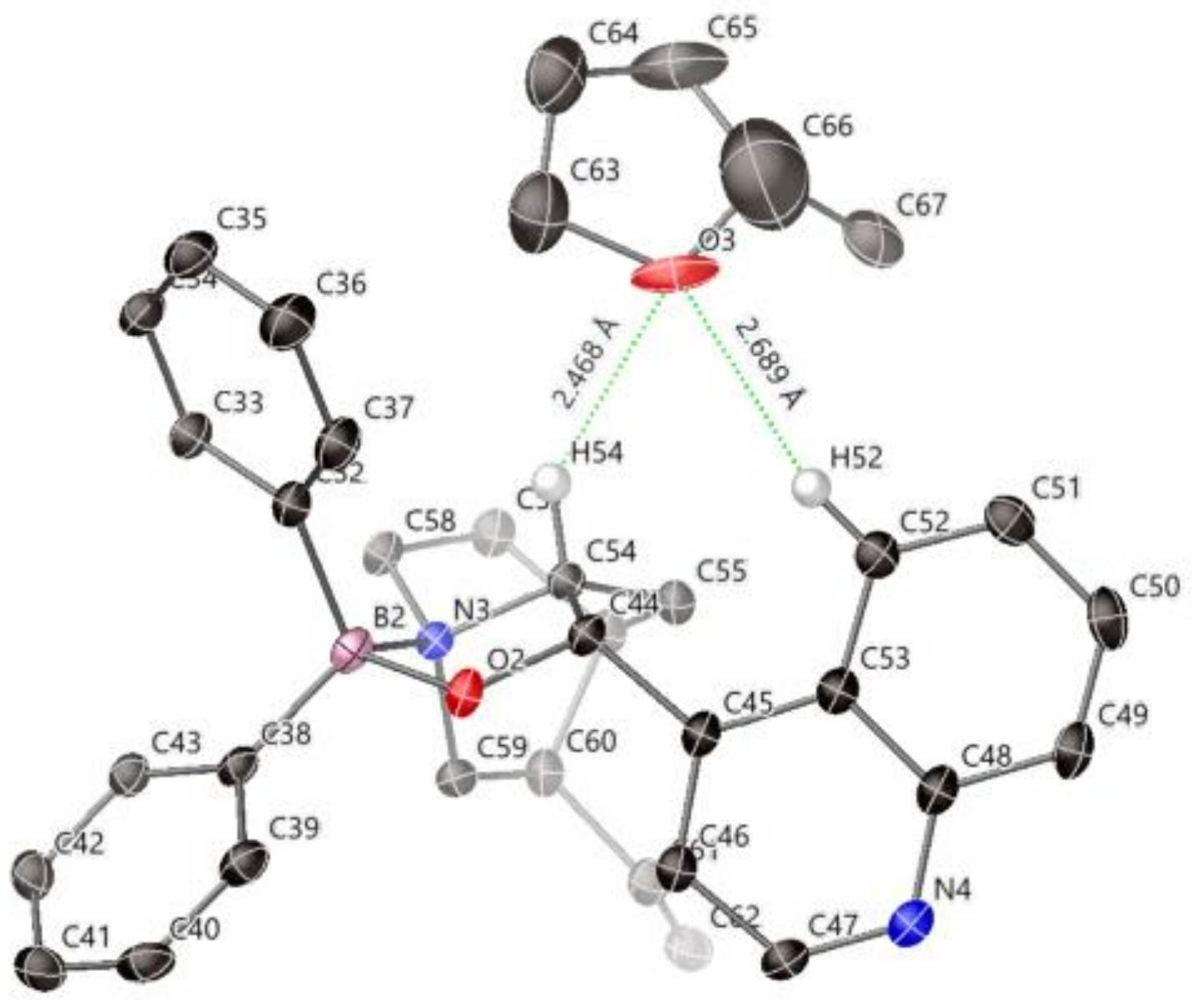

Fig. S2. The molecular structure of **2·0.5MeTHF** with thermal ellipsoids set at 50% probability. Hydrogen atoms have been omitted for clarity.

Table S2. Crystallographic data and structure refinement parameters for **2·0.5MeTHF.**

Empirical formula	$C_{130}H_{134}B_4N_8O_5$	
Formula weight	1931.68	
Temperature	100(2) K	
Wavelength	1.54184 Å	
Crystal system	Orthorhombic	
Space group	$P2_12_12_1$	
Unit cell dimensions	a = 11.45430(10) Å	α = 90°.
	b = 17.95500(10) Å	β = 90°.
	c = 25.7290(2) Å	γ = 90°.
Volume	5291.48(7) Å^3	
Z	2	
Density (calculated)	1.212 Mg/m^3	
Absorption coefficient	0.561 mm^{-1}	
F(000)	2060	
Theta range for data collection	4.579 to 71.511°.	
Index ranges	-13<=h<=9, -22<=k<=19, -31<=l<=31	
Reflections collected	34859	
Independent reflections	10205 [$R(int)$ = 0.0381]	
Completeness to theta = 67.684°	99.8 %	
Refinement method	Full-matrix least-squares on F^2	
Data / restraints / parameters	10205 / 91 / 686	
Goodness-of-fit on F^2	0.994	
Final R indices [I>2sigma(I)]	R_1 = 0.0535, wR_2 = 0.1479	
R indices (all data)	R_1 = 0.0591, wR_2 = 0.1509	
Absolute structure parameter	0.02(7)	
Largest diff. peak and hole	1.116 and -0.316 e.Å^{-3}	

$R1 = \Sigma||Fo| - |Fc||/\Sigma|Fo|$. [b] $wR2 = [\Sigma w(Fo^2 - Fc^2)^2/\Sigma w(Fo^2)^2]^{1/2}$

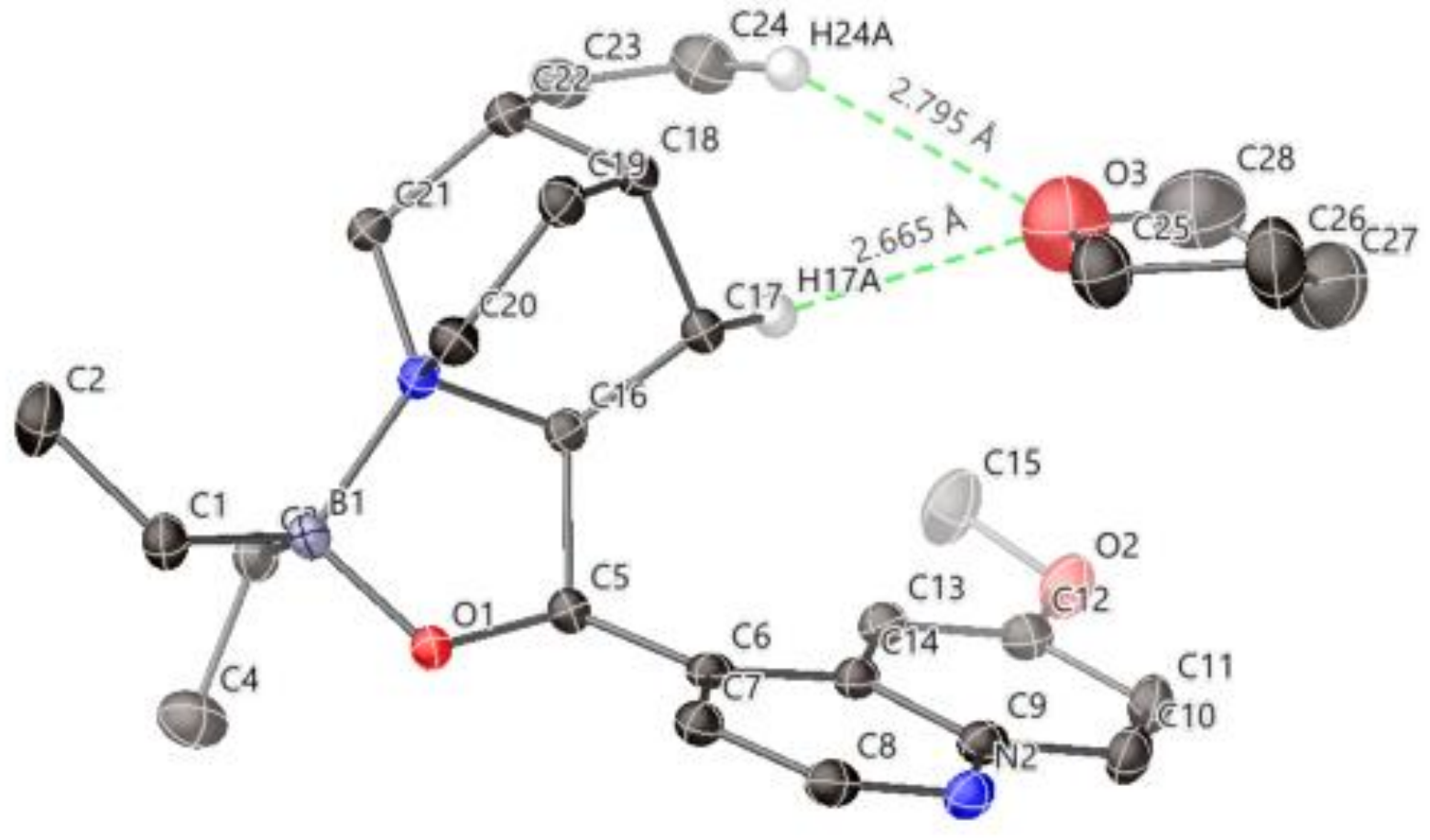

Fig. S3. The molecular structure of **3·THF** with thermal ellipsoids set at 50% probability. Hydrogen atoms have been omitted for clarity.

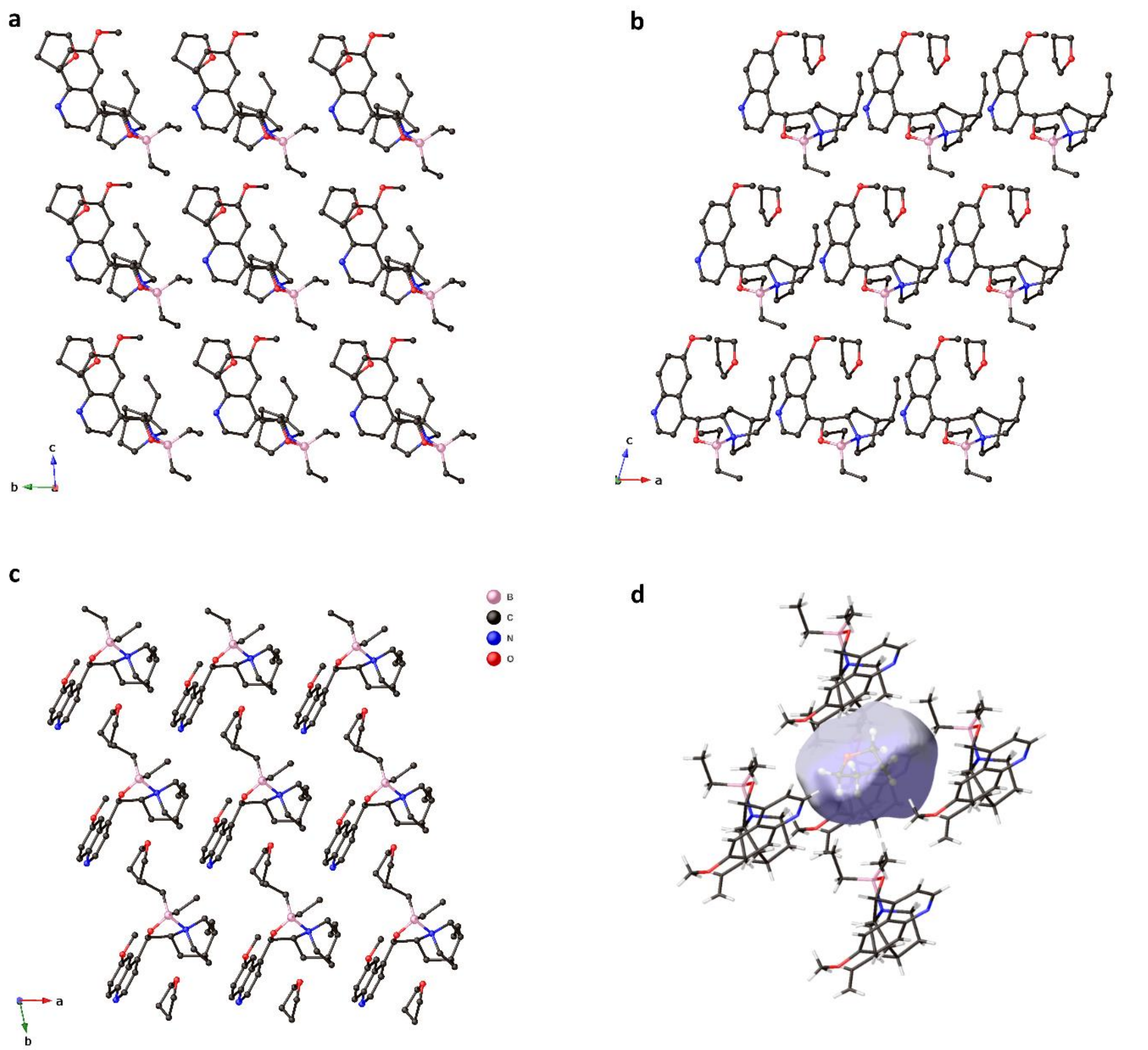

Fig. S4. Crystal packing of molecules in **3·THF** (a-c) and representation of guest occupied voids (d) [calculated as solvent-accessible voids (r = 1.4 Å); blue and gray colors denote the interior and exterior void surfaces, respectively].

Table S3. Crystallographic data and structure refinement parameters for **3 THF.**

Empirical formula	$C_{28}H_{41}BN_2O_3$	
Formula weight	464.44	
Temperature	100(2) K	
Wavelength	0.71073 Å	
Crystal system	Triclinic	
Space group	*P*1	
Unit cell dimensions	*a* = 7.9894(3) Å	*α* = 81.331(2)°
	b = 8.7633(2) Å	*β* = 72.609(3)°
	c = 9.9405(3) Å	*γ* = 75.132(3)°
Volume	639.85(4) $Å^3$	
Z	1	
Density (calculated)	1.205 Mg/m^3	
Absorption coefficient	0.077 mm^{-1}	
F(000)	252	
Theta range for data collection	2.741 to 25.658°.	
Index ranges	-8<=*h*<=9, -10<=*k*<=10, -11<=*l*<=12	
Reflections collected	8727	
Independent reflections	4240 [*R(int)* = 0.0121]	
Completeness to theta = 25.242°	99.7 %	
Refinement method	Full-matrix least-squares on F^2	
Data / restraints / parameters	4240 / 3 / 310	
Goodness-of-fit on F^2	1.056	
Final R indices [I>2sigma(I)]	R_1 = 0.0379, wR_2 = 0.1048	
R indices (all data)	R_1 = 0.0379, wR_2 = 0.1049	
Absolute structure parameter	-2.0(2)	
Largest diff. peak and hole	0.521 and -0.317 e.$Å^{-3}$	

$R1 = \Sigma||Fo| - |Fc||/\Sigma|Fo|$. [b] $wR2 = [\Sigma w(Fo^2 - Fc^2)^2/\Sigma w(Fo^2)^2]^{1/2}$

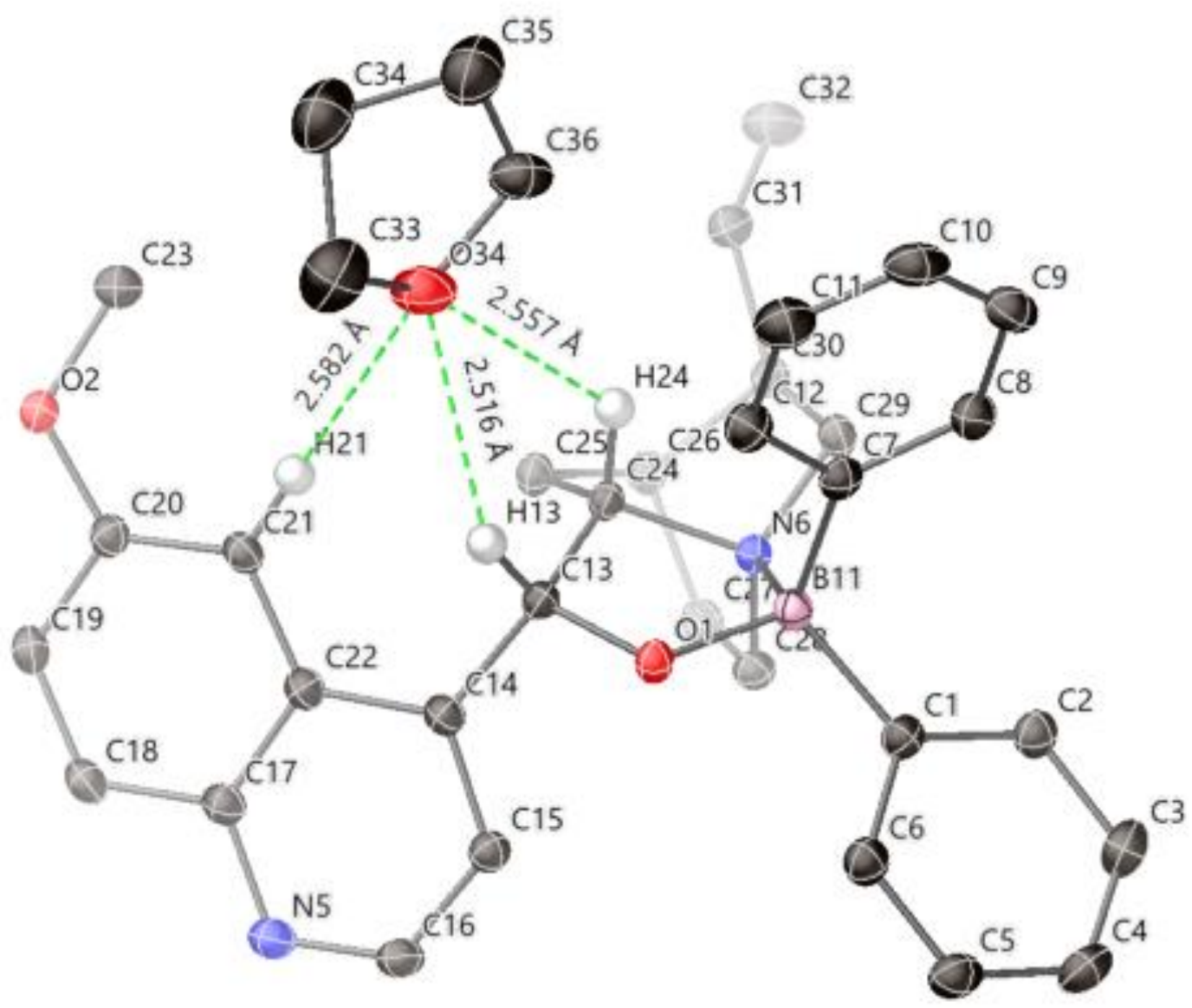

Fig. S5. The molecular structure of **4·THF** with thermal ellipsoids set at 50% probability. Hydrogen atoms have been omitted for clarity.

Table S4. Crystallographic data and structure refinement parameters for **4 THF.**

Empirical formula	$C_{36}H_{41}BN_2O_3$	
Formula weight	560.52	
Temperature	100(2) K	
Wavelength	1.54184 Å	
Crystal system	Orthorhombic	
Space group	$P2_12_12_1$	
Unit cell dimensions	a = 9.23320(10) Å	α = 90°
	b = 10.63330(10) Å	β = 90°
	c = 29.9767(2) Å	γ = 90°
Volume	2943.09(5) Å^3	
Z	4	
Density (calculated)	1.265 Mg/m^3	
Absorption coefficient	0.079 mm^{-1}	
F(000)	1200	
Theta range for data collection	2.9483 to 70.2109 °.	
Index ranges	-11<=h<=10, -12<=k<=12, -36<=l<=36	
Reflections collected	55697	
Independent reflections	5562 [$R(int)$ = 0.0422]	
Completeness to theta = 25.242°	99.7 %	
Refinement method	Full-matrix least-squares on F^2	
Data / restraints / parameters	5562 / 0 / 380	
Goodness-of-fit on F^2	0.925	
Final R indices [I>2sigma(I)]	R_1 = 0.0335, wR_2 = 0.0870	
R indices (all data)	R_1 = 0.0336, wR_2 = 0.0872	
Absolute structure parameter	-1.8(3)	
Largest diff. peak and hole	0.484 and -0.301 e.Å^{-3}	

$R1 = \Sigma||Fo| - |Fc||/\Sigma|Fo|$. [b] $wR2 = [\Sigma w(Fo^2 - Fc^2)^2/\Sigma w(Fo^2)^2]^{1/2}$

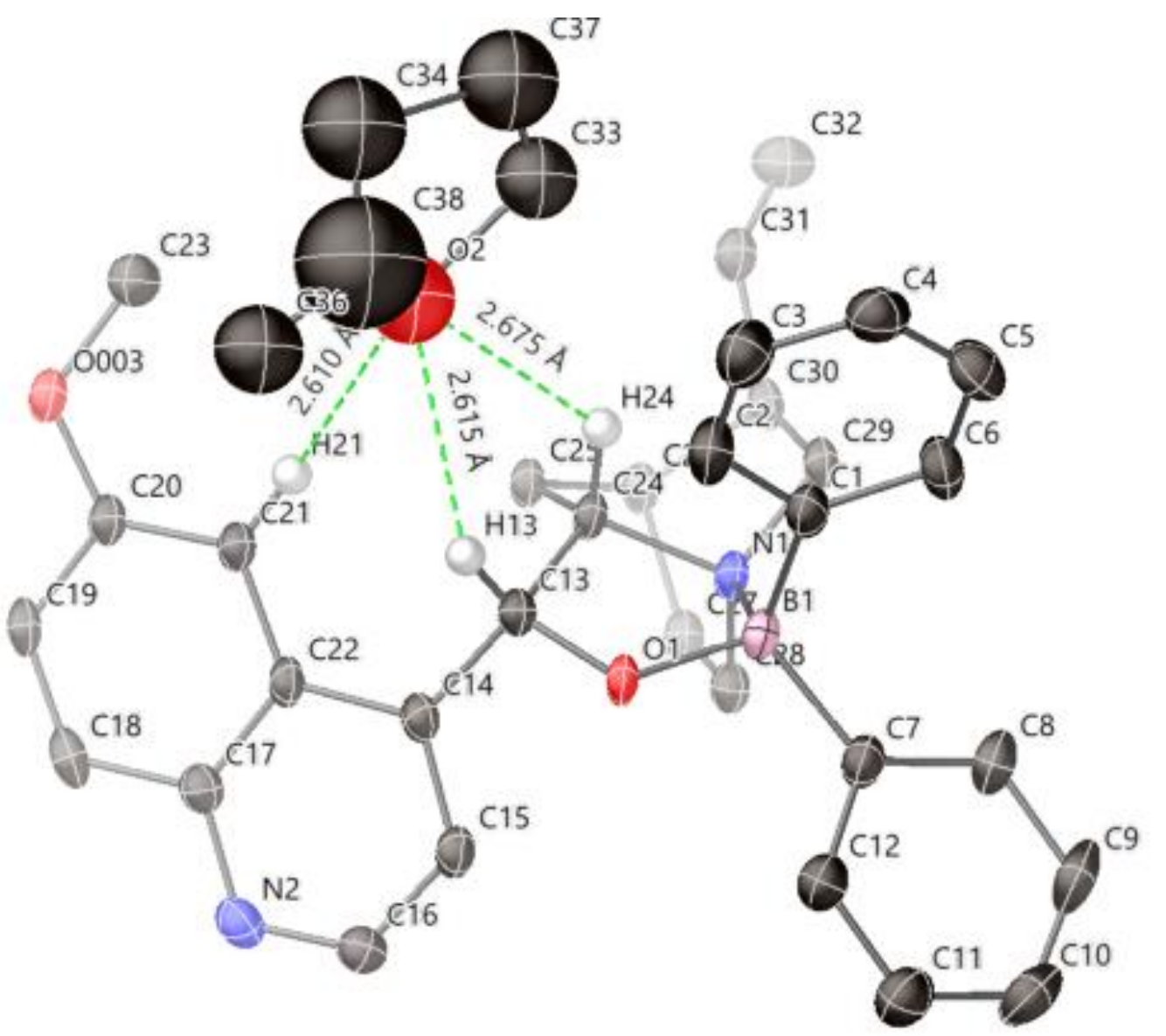

Fig. S6. The molecular structure of **4·MeTHF** with thermal ellipsoids set at 50% probability. Hydrogen atoms have been omitted for clarity.

Table S5. Crystallographic data and structure refinement parameters for **4 MeTHF.**

Empirical formula	$C_{37}H_{43}BN_2O_3$	
Formula weight	574.54	
Temperature	100(2) K	
Wavelength	1.54184 Å	
Crystal system	Orthorhombic	
Space group	$P2_12_12_1$	
Unit cell dimensions	a = 9.30077(6) Å	α = 90°
	b = 10.65954(8) Å	β = 90°
	c = 30.6359(2) Å	γ = 90°
Volume	3037.30(4) $Å^3$	
Z	4	
Density (calculated)	1.256 Mg/ m^3	
Absorption coefficient	0.613 mm^{-1}	
F(000)	1232	
Theta range for data collection	4.392 to 70.022°.	
Index ranges	-11<=h<=10, -12<=k<=12, -37<=l<=37	
Reflections collected	56824	
Independent reflections	5740 [R_{int} = 0.1382]	
Completeness to theta = 25.242°	99.8 %	
Refinement method	Full-matrix least-squares on F^2	
Data / restraints / parameters	5740 / 0 / 360	
Goodness-of-fit on F^2	1.035	
Final R indices [I>2sigma(I)]	R_1 = 0.0923, wR_2 = 0.2437	
R indices (all data)	R_1 = 0.0927, wR_2 = 0.2442	
Largest diff. peak and hole	1.356 and - -0.760 e.$Å^{-3}$	

$R1 = \Sigma||Fo| - |Fc||/\Sigma|Fo|$. [b] $wR2 = [\Sigma w(Fo^2 - Fc^2)^2/\Sigma w(Fo^2)^2]^{1/2}$

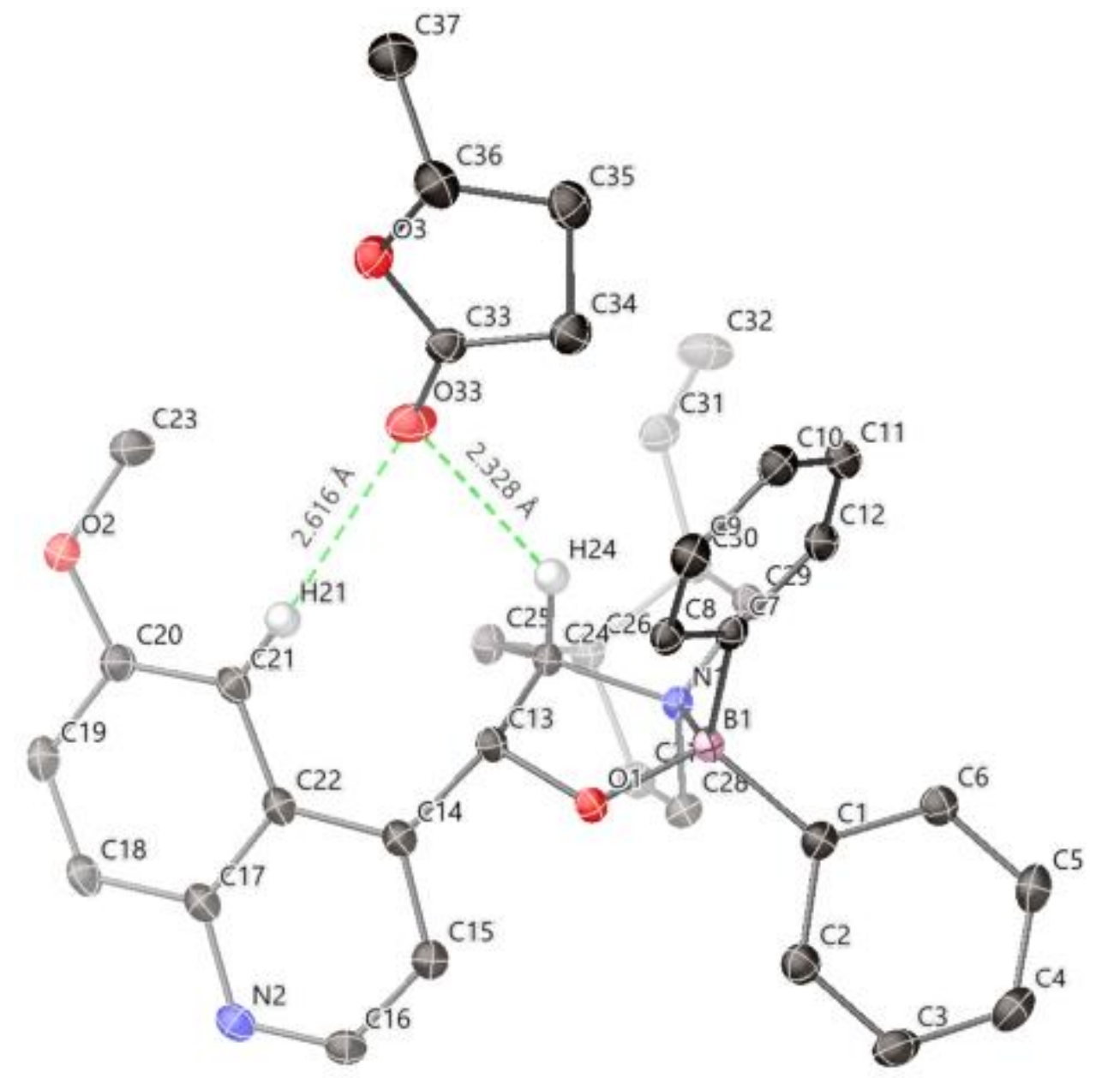

Fig. S7. The molecular structure of **4·GVL** with thermal ellipsoids set at 50% probability. Hydrogen atoms have been omitted for clarity.

Table S6. Crystallographic data and structure refinement parameters for **4 GVL.**

Empirical formula	$C_{37}H_{41}BN_2O_4$	
Formula weight	588.53	
Temperature	100(2) K	
Wavelength	1.54184 Å	
Crystal system	Orthorhombic	
Space group	$P2_12_12_1$	
Unit cell dimensions	a = 9.18310(10) Å	α = 90°.
	b = 10.71680(10) Å	β = 90°.
	c = 30.9233(2) Å	γ = 90°.
Volume	3043.27(5) $Å^3$	
Z	4	
Density (calculated)	1.285 Mg/m^3	
Absorption coefficient	0.651 mm^{-1}	
F(000)	1256	
Theta range for data collection	2.852 to 70.116°.	
Index ranges	-9<=h<=11, -13<=k<=13, -37<=l<=37	
Reflections collected	34706	
Independent reflections	5759 [$R(int)$ = 0.0324]	
Completeness to theta = 67.684°	100.0 %	
Refinement method	Full-matrix least-squares on F^2	
Data / restraints / parameters	5759 / 0 / 399	
Goodness-of-fit on F^2	1.034	
Final R indices [I>2sigma(I)]	R_1 = 0.0279, wR_2 = 0.0715	
R indices (all data)	R_1 = 0.0286, wR_2 = 0.0719	
Absolute structure parameter	0.00(4)	
Largest diff. peak and hole	0.266 and -0.225 e.$Å^{-3}$	

$R1 = \Sigma||Fo| - |Fc||/\Sigma|Fo|$. [b] $wR2 = [\Sigma w(Fo^2 - Fc^2)^2/\Sigma w(Fo^2)^2]^{1/2}$

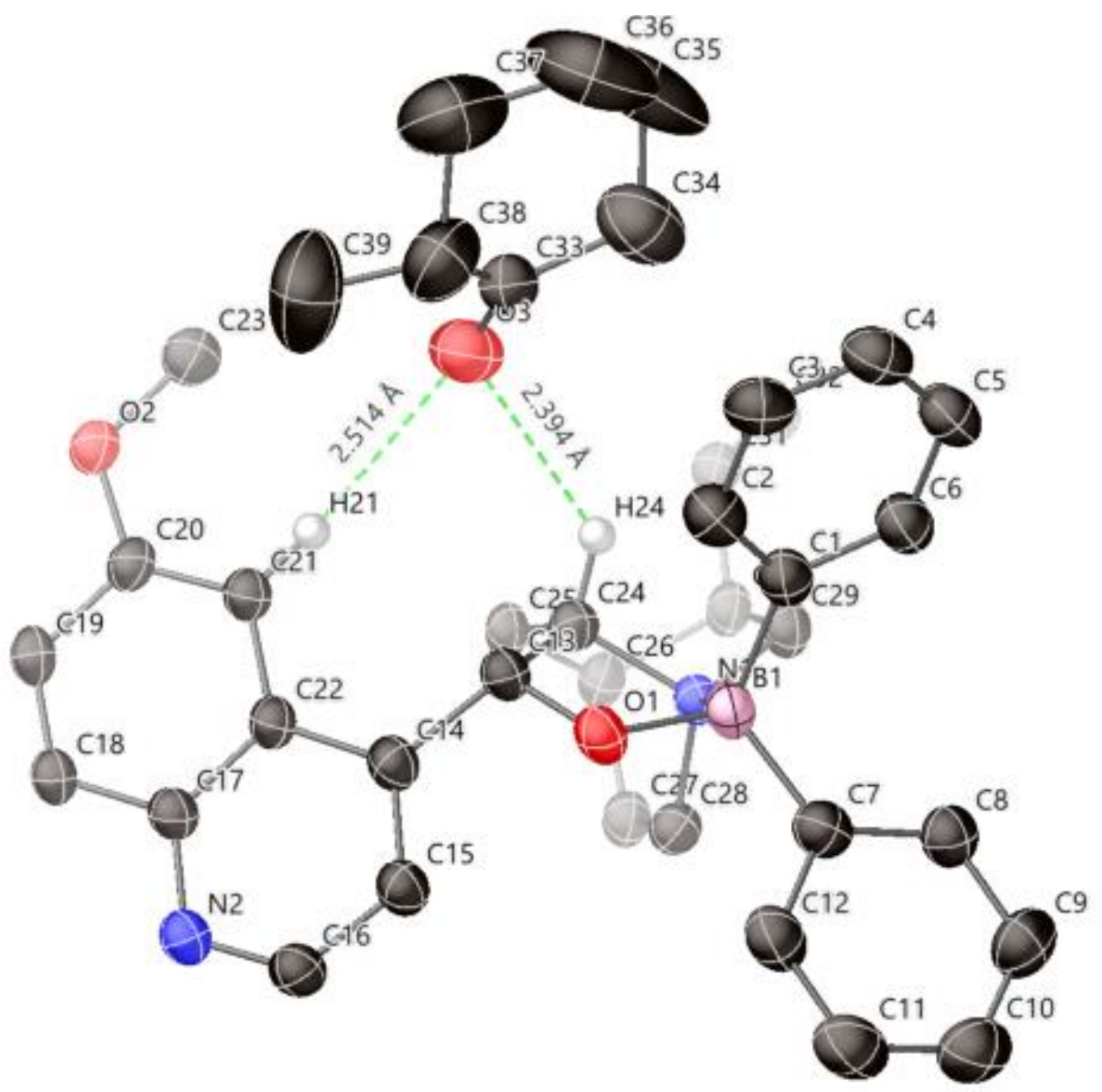

Fig. S8. The molecular structure of **4·(2-MCH)** with thermal ellipsoids set at 50% probability. Hydrogen atoms have been omitted for clarity.

Table S7. Crystallographic data and structure refinement parameters for **4 (2MCH)**

Empirical formula	$C_{39}H_{45}BN_2O_3$	
Formula weight	600.58	
Temperature	100(2) K	
Wavelength	1.54184 Å	
Crystal system	Orthorhombic	
Space group	$P2_12_12_1$	
Unit cell dimensions	a = 9.3880(2) Å	α = 90°.
	b = 10.5294(2) Å	β = 90°.
	c = 32.6461(10) Å	γ = 90°.
Volume	3227.07(14) $Å^3$	
Z	4	
Density (calculated)	1.236 Mg/m^3	
Absorption coefficient	0.599 mm^{-1}	
F(000)	1288	
Theta range for data collection	4.412 to 70.149°.	
Index ranges	$-11<=h<=10$, $-12<=k<=12$, $-39<=l<=39$	
Reflections collected	34816	
Independent reflections	6100 [$R(int)$] = 0.0539]	
Completeness to theta = 67.684°	99.8 %	
Refinement method	Full-matrix least-squares on F^2	
Data / restraints / parameters	6100 / 0 / 408	
Goodness-of-fit on F^2	1.050	
Final R indices [I>2sigma(I)]	R_1 = 0.0702, wR_2 = 0.1674	
R indices (all data)	R_1 = 0.0750, wR_2 = 0.1708	
Absolute structure parameter	0.28(13)	
Largest diff. peak and hole	0.506 and -0.392 e.$Å^{-3}$	

$R1 = \Sigma||Fo| - |Fc||/\Sigma|Fo|$. [b] $wR2 = [\Sigma w(Fo^2 - Fc^2)^2/\Sigma w(Fo^2)^2]^{1/2}$

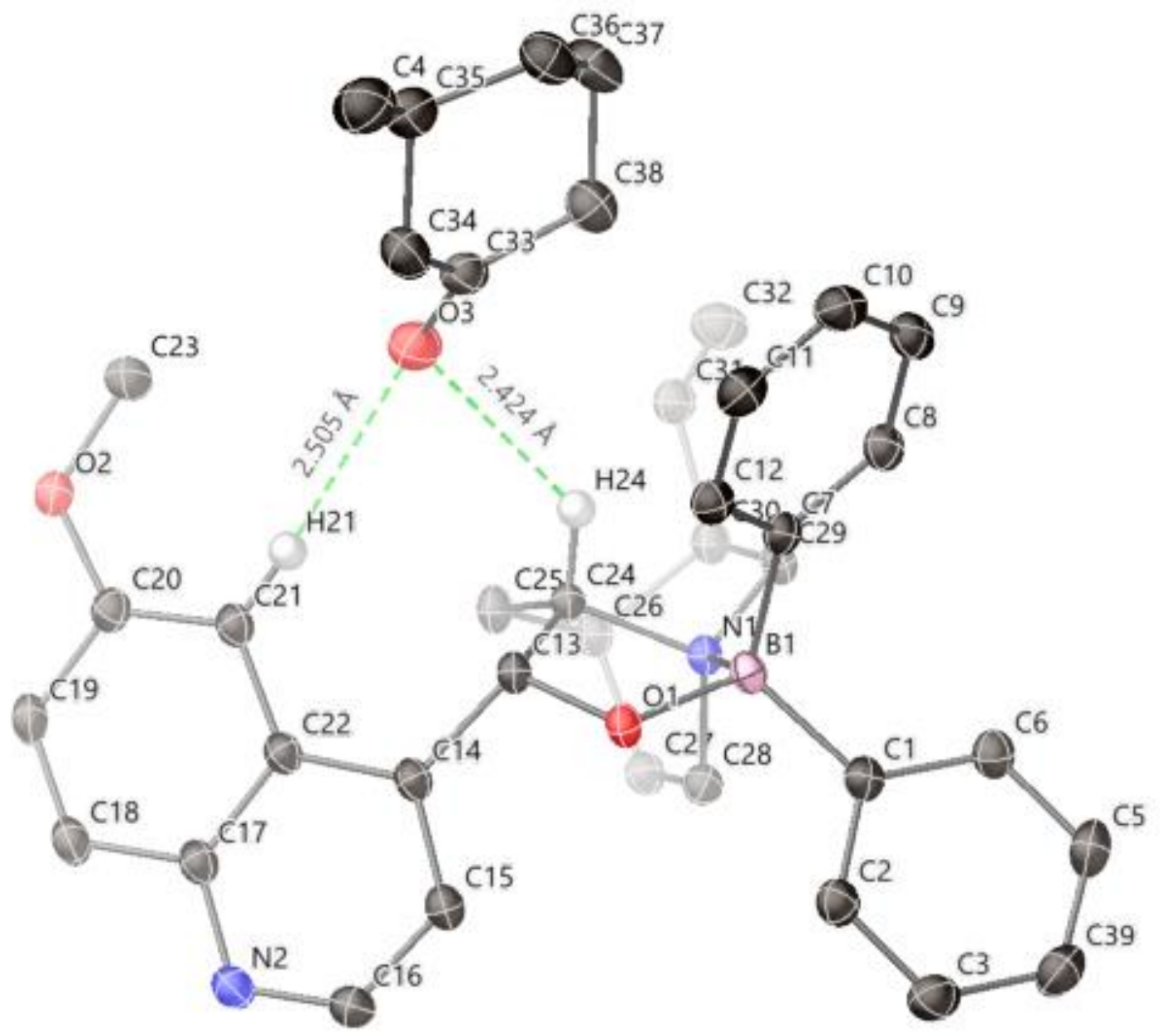

Fig. S9. The molecular structure of **4·(3-MCH)** with thermal ellipsoids set at 50% probability. Hydrogen atoms have been omitted for clarity.

Table S8. Crystallographic data and structure refinement parameters for **4 (3MCH)**

Empirical formula	$C_{39}H_{45}BN_2O_3$	
Formula weight	600.58	
Temperature	100(2) K	
Wavelength	1.54184 Å	
Crystal system	Monoclinic	
Space group	$P2_1$	
Unit cell dimensions	*a* = 10.44360(10) Å	α = 90°.
	b = 9.26440(10) Å	β = 103.1770(10)°.
	c = 17.1876(2) Å	γ = 90°.
Volume	1619.18(3) Å^3	
Z	2	
Density (calculated)	1.232 Mg/m^3	
Absorption coefficient	0.597 mm^{-1}	
F(000)	644	
Theta range for data collection	2.640 to 70.129°.	
Index ranges	-12<=*h*<=9, -11<=*k*<=11, -20<=*l*<=20	
Reflections collected	23967	
Independent reflections	6087 [*R(int)* = 0.0212]	
Completeness to theta = 67.684°	99.9 %	
Refinement method	Full-matrix least-squares on F^2	
Data / restraints / parameters	6087 / 1 / 416	
Goodness-of-fit on F^2	1.022	
Final R indices [I>2sigma(I)]	R_1 = 0.0279, wR_2 = 0.0728	
R indices (all data)	R_1 = 0.0281, wR_2 = 0.0730	
Absolute structure parameter	0.03(4)	
Largest diff. peak and hole	0.201 and -0.209 e.Å^{-3}	

R1 = Σ||Fo| − |Fc||/Σ|Fo|. [b] *$wR2 = [\Sigma w(Fo^2 - Fc^2)^2/\Sigma w(Fo^2)^2]^{1/2}$*

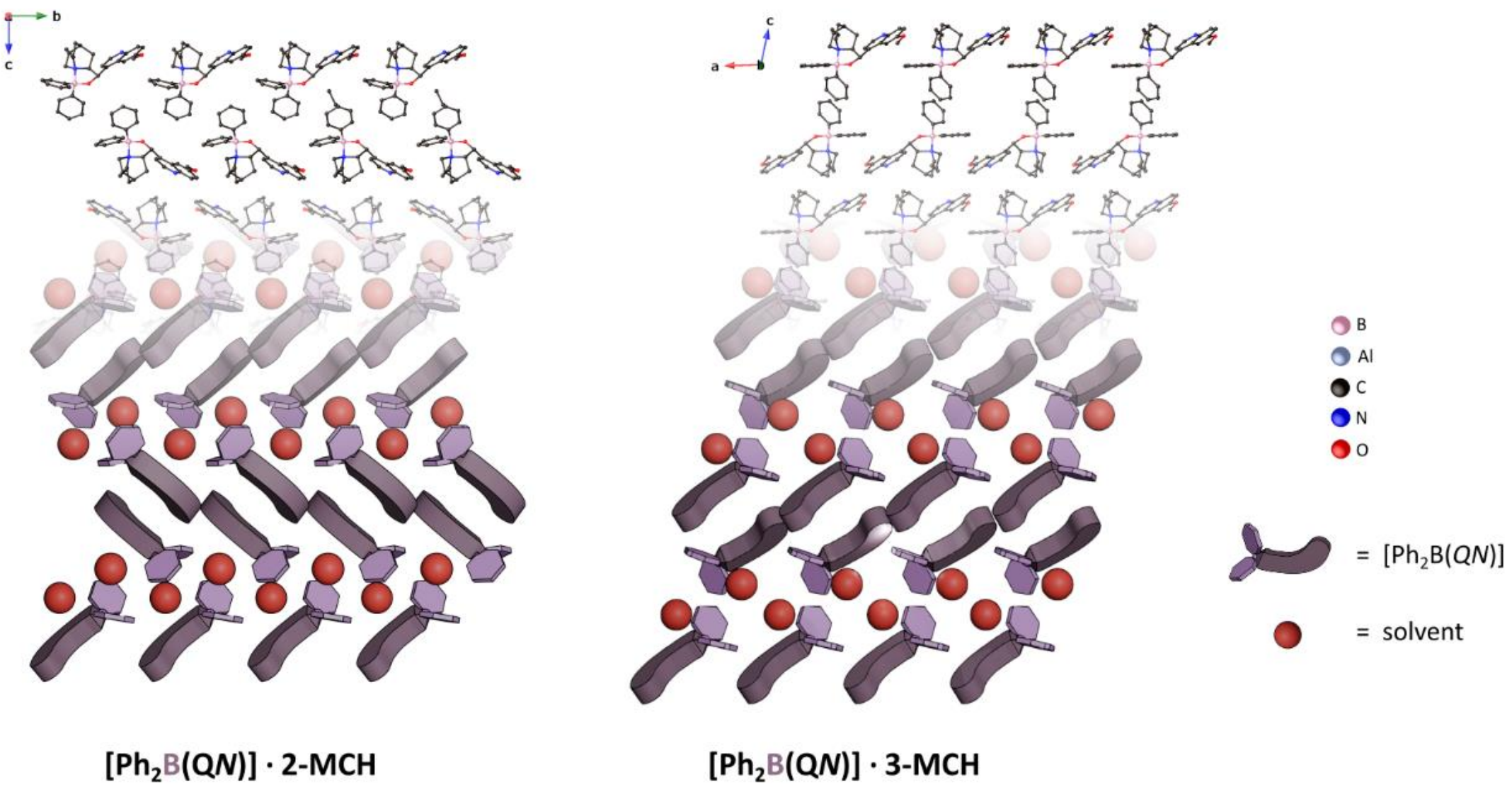

Fig. S10. Comparison of the supramolecular structures of **4**·(2-MCH) and **4**·(3-MCH).

Colorless, well-shaped crystals of **4**·MeCN were isolated from the MeCN fractions obtained in the extraction experiments by crystallization at room temperature. SC-XRD analysis showed that **4**·MeCN adopts an analogous framework to that observed for **4**·THF, **4**·MeTHF, **4**·GVL, and **4**·(2-MCH), with MeCN molecules confined within the lattice voids and oriented through weak C-H···N interactions (Fig. S11).

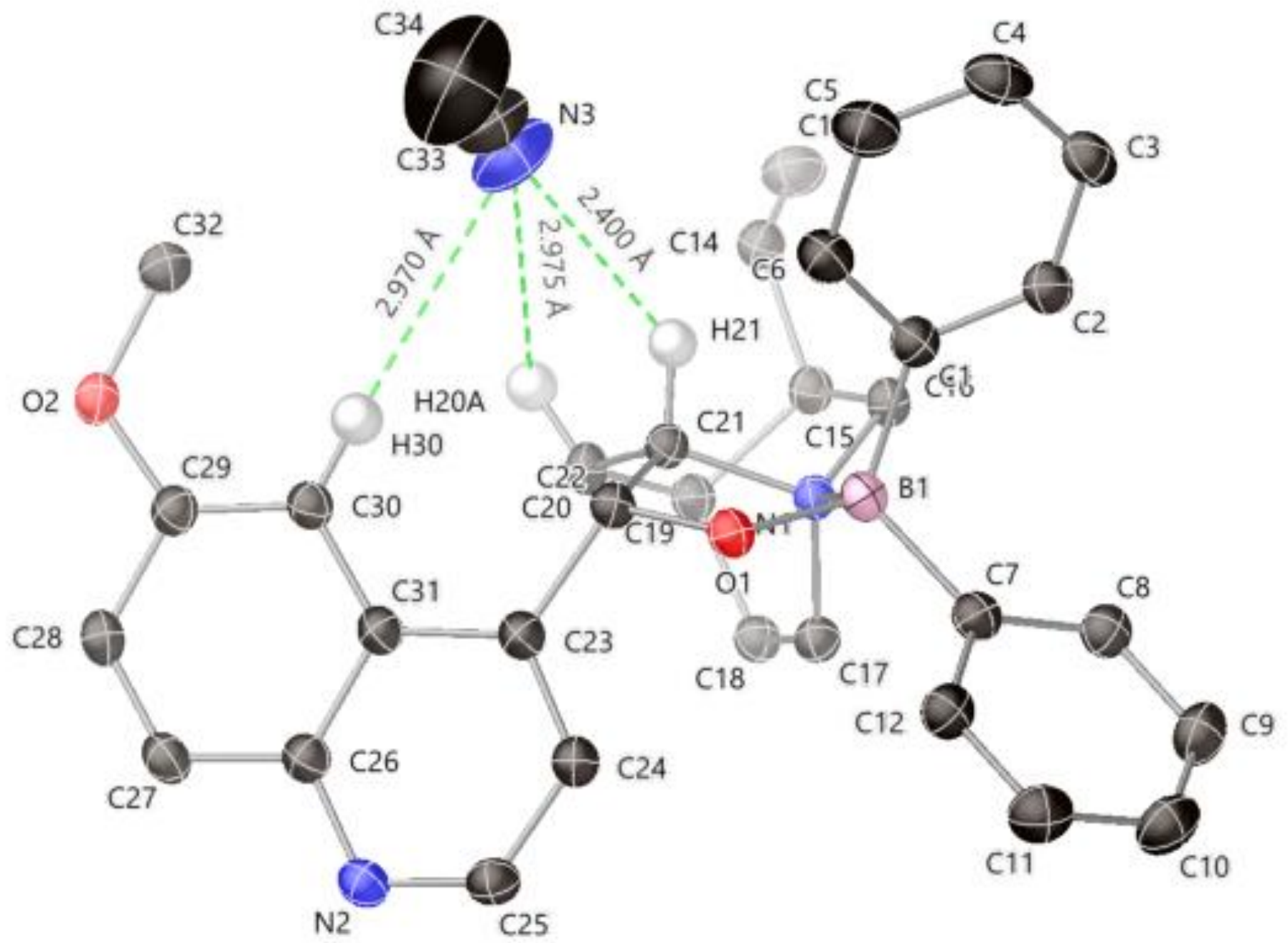

Fig. S11. The molecular structure of **4**·MeCN with thermal ellipsoids set at 50% probability. Hydrogen atoms have been omitted for clarity.

Table S9. Crystallographic data and structure refinement parameters for **4·MeCN**

Empirical formula	$C_{34}H_{36}BN_3O_2$	
Formula weight	529.47	
Temperature	100(2) K	
Wavelength	1.54184 Å	
Crystal system	Orthorhombic	
Space group	$P2_12_12_1$	
Unit cell dimensions	a = 9.13260(10) Å	α = 90°.
	b = 10.60050(10) Å	β =90°.
	c = 29.9181(2) Å	γ = 90°.
Volume	2896.38(5) $Å^3$	
Z	4	
Density (calculated)	1.214 Mg/m^3	
Absorption coefficient	0.587 mm^{-1}	
F(000)	1128	
Theta range for data collection	2.954 to 70.610 °.	
Index ranges	$-11<=h<=10$, $-12<=k<=11$, $-36<=l<=35$	
Reflections collected	13031	
Independent reflections	5511 *[R(int)* = 0.0131]	
Completeness to theta = 67.684°	99.9 %	
Refinement method	Full-matrix least-squares on F^2	
Data / restraints / parameters	5511/ 0 / 363	
Goodness-of-fit on F^2	1.030	
Final R indices [I>2sigma(I)]	R_1 = 0.0349, wR_2 = 0.0907	
R indices (all data)	R_1 = 0.0354, wR_2 = 0.0915	
Absolute structure parameter	0.05(5)	
Largest diff. peak and hole	0.226 and -0.238 e.$Å^{-3}$	

$R1 = \Sigma||Fo| - |Fc||/\Sigma|Fo|$. [b] $wR2 = [\Sigma w(Fo^2 - Fc^2)^2/\Sigma w(Fo^2)^2]^{1/2}$

Table S10. Calculated unit cell volumes (relative to the corresponding THF solvates), void volumes, van der Waals volumes of the guest molecules, and guest-to-void volume ratios for various solvates of the corresponding Al- and B-based crystalline sponges.

Structure	Unit cell volume		Voids volume[b]		Guest van der Waals volume [Å³][c]	Guest-to-void volume ratio
	total [Å³]	relative to the THF solvate[a]	fraction of the unit cell	per one guest molecule [Å³]		
[MeAl(*CN*)$_2$]·THF [d]	7147.7	100.0%	37.0%	440.5	74.2	20.8%
[MeAl(*CN*)$_2$]·MeTHF [d]	7077.7	99.0%	36.2%	427.3	91.5	21.4%
**2**·0.5THF	5264.1	100.0%	12.1%	159.7	74.2	46.4%
**2**·0.5MeTHF	5293.3	100.6%	12.8%	169.7	91.5	53.9%
**3**·THF	639.8	100.0%	20.3%	130.2	74.2	57.0%
**4**·THF	2943.1	100.0%	14.4%	106.1	74.2	69.9%
**4**·MeTHF	3036.9	103.2%	16.8%	127.2	91.5	71.9%
**4**·GVL	3043.3	103.4%	16.4%	124.9	97.6	78.2%
**4**·(2-MCH)	3227.1	109.6%	21.9%	176.3	123.4	70.0%
**4**·(3-MCH)	1619.2	110.0%	24.0%	194.3	123.4	63.5%
**4**·MeCN	2896.4	98.4%	13.3%	96.1	44.6	46.4%

[a]Relative unit cell volume (%) compared to the THF-containing analogue.
[b]Calculated as solvent-accessible voids using a probe radius of 1.41 Å and a grid spacing of 0.3 Å.
[c]Van der Waals volume of the related guest molecule calculated according to reference [6]
[d]Calculated using previously reported structures.[7]

3. NMR Spectra

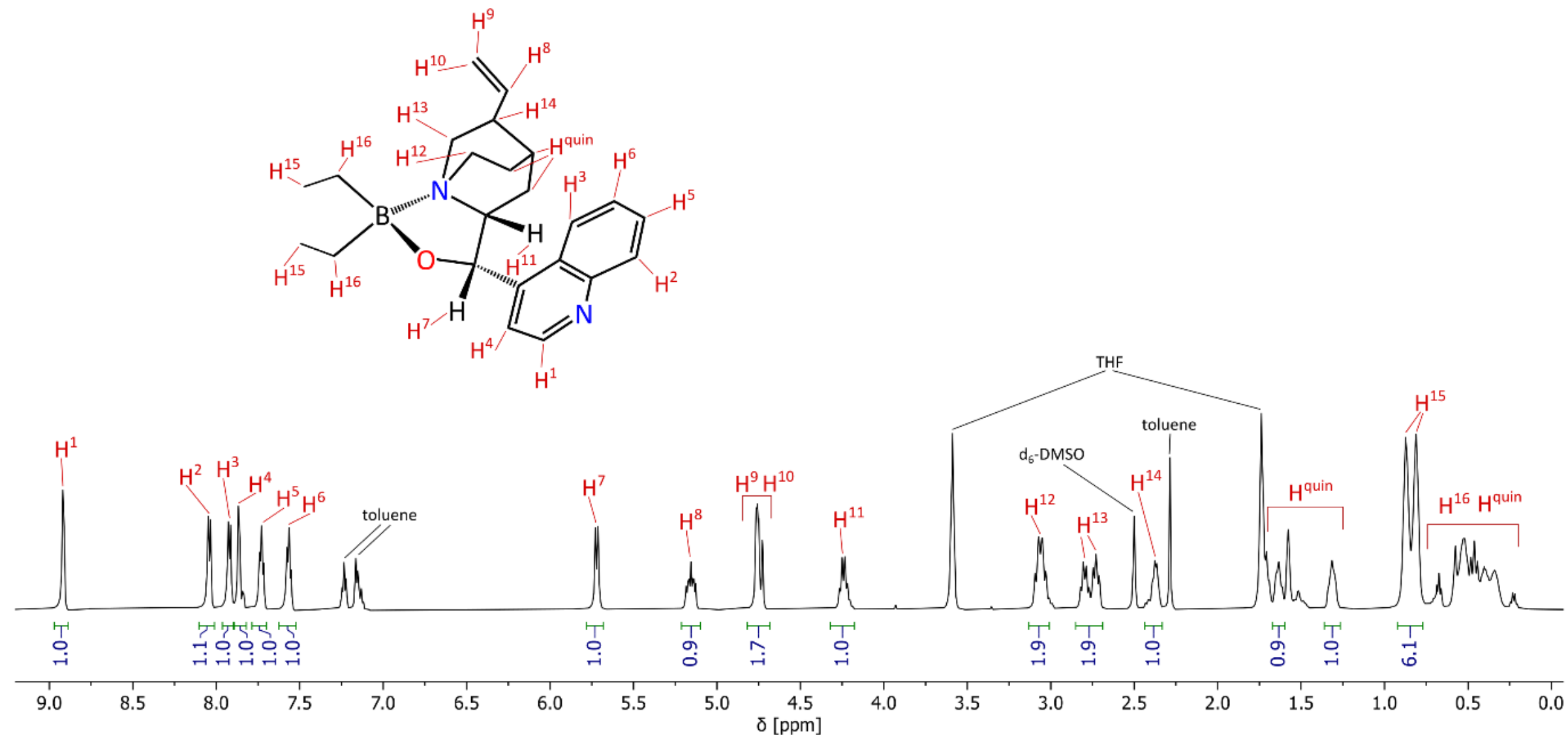

Fig. S12. ^{1}H NMR spectrum of **1** in d_6-DMSO.

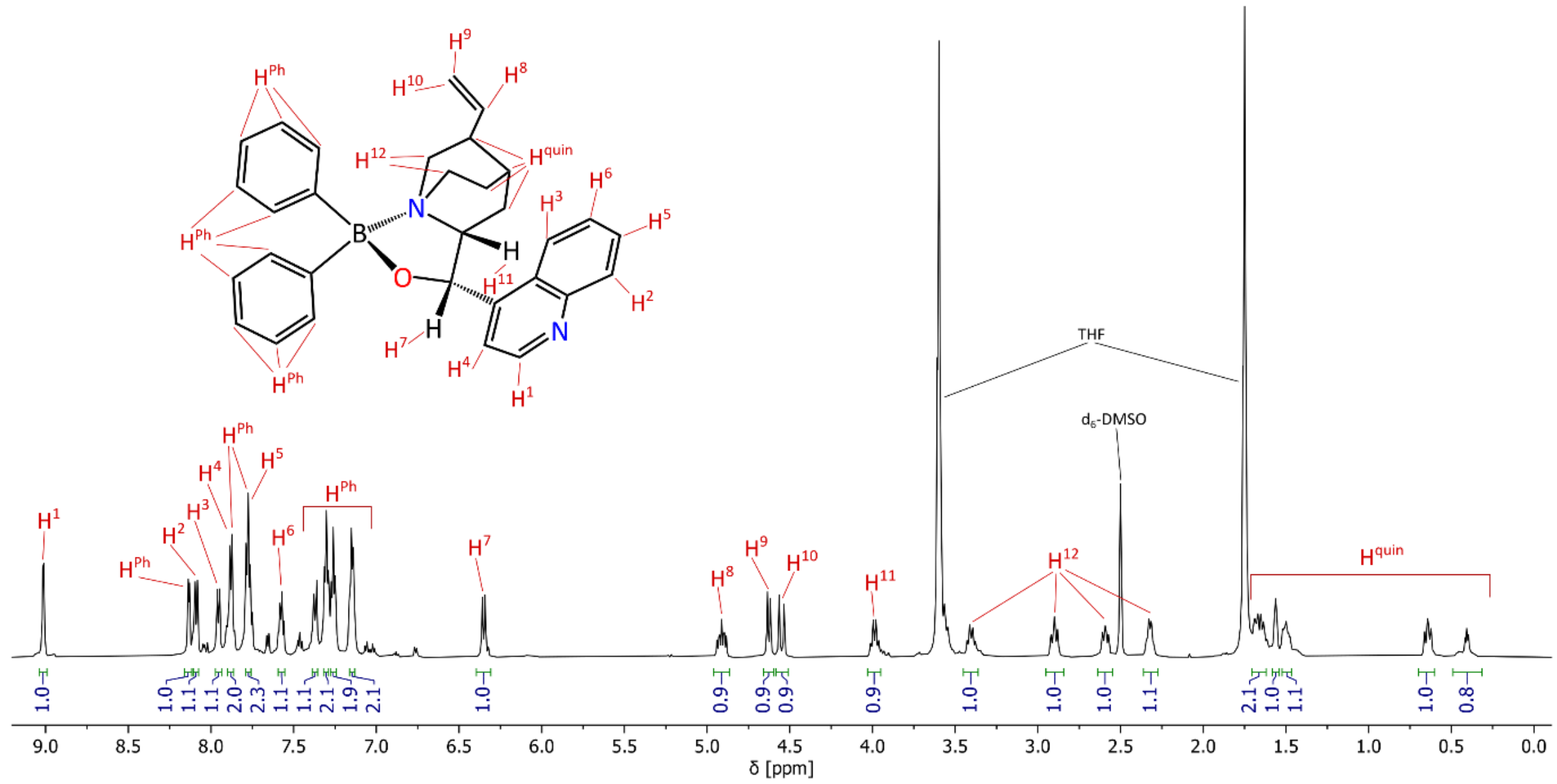

Fig. S13. ^{1}H NMR spectrum of **2** in d_6-DMSO.

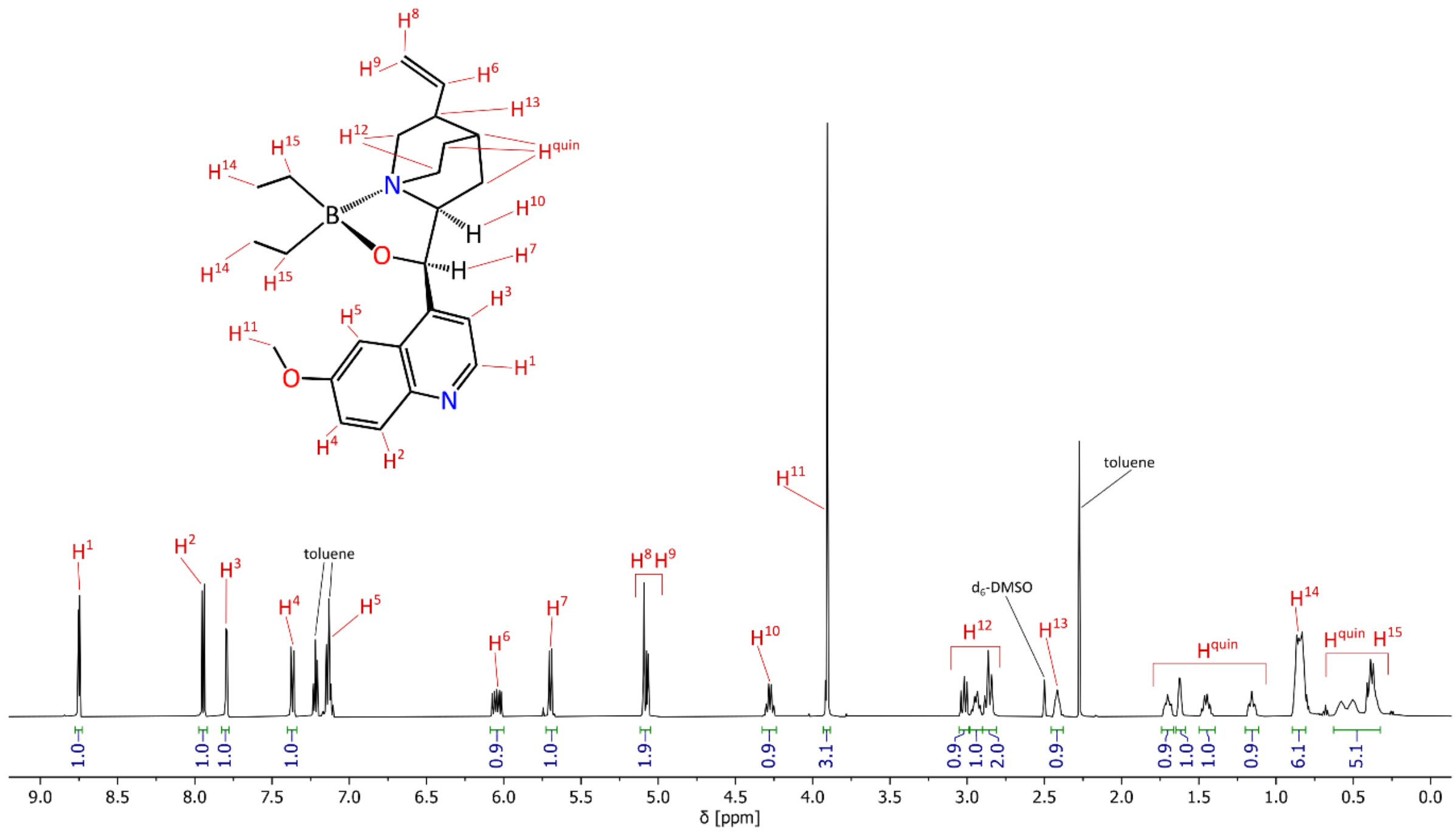

Fig. S14. ^{1}H NMR spectrum of **3** in d_6-DMSO.

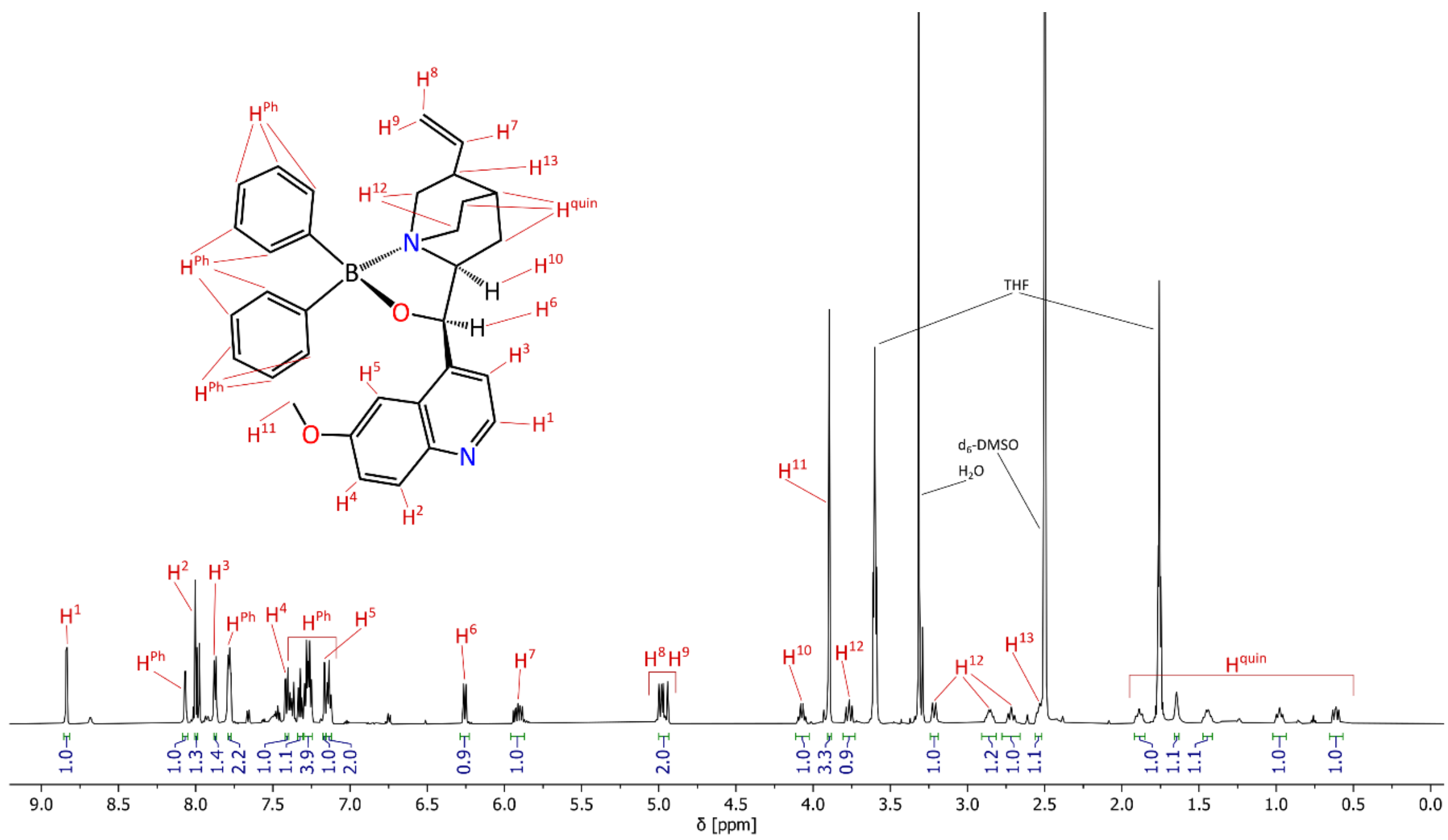

Fig. S15. ^{1}H NMR spectrum of **4** in d_6-DMSO.

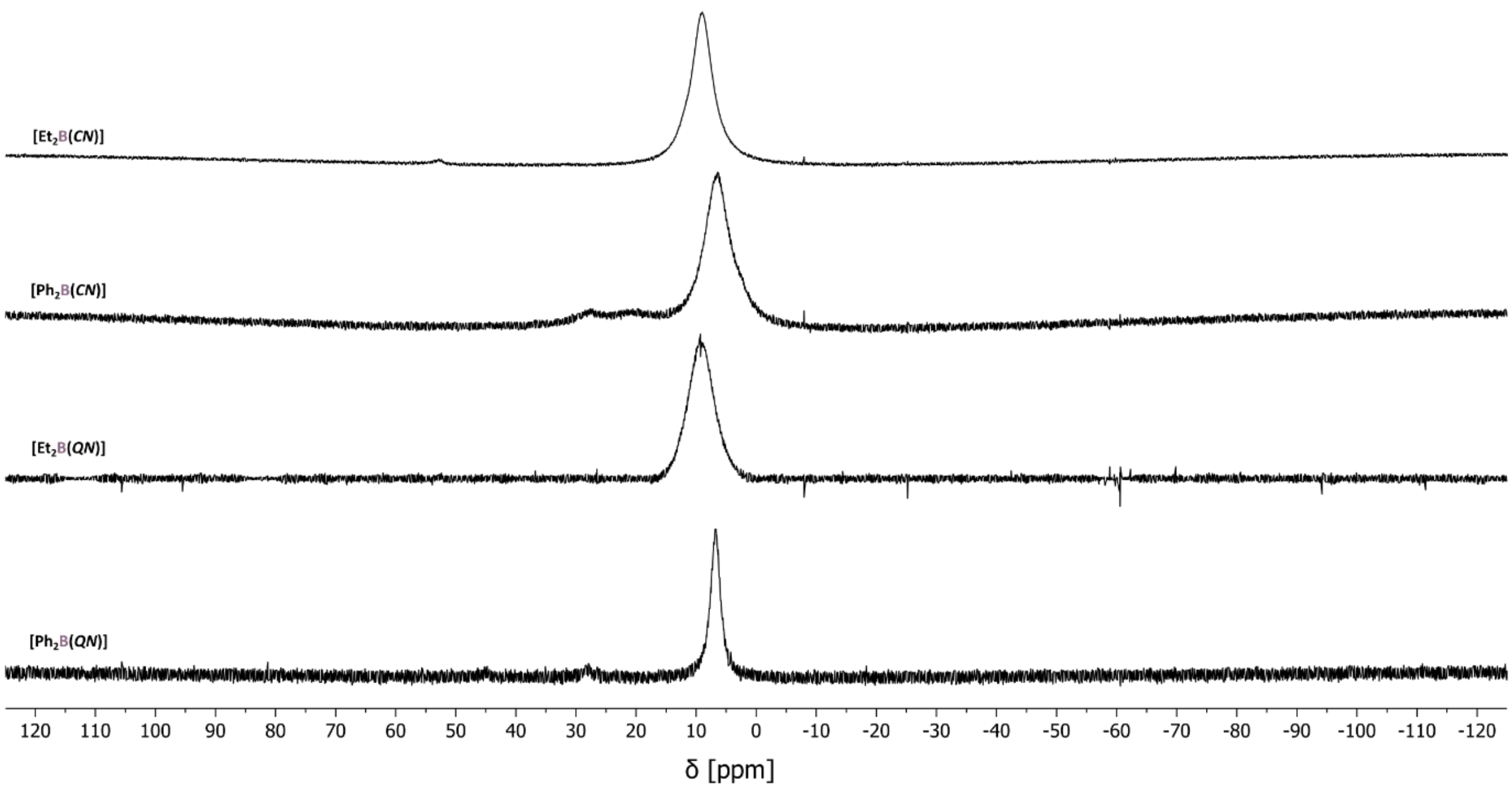

Fig. S16. ^{11}B NMR spectra of complexes **1-4** in d_6-DMSO.

4. TGA

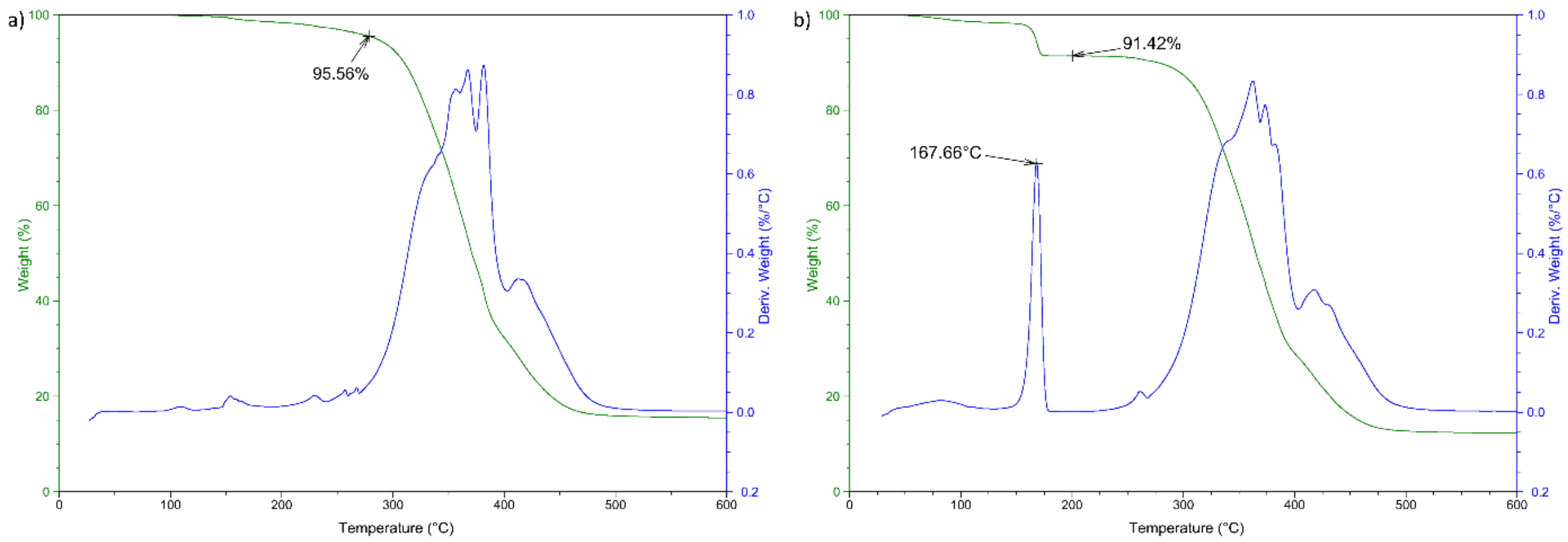

Fig. S17. TGA and DTG profiles of **2**·0.5THF (a) and **2**·0.5MeTHF (b).

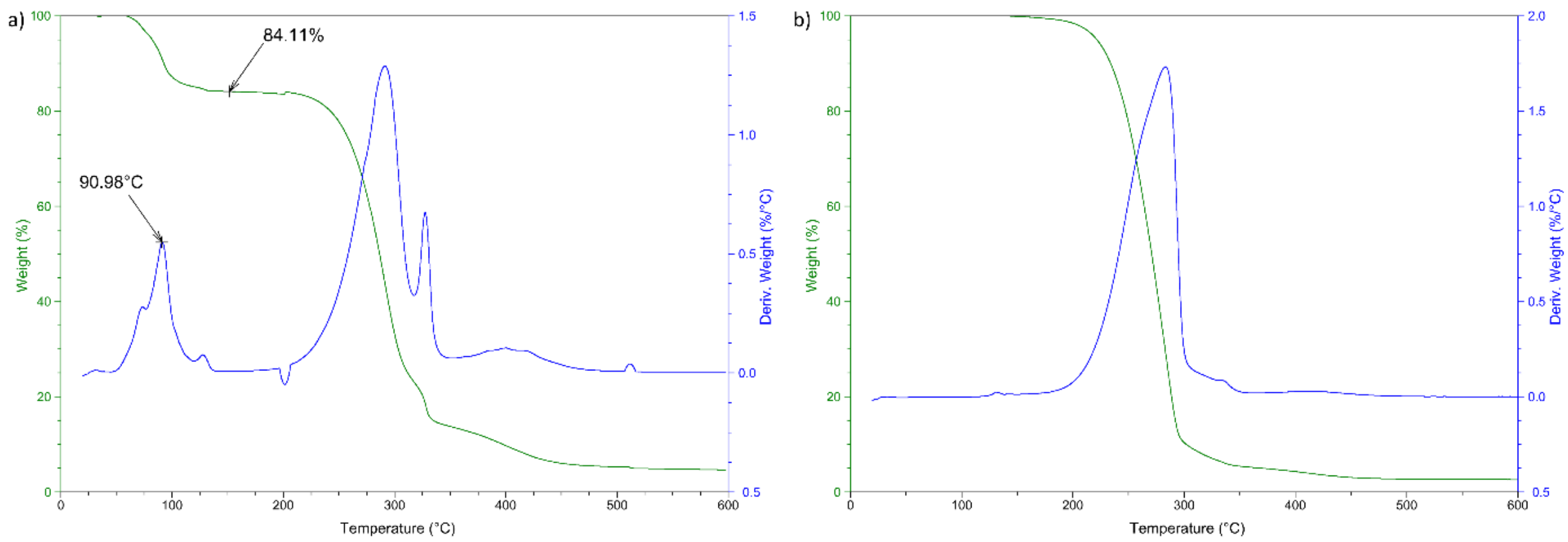

Fig. S18. TGA and DTG profiles of **3**·THF (a) and **3**·THF after recrystallization from MeTHF (b).

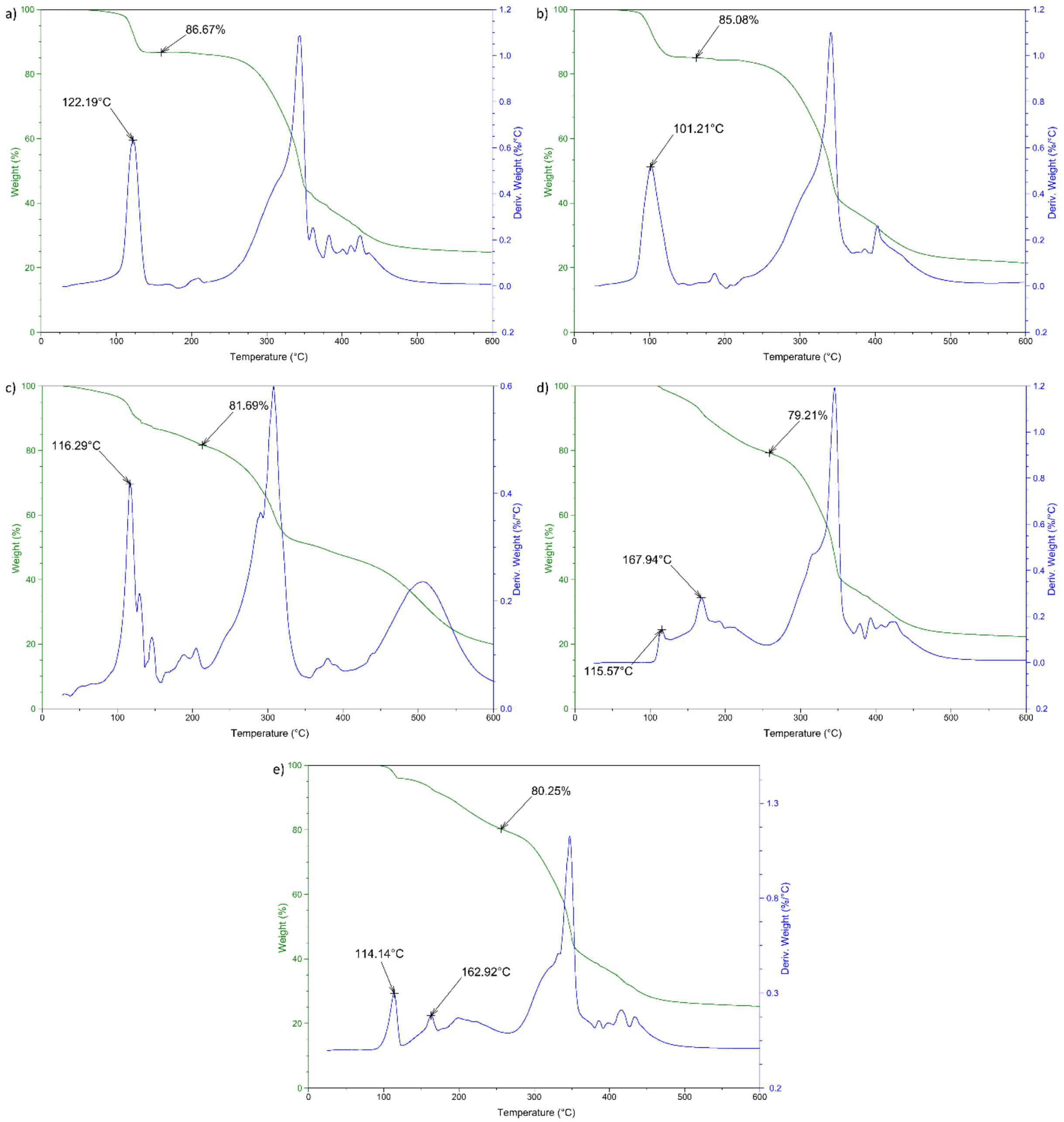

Fig. S19. TGA and DTG profiles of **4**·THF (a), **4**·MeTHF (b), **4**·GVL (c), **4**·(2-MCH) (d), and **4**·(3-MCH) (e).

Table S11. Comparison of the guest mass fraction calculated from the crystal structure and obtained from TGA analysis.

Structure	Guest mass fraction (%)	
	from the crystal structure	from TGA analysis
**2**·0.5THF	7.3	4.4
**2**·0.5MeTHF	8.6	8.6
**3**·THF	15.5	15.9
**4**·THF	12.9	13.3
**4**·MeTHF	15.0	14.9
**4**·GVL	17.0	18.3
**4**·(2-MCH)	18.9	20.8
**4**·(3-MCH)	18.9	19.8

5. PXRD

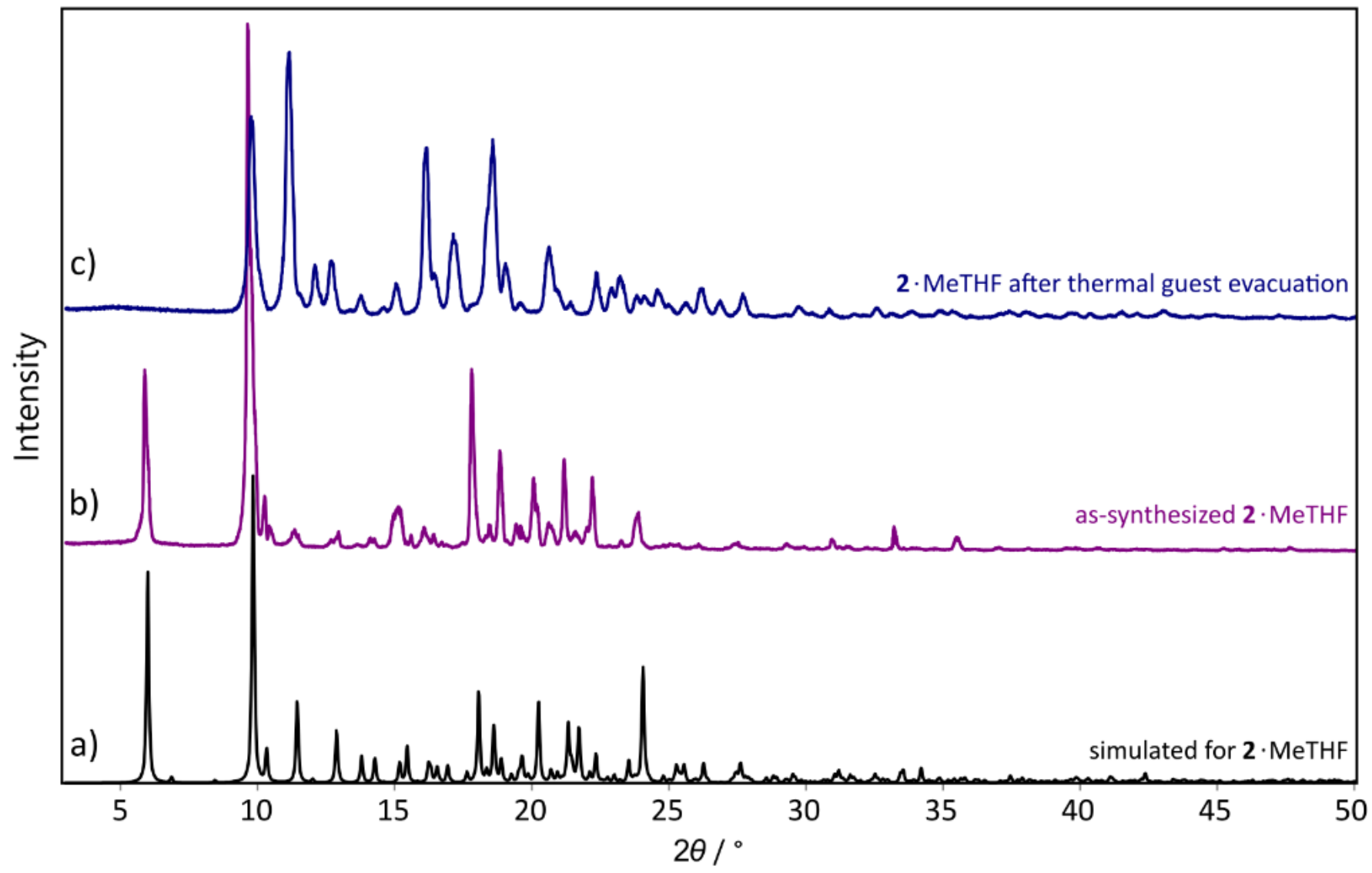

Fig. S20. Powder patterns of **2**·0.5MeTHF: a) simulated from the crystal structure, b) as-synthesized material, c) after thermal evacuation of guest molecules.

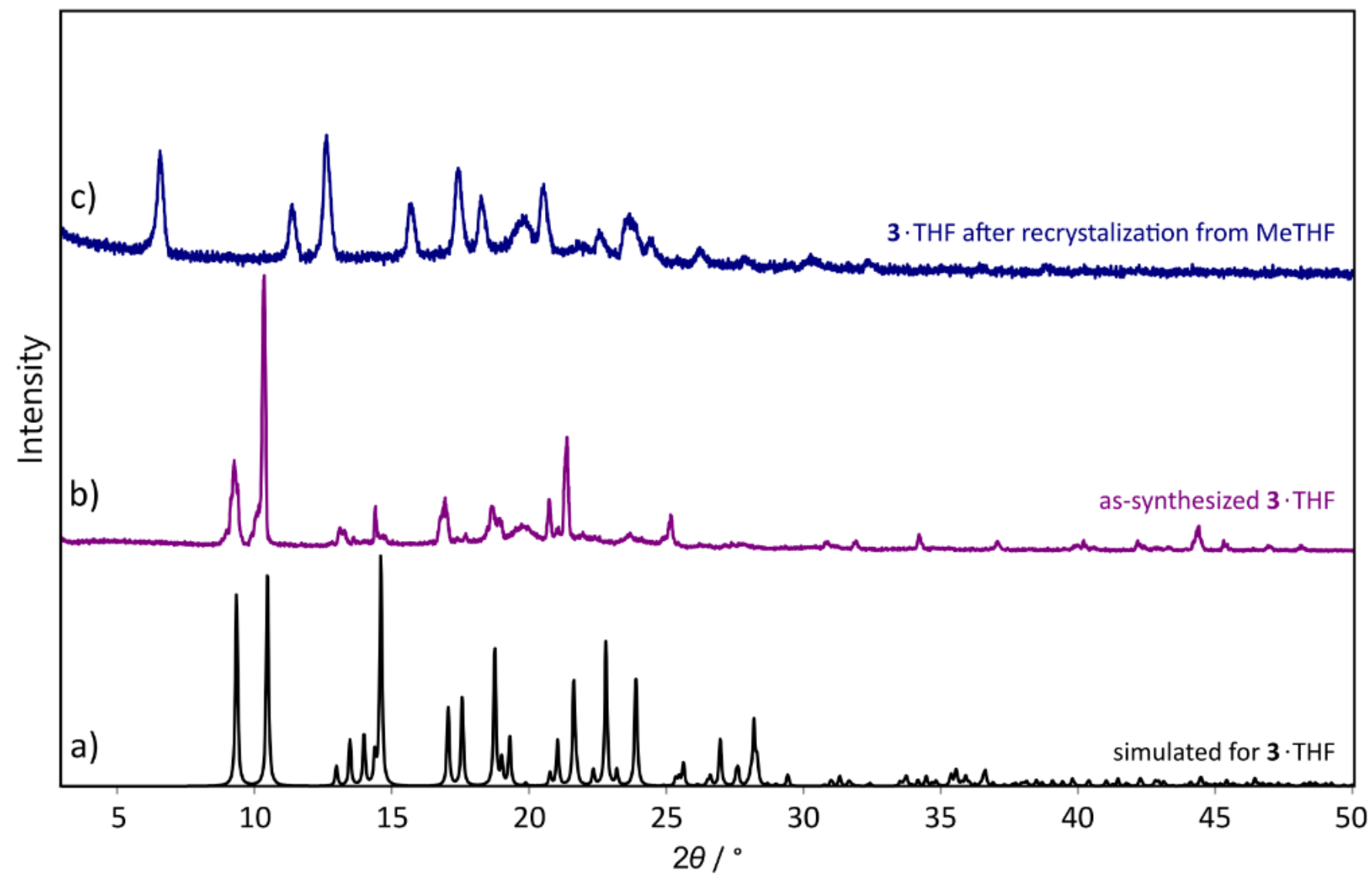

Fig. S21. Powder patterns of **3**·THF: a) simulated from the crystal structure, b) as-synthesized material, c) after recrystallization from MeTHF.

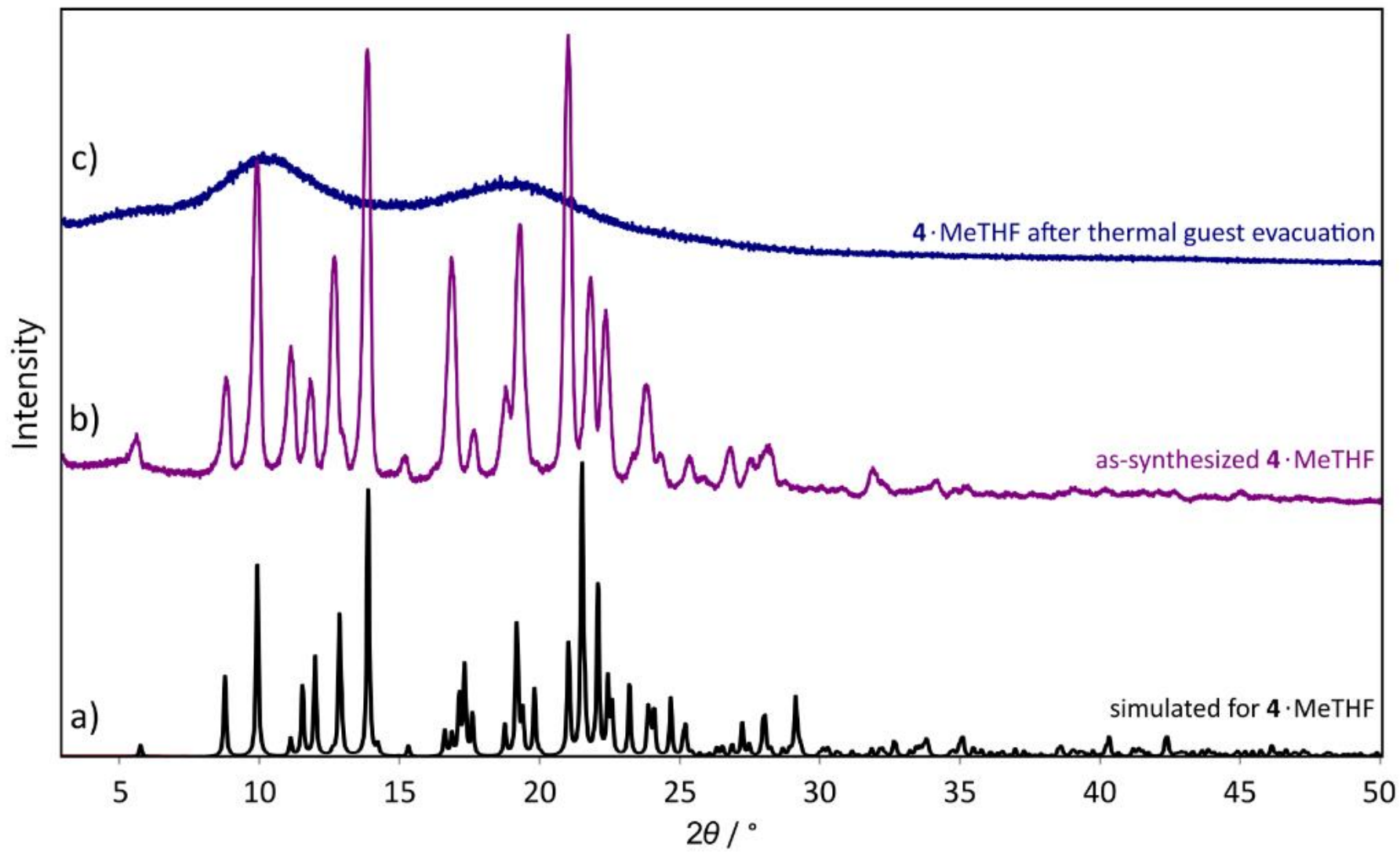

Fig. S22. Powder patterns of **4**·MeTHF: a) simulated from the crystal structure, b) as-synthesized material, c) after thermal evacuation of guest molecules.

6. Gas Chromatography

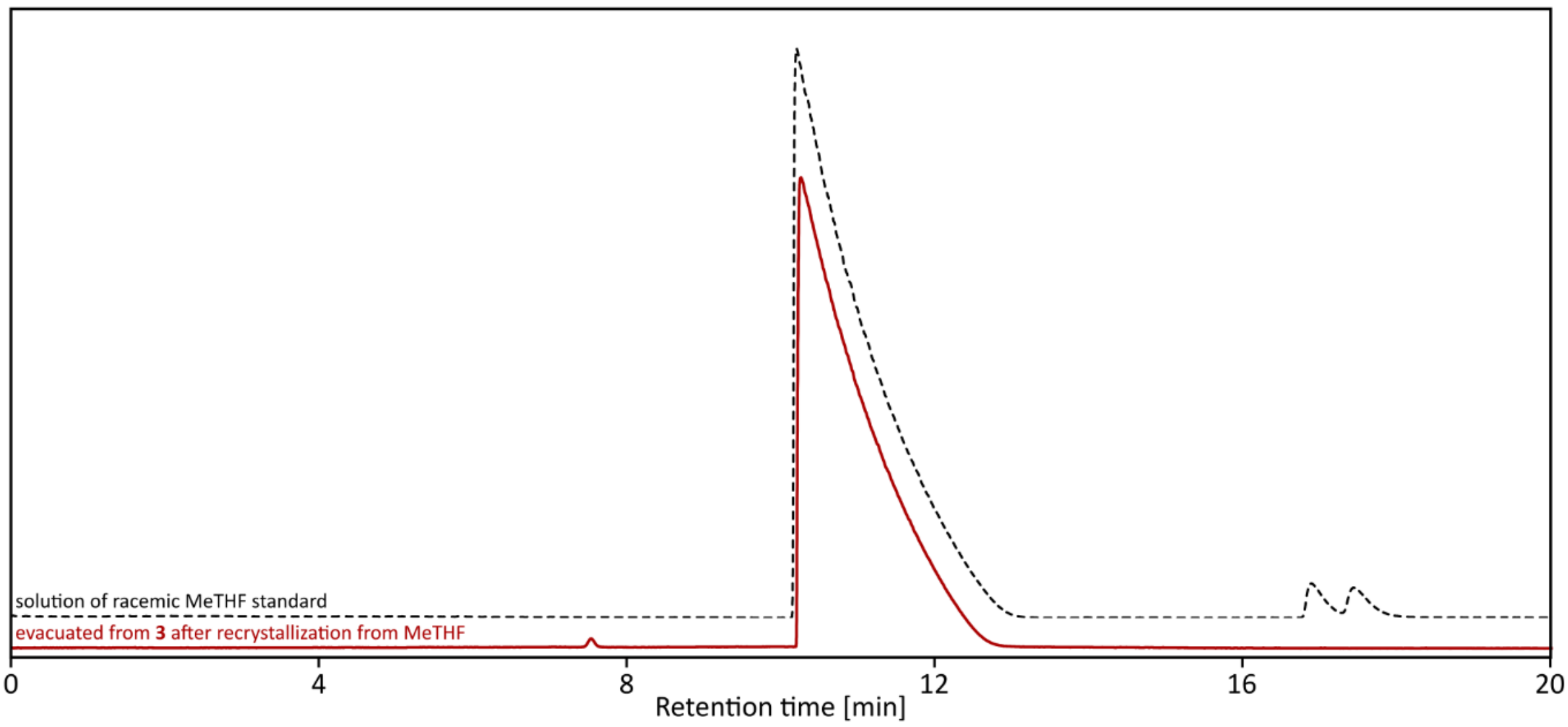

Fig. S23. Comparison of GC chromatograms of a racemic MeTHF standard and a sample released from complex **3** recrystallized from MeTHF.

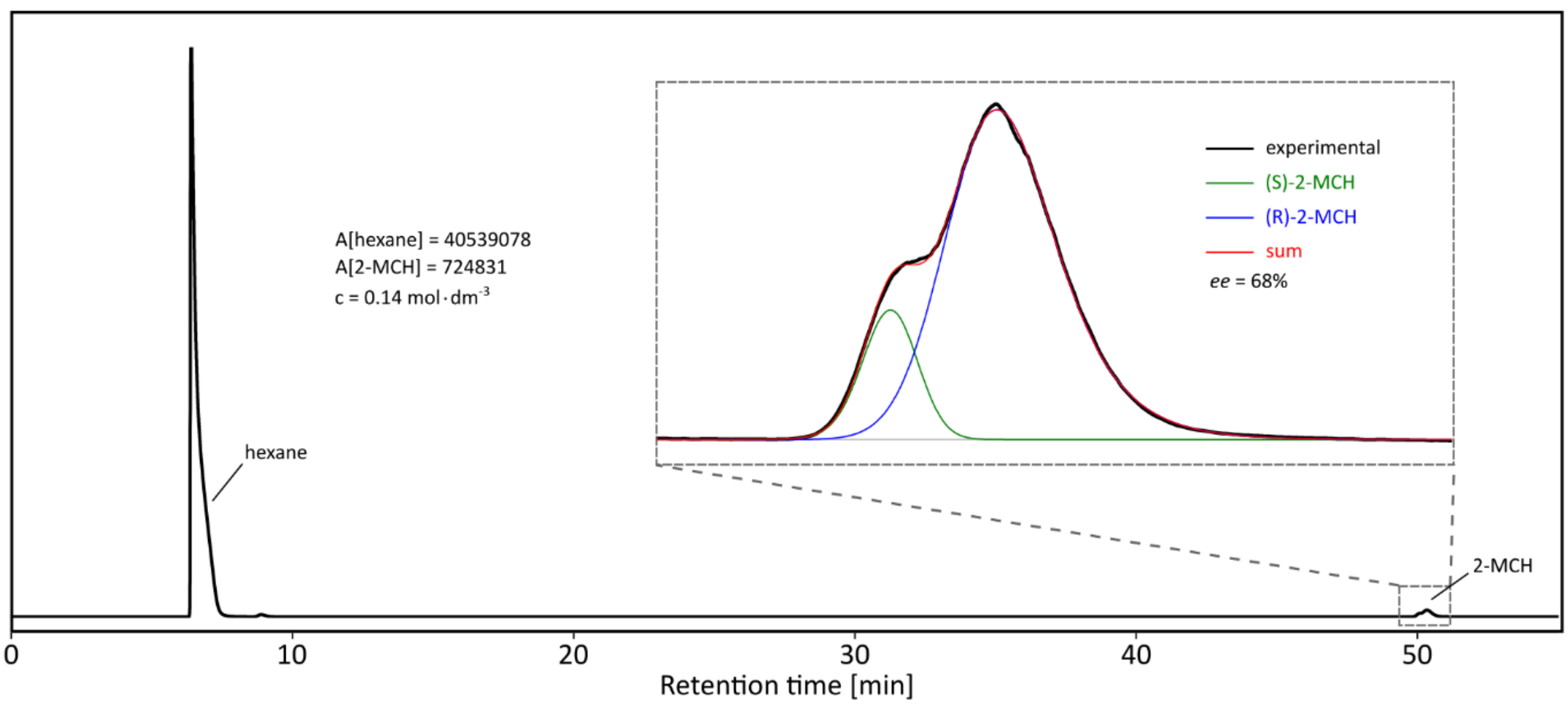

Fig. S24. Analysis of the GC chromatogram from the 2-MCH/hexane recovered from **4**·(2-MCH) *via* the extraction method.

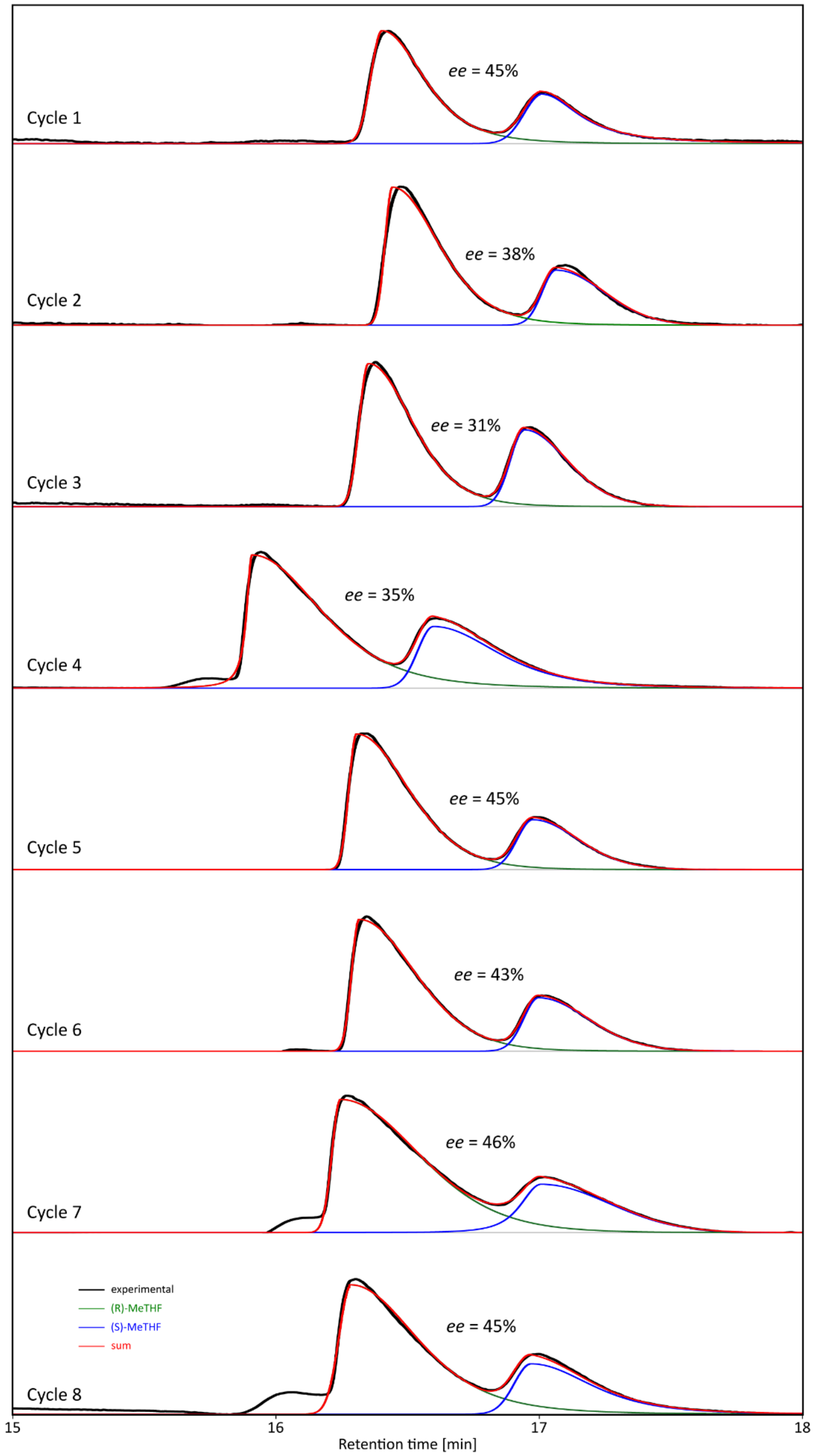

Fig. S25. Analysis of the GC chromatograms of enantioenriched MeTHF/hexane solutions received during the reproducibility experiment.

7. Hirshfeld surface analysis and interaction energy calculations

Methods.
Analysis of guest binding energies and guest positioning was performed for representative structures: **2**·0.5MeTHF, **4**·MeTHF, and **4**·(3-MCH). The molecular geometries of the guest molecules, namely (R)- and (S)-MeTHF and (R)- and (S)-3-MCH, were optimized using the BP86 density functional with the def2-SVP basis set as implemented in the ORCA software package. The optimized guest molecules were subsequently used to construct two types of host-guest models based on the experimental crystal structures (Model A) and molecular docking calculations (Model B).

Model A. The optimized guest molecules were fitted to the electron-density maxima of the experimental crystal structures.

Model B. To investigate possible alternative guest orientations within the host-generated voids, molecular docking simulations were performed using the CCDC GOLD software.[8] Instead of a protein receptor, a molecular cluster consisting of 6 host molecules extracted from the experimentally determined crystal structures was used to reproduce the local crystal environment surrounding the guest molecule. The binding site was defined as a spherical region with a radius of 6 Å centered at the geometrical center of the guest molecule determined from the experimental crystal structure. Docking calculations were performed for both (R)- and (S)-enantiomers of each guest molecule treated as ligands. The genetic algorithm (GA) settings were as follows: 100 GA runs with a search efficiency of 200%. The GoldScore function was used as the scoring function. The docking poses with the highest GoldScore values were selected for further analysis.

Hirshfeld surface and interaction energy calculations.
Analysis of close contacts and host–guest interaction energies were performed using the CrystalExplorer software (version 21.5)[9] for models A and B. Wavefunctions and interaction energies were calculated using the Tonto module implemented in CrystalExplorer at the B3LYP/6-31G(d,p) level of theory.[10] The interaction energies between molecular pairs are decomposed into four main components: electrostatic, polarization, dispersion, and exchange-repulsion terms.

For Hirshfeld surface (HS) analysis, the distances (d_e) and (d_i) are defined as the distances from the HS to the nearest nucleus outside and inside the surface, respectively. The normalized contact distance, d_{norm}, is a normalized function of d_e and d_i relative to the corresponding van der Waals radii of the atoms. It is used to identify intermolecular contacts that are shorter or longer than the sum of the corresponding van der Waals radii. The color scale used for HS mapped over d_e, d_i, and d_{norm} represents short (red), intermediate (green), and long (blue) intermolecular distances. The electrostatic potential (ESP) was mapped onto the Hirshfeld surfaces using a color scale ranging from −0.05 au (red) through 0 au (white) to +0.05 au (blue), where 1 au corresponds to 2625.5 kJ·mol^{-1} per unit charge.

Results for 4·MeTHF.

For **4**·MeTHF, Model A^R best reflects the experimental crystal structure, with the (R)-enantiomer of guest included in the model. HS analysis of the guest fragment shows that the guest molecule is in contact mainly with eight neighboring molecules: six host molecules and two other guest molecules (Fig. S26a). These molecules were therefore included in the interaction energy calculations. The largest contribution to the HS area (45.5%) corresponds to contacts with a host molecule involved in O-H···O interactions. Together with two additional host molecules, constitute the main environment of the chiral void, accounting for 89.9% of the total HS area. The mean d_i, and d_{norm} values are 1.492 and 0.478

Å, respectively, while the HS volume is 126.67 $Å^3$, indicating relatively tight packing of the guest molecule within the host framework.

The intermolecular contacts are dominated by H···H and H···C/C···H contacts, which account for 79.1% and 14.0% of the HS, respectively. O···H contacts account for an additional 6.6% of the surface and reflect weak hydrogen-bonding interactions between the MeTHF guest and the main host molecule (Fig. S26b,c). This distribution indicates that guest-host interactions are dominated by van der Waals forces, which is consistent with the interaction energy calculations showing that the dispersion component provides the largest contribution to the total interaction energy (Table S13). Furthermore, the orientation of the MeTHF molecule appears to be governed by electrostatic complementarity. The region of negative ESP around the oxygen atom of MeTHF is directed toward the region of positive ESP within the cavity of the main host molecule (Figure 4 and S27), corresponding to the C-H···O interactions. This arrangement results in an significant contribution from the electrostatic energy component to the total interaction energy for this host–guest pair (Table S13).

Interestingly, placing the (S)-enantiomer of MeTHF in similar position (Model A^S) results in a significant increase in repulsion energy, associated with increased steric hindrance, while maintaining the overall binding energy at a similar level (Table S12). This difference in the individual energy components results in a more favorable overall interaction energy for the (R)-enantiomer by approximately 12.4 kJ mol^{-1}, consistent with the enantioseparation experiments.

To explore alternative guest positions in the **4**·MeTHF system, docking calculations were performed using the CCDC GOLD software, which revealed marked differences in the behavior of the R and S enantiomers. While (R)-MeTHF preferentially samples position close to that observed in the crystal structure (RMS between Model A^R and B^R is 0.226), for (S)-MeTHF a broader range of orientations was found, which differ significantly from guest position observed in the crystal structure (Fig. S28). The optimal GoldScore was slightly higher for (R)-MeTHF (33.0644) than for (S)-MeTHF (32.7102), in agreement with the experimentally observed enantiodiscrimination. In the optimal Model B^S, (S)-MeTHF preserves a similar overall shape and methyl-group position as (R)-enantiomer but reverses the orientation of the ether oxygen atom (Fig. S27). This orientation decreases steric repulsion, but at the cost of disrupting electrostatic complementarity and the favorable O···H contacts, which results in lowering the electrostatic binding energy (Table S14). Therefore, total host–guest interaction energy in model BS remains less favorable than for the preferred (R)-guest models. Thus, the enantioselective inclusion of MeTHF is likely governed by the combined complementarity of molecular shape and ESP, which together establishes an effective lock-and-key recognition mechanism.

Notably, although CCDC GOLD can be useful for analyzing host–guest frameworks in molecular crystals,[11] the above results highlight some limitations in its ability to describe subtle, directional interactions. In the present system, GOLD efficiently identifies the region of the cavity where the MeTHF molecule best fits and fills the available space, but it does not necessarily reproduce the precise orientation required to optimize dipole alignment and weak C-H···O interactions, as revealed by the complementary interaction-energy analysis. Thus, GOLD provides a useful description of the overall geometrical fit, while detailed quantum-chemical analysis is required to capture the subtle energetic balance governing the preferred host–guest arrangement.

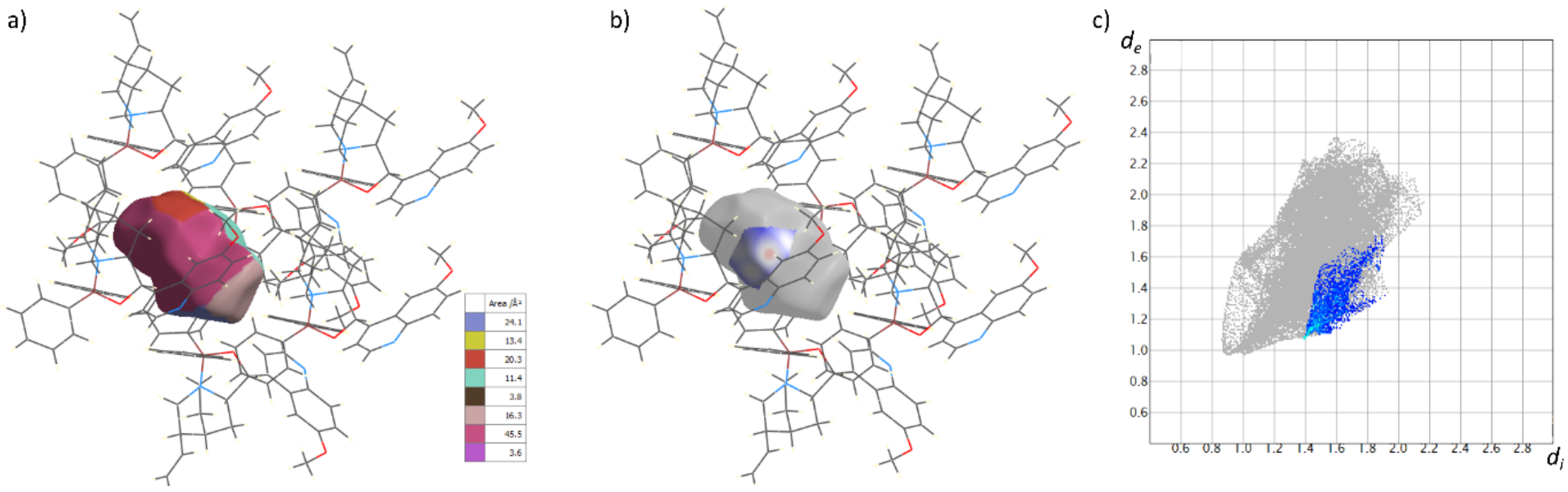

Fig. S26. Hirshfeld surface analysis of the guest molecule in Model A^R of the **4**·MeTHF system: (a) fragment patch map; (b) d_{norm} map highlighting the CH···O contacts; (c) fingerprint plot of d_e/d_i distances highlighting the CH···O contacts.

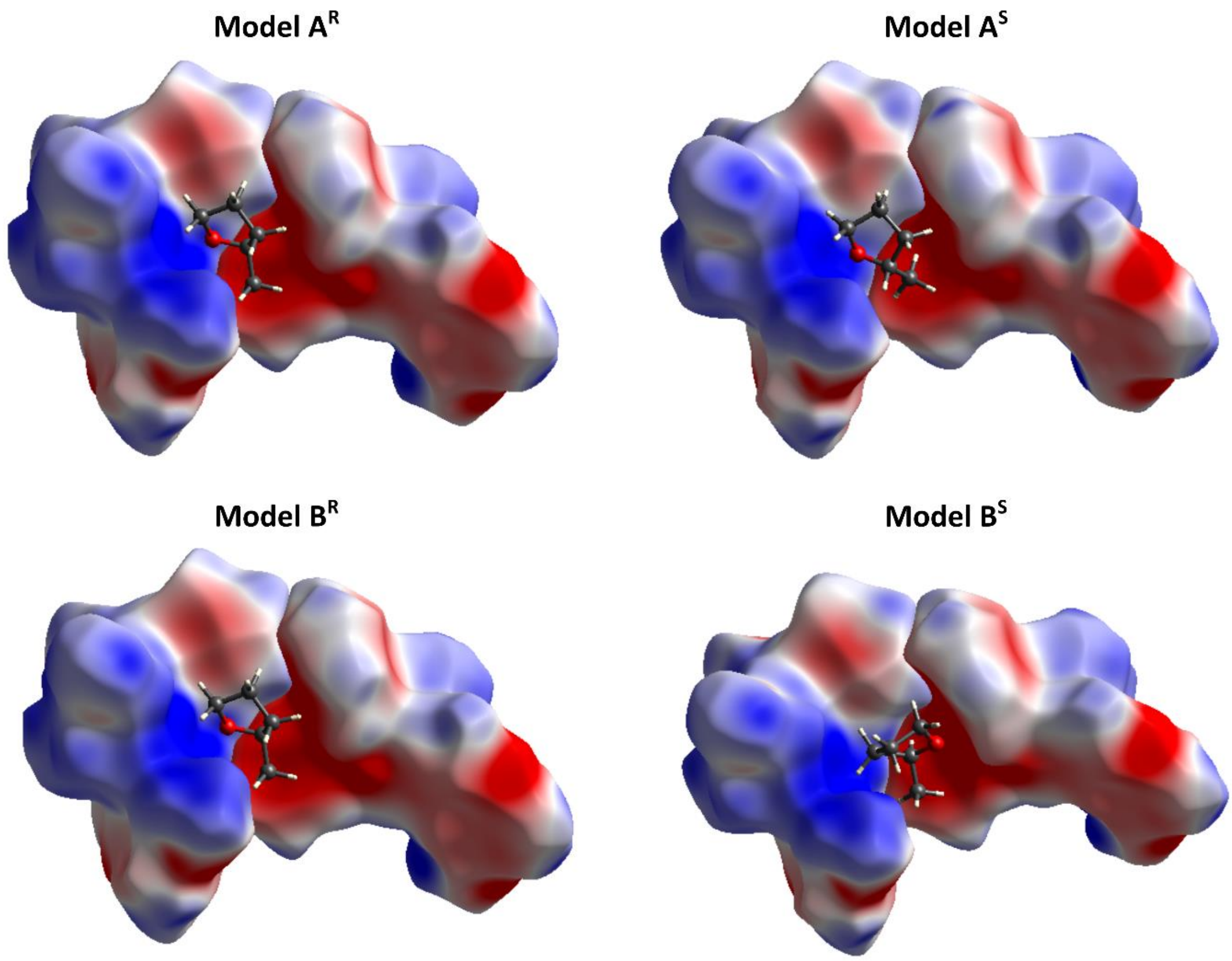

Fig. S27. Positions of the guest molecules within the voids in Models A and B of the **4**·MeTHF system. The Hirshfeld surfaces of the host molecules are mapped with the ESP over the range from -0.05 au (red) to +0.05 au (blue).

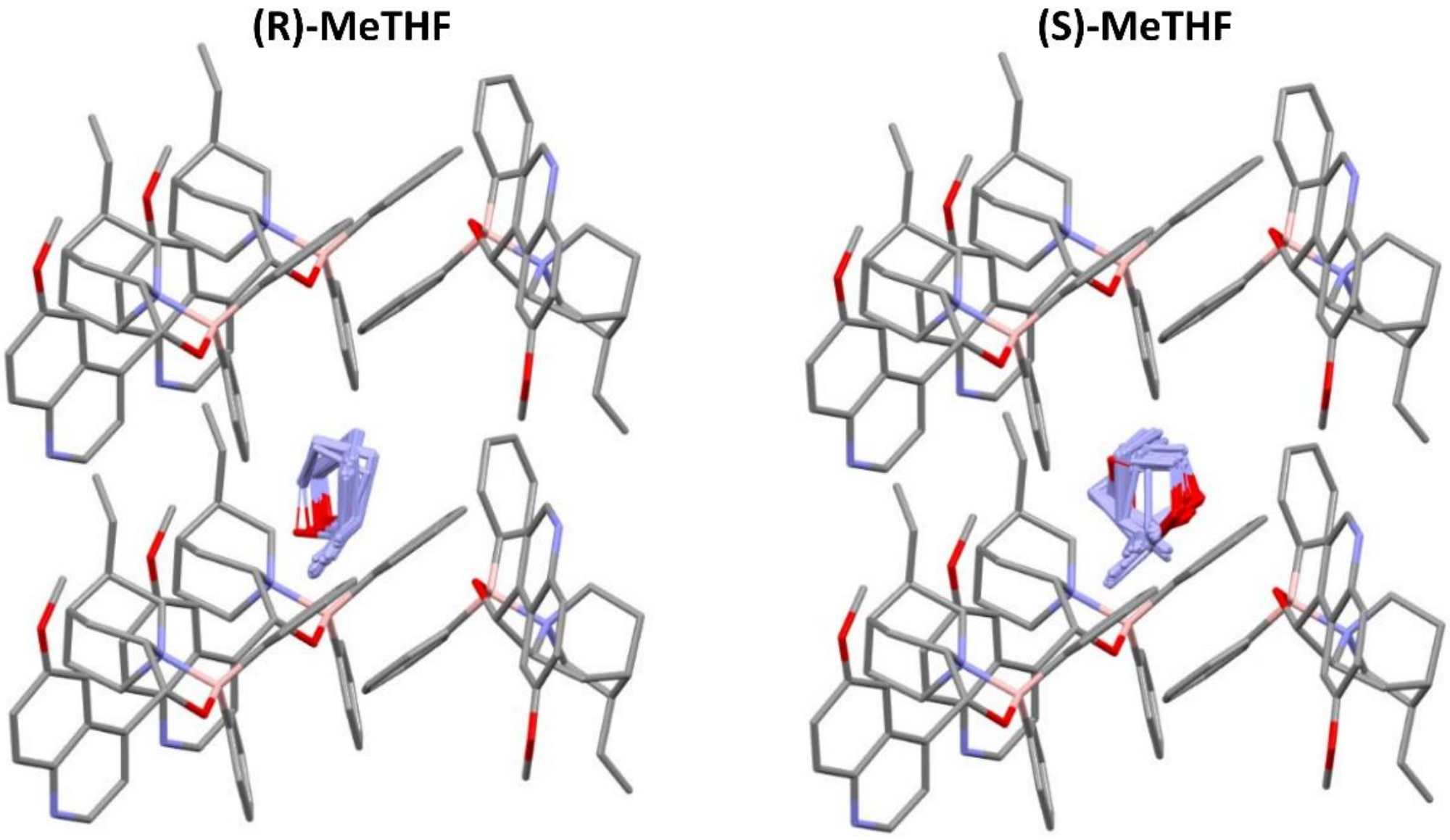

Fig. S28. Simulated poses of (R)- and (S)-MeTHF within the voids of **4**·MeTHF, obtained from molecular docking calculations.

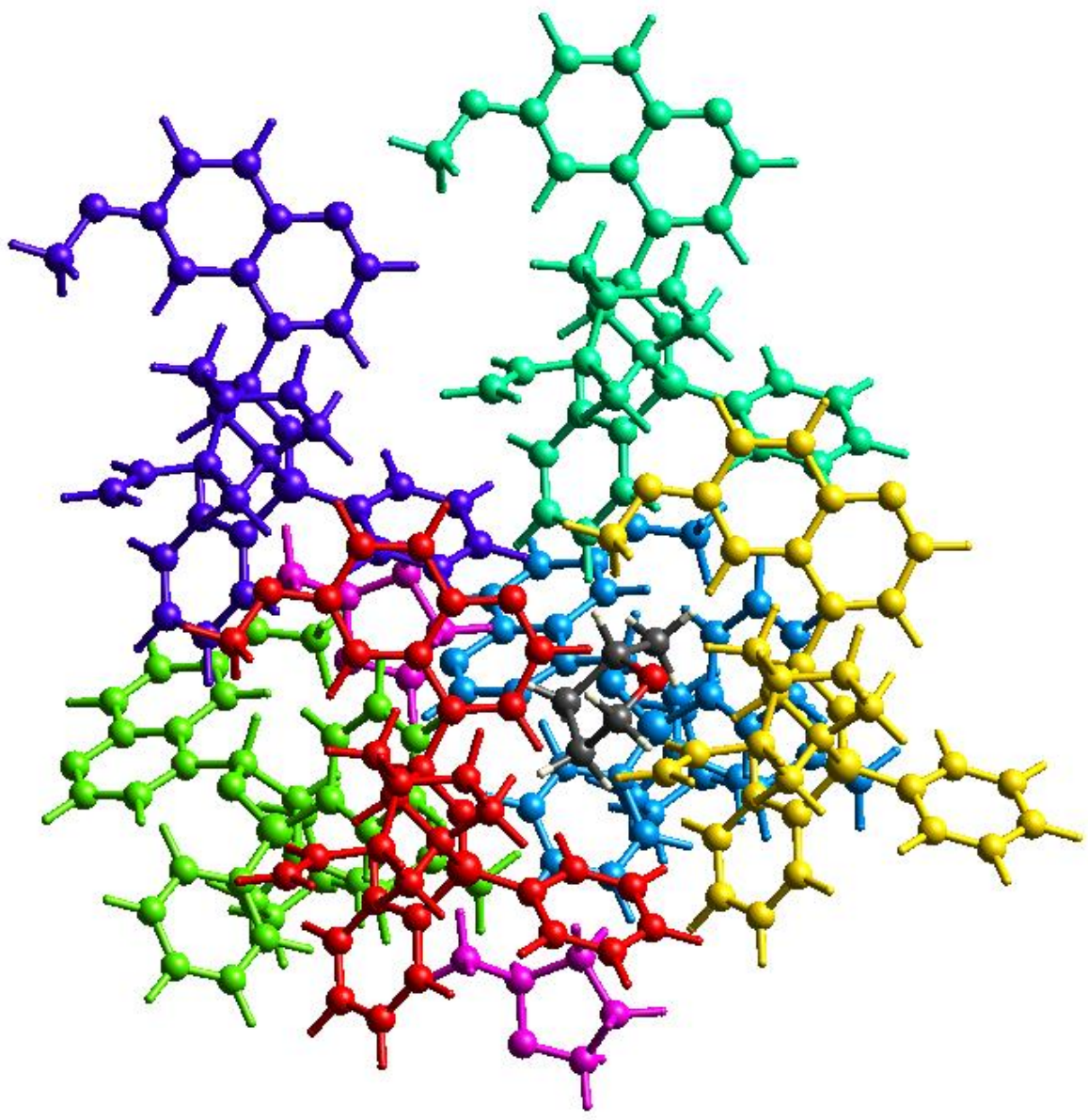

Table S12. Calculated host–guest interaction energies for the model A^S (**4**·MeTHF) with individual neighboring molecules, as well as the total interaction energy, decomposed into electrostatic (E_{ele}), polarization (E_{pol}), dispersion (E_{dis}), and exchange-repulsion (E_{rep}) components. Energies are given in kJ mol^{-1}.

	R [Å]	HS area [Å^2]	E_{ele}	E_{pol}	E_{dis}	E_{rep}	E_{tot}
	4,87	44.4	-25,10	-7,90	-51,40	62,40	-38,60
	7,03	26.2	-17,80	-2,80	-43,20	67,10	-17,10
	6,85	19.9	-10,30	-0,70	-23,50	33,70	-11,00
	9,37	13.5	-3,60	-0,20	-13,70	15,40	-6,40
	9,09	13.5	-2,60	-0,20	-10,50	9,80	-6,10
	10,29	10.5	-2,30	-0,20	-9,50	8,00	-5,90
	7,22	3.8	0,10	0,00	-2,50	0,20	-1,90
	7,22	3.5	0,10	0,00	-2,50	0,20	-1,90
		Total:	-61,50	-12,00	-156,80	196,80	**-88,90**

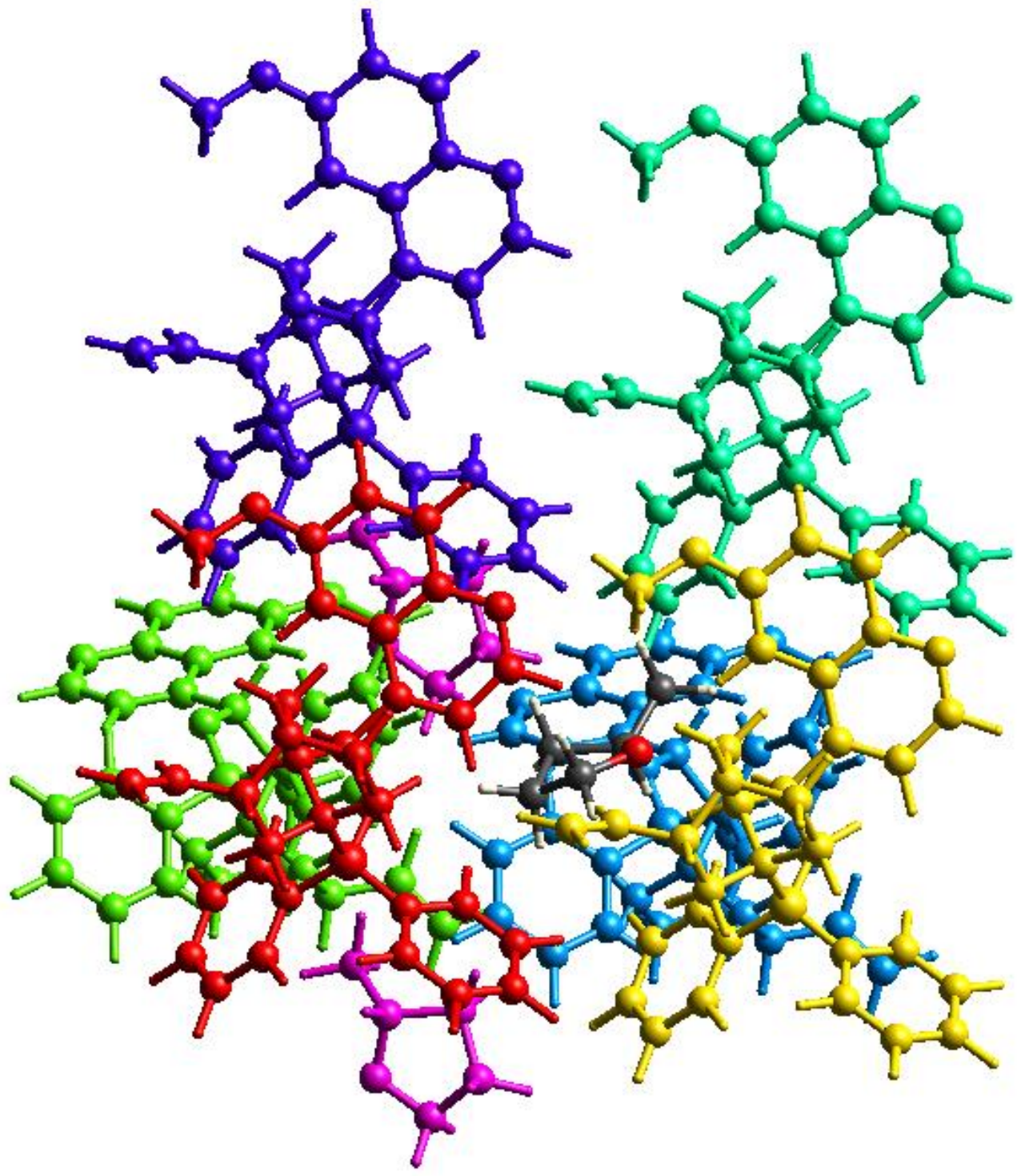

Table S13. Calculated host–guest interaction energies for the model A^R (**4**·MeTHF) with individual neighboring molecules, as well as the total interaction energy, decomposed into electrostatic (E_{ele}), polarization (E_{pol}), dispersion (E_{dis}), and exchange-repulsion (E_{rep}) components. Energies are given in kJ mol^{-1}.

	R [Å]	HS area [Å^2]	E_{ele}	E_{pol}	E_{dis}	E_{rep}	E_{tot}
	4.89	45.5	-22,60	-8,00	-52,40	46,70	-46,60
	7.08	24.1	-8,40	-2,40	-31,30	26,90	-21,30
	6.81	20.3	-11,70	-0,80	-24,40	39,80	-9,60
	9.37	13.4	-2,90	-0,20	-14,00	12,60	-7,60
	9.06	16.3	-4,40	-0,20	-13,50	15,60	-6,90
	10.22	11.4	-2,80	-0,20	-10,50	10,80	-5,50
	7.24	3.8	0,20	0,00	-2,40	0,20	-1,90
	7.24	3.6	0,20	0,00	-2,40	0,20	-1,90
		Total:	-52,40	-11,80	-150,90	152,80	**-101,30**

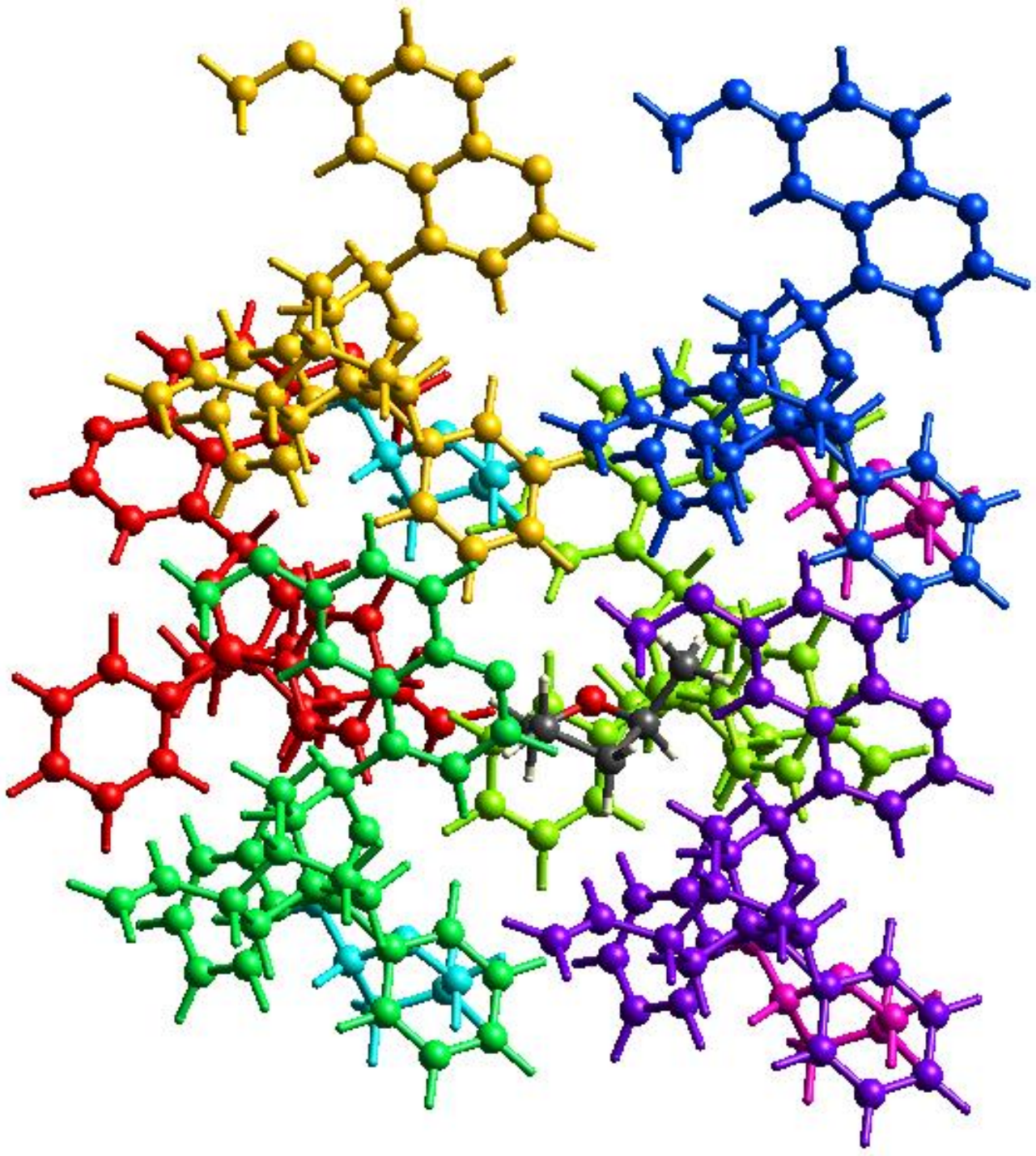

Table S14. Calculated host–guest interaction energies for the model B^S (**4**·MeTHF) with individual neighboring molecules, as well as the total interaction energy, decomposed into electrostatic (E_{ele}), polarization (E_{pol}), dispersion (E_{dis}), and exchange-repulsion (E_{rep}) components. Energies are given in kJ mol^{-1}.

	**R [Å]**	**HS area [Å^2]**	E_{ele}	E_{pol}	E_{dis}	E_{rep}	E_{tot}
	5.17	44.8	-15,90	-3,30	-61,70	70,10	-29,70
	6.51	30.1	-13,40	-5,40	-49,30	78,80	-12,50
	7.25	17.1	-3,30	-0,30	-16,10	10,70	-11,10
	9.13	14.0	-6,80	-0,40	-14,70	16,80	-8,10
	8.83	16.4	-1,70	-0,30	-12,80	8,10	-8,10
	10.19	12.1	0,00	-0,20	-9,10	5,00	-5,00
	7.01	2.8	-0,60	-0,10	-1,60	0,00	-2,10
	7.01	1.8	-0,60	-0,10	-1,60	0,00	-2,10
		Total:	-42,30	-10,10	-166,90	189,50	**-78,70**

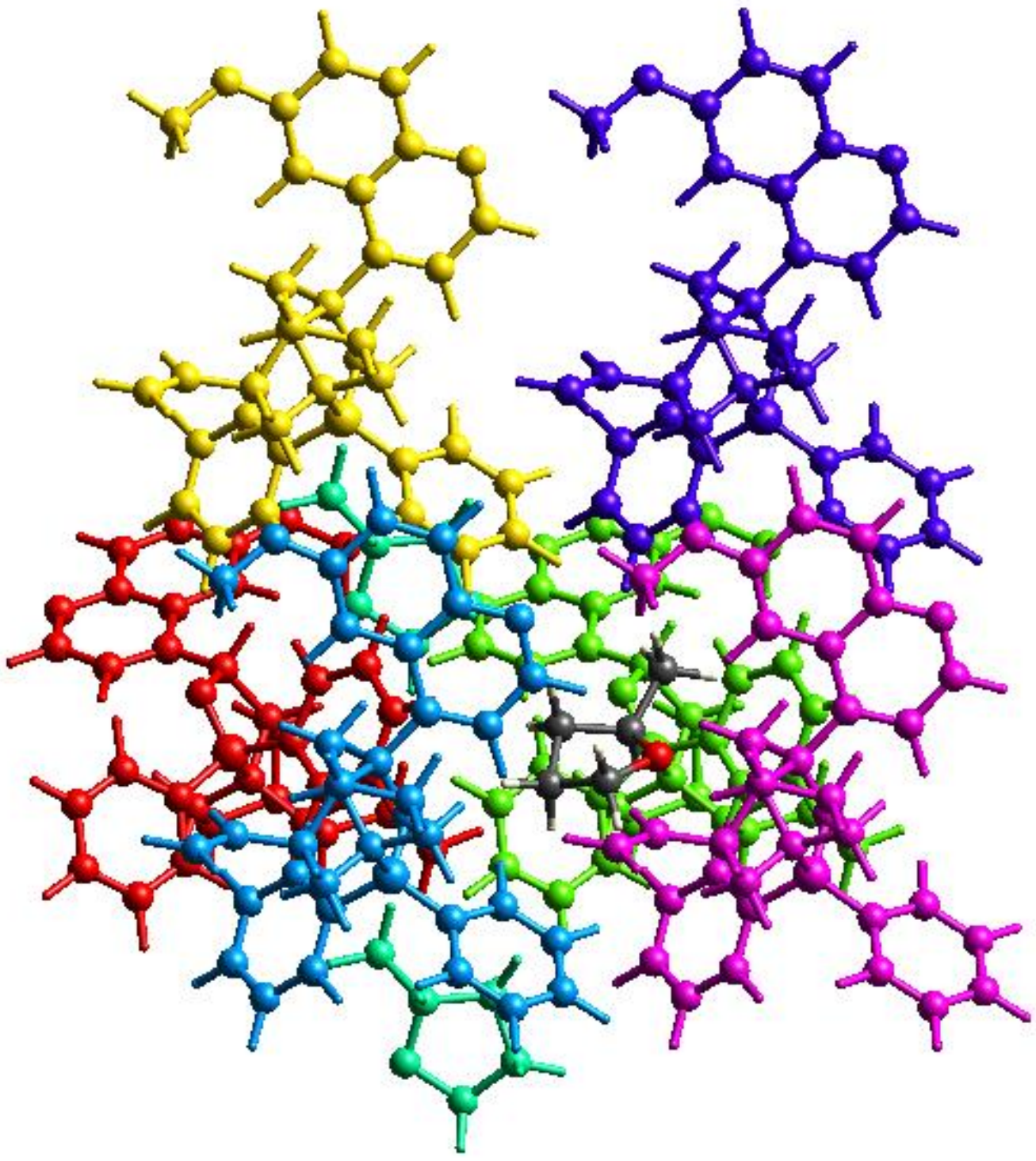

Table S15. Calculated host–guest interaction energies for the model B^R (**4**·MeTHF) with individual neighboring molecules, as well as the total interaction energy, decomposed into electrostatic (E_{ele}), polarization (E_{pol}), dispersion (E_{dis}), and exchange-repulsion (E_{rep}) components. Energies are given in kJ mol^{-1}.

	R [Å]	HS area [Å^2]	E_{ele}	E_{pol}	E_{dis}	E_{rep}	E_{tot}
	4.74	47.0	-25,60	-11,20	-57,80	77,30	-37,90
	6.98	25.6	-10,40	-2,80	-39,70	38,50	-23,90
	6.88	21.2	-5,40	-0,80	-24,40	17,70	-16,60
	9.42	13.6	-2,00	-0,20	-12,00	7,80	-7,80
	9.38	13.5	-1,90	-0,20	-10,00	8,00	-5,90
	10.62	9.8	-0,70	-0,10	-6,60	2,60	-5,00
	7.13	4.2	0,10	0,00	-2,70	0,30	-2,10
	7.13	4.1	0,10	0,00	-2,70	0,30	-2,10
		Total:	-45,80	-15,30	-155,90	152,50	**-101,30**

Results for 2·0.5MeTHF.

In this case, the experimental crystal structure is best represented by Model A^S, including (S)-guest. Similarly to the **4**·MeTHF system, HS analysis using the fragment patch maps shows that the guest molecules are in contact mainly with eight neighboring molecules: six host molecules and two other guest molecules (Fig. S29a), which were therefore included in the interaction energy calculations. The contact maps and energy decomposition show a similar dominance of H···H and H···C/C···H contacts (Fig. S29bc), together with a major contribution from dispersion forces (Table S17). In contrast to **4**·MeTHF, however, the guest molecules in the voids of **2**·0.5MeTHF are considerably less tightly packed, as reflected by the higher mean d_i and d_{norm} values of 1.557 and 0.630 Å, respectively, and the larger HS volume of 139.72 Å³. Consequently, the docking simulations sampled a much broader range of possible guest positions, with the optimal Model B structures differing substantially from Model A for both enantiomers (Fig. S30 and S31). These simulated poses exhibit increased steric hindrance but, at the same time, enhanced dispersion interactions, likely resulting from more favorable contacts with the aromatic rings (Tables S18 and S19). As a result, the total interaction energies remain comparable to those observed for the models A. Overall, the differences in total interaction energies between the two enantiomers are relatively small, falling within the range of 2.6-7.9 kJ mol^{-1}, which is consistent with the low degree of enantiodiscrimination observed in the separation experiments.

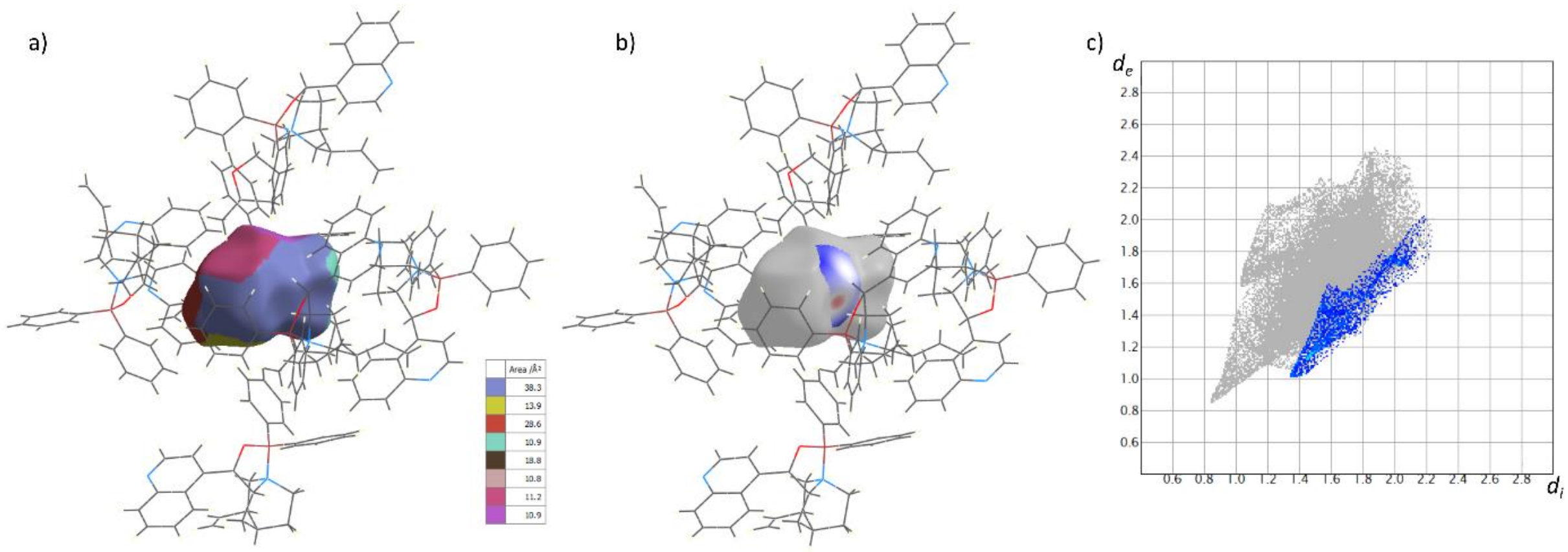

Fig. S29. Hirshfeld surface analysis of the guest molecule in Model A^R of the **2**·0.5MeTHF system: (a) fragment patch map; (b) d_{norm} map highlighting the CH···O contacts; (c) fingerprint plot of d_e/d_i distances highlighting the CH···O contacts.

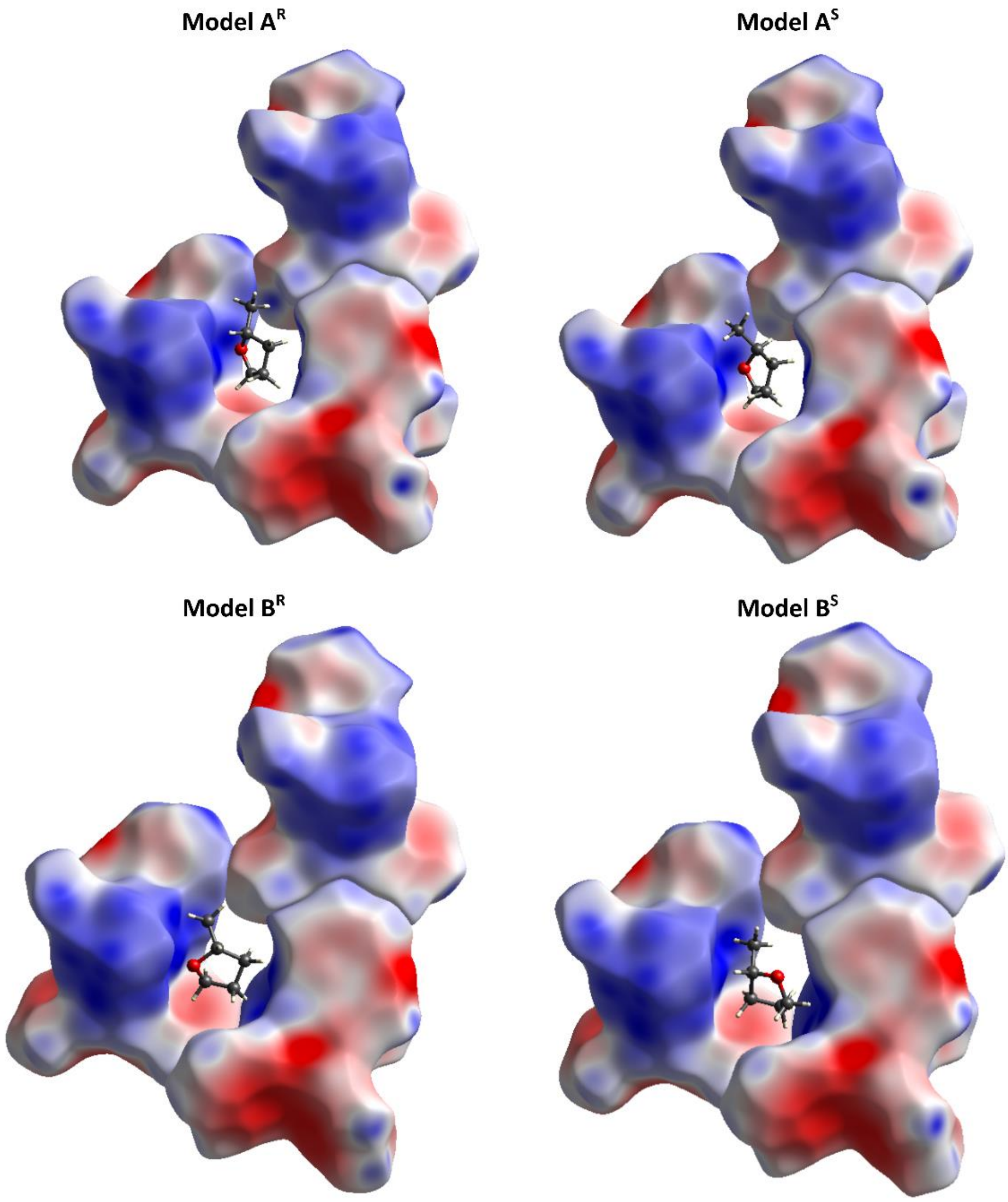

Fig. S30. Positions of the guest molecules within the voids in Models A and B of the **2**·0.5MeTHF system. The Hirshfeld surfaces of the host molecules are mapped with the ESP over the range from -0.05 au (red) to +0.05 au (blue).

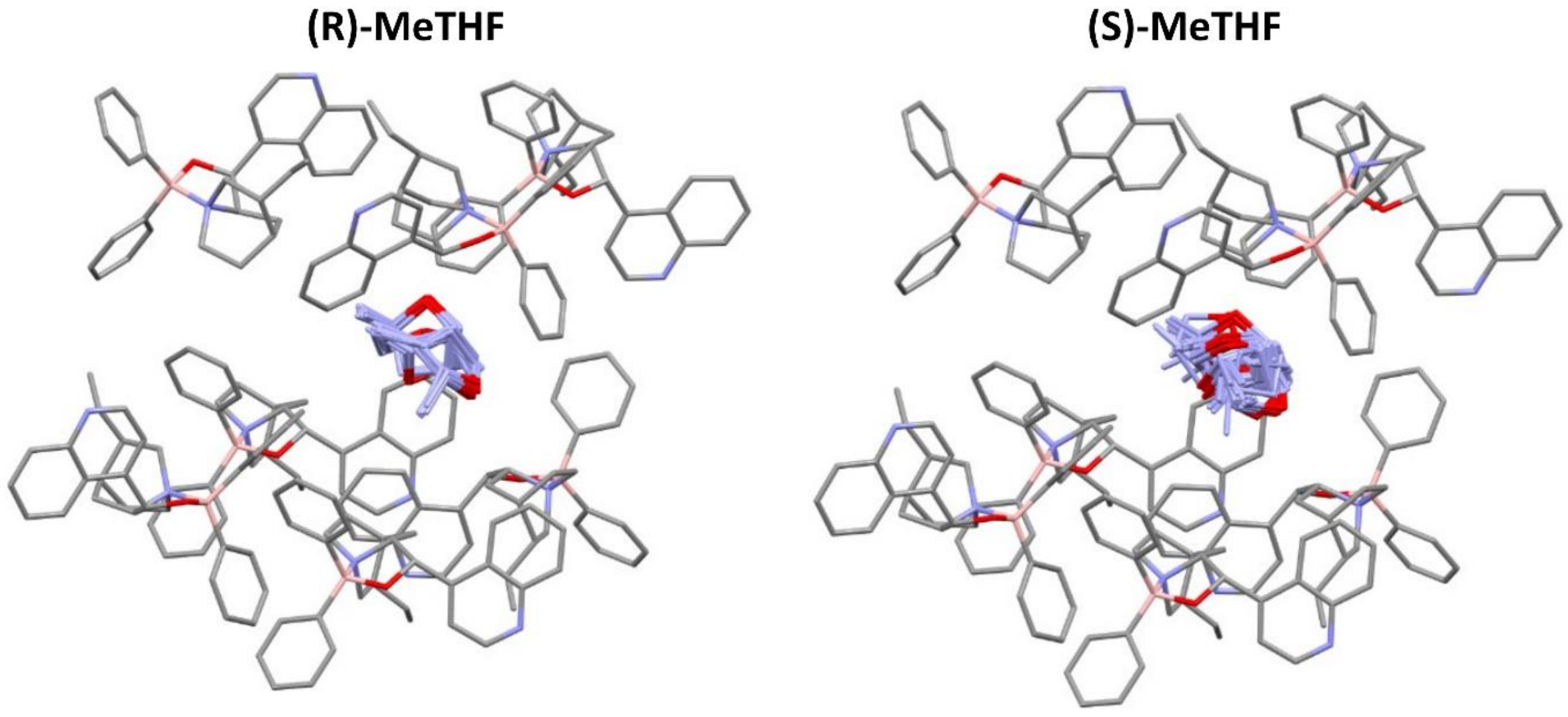

Fig. S31. Simulated poses of (R)- and (S)-MeTHF within the voids of **2**·0.5MeTHF, obtained from molecular docking calculations.

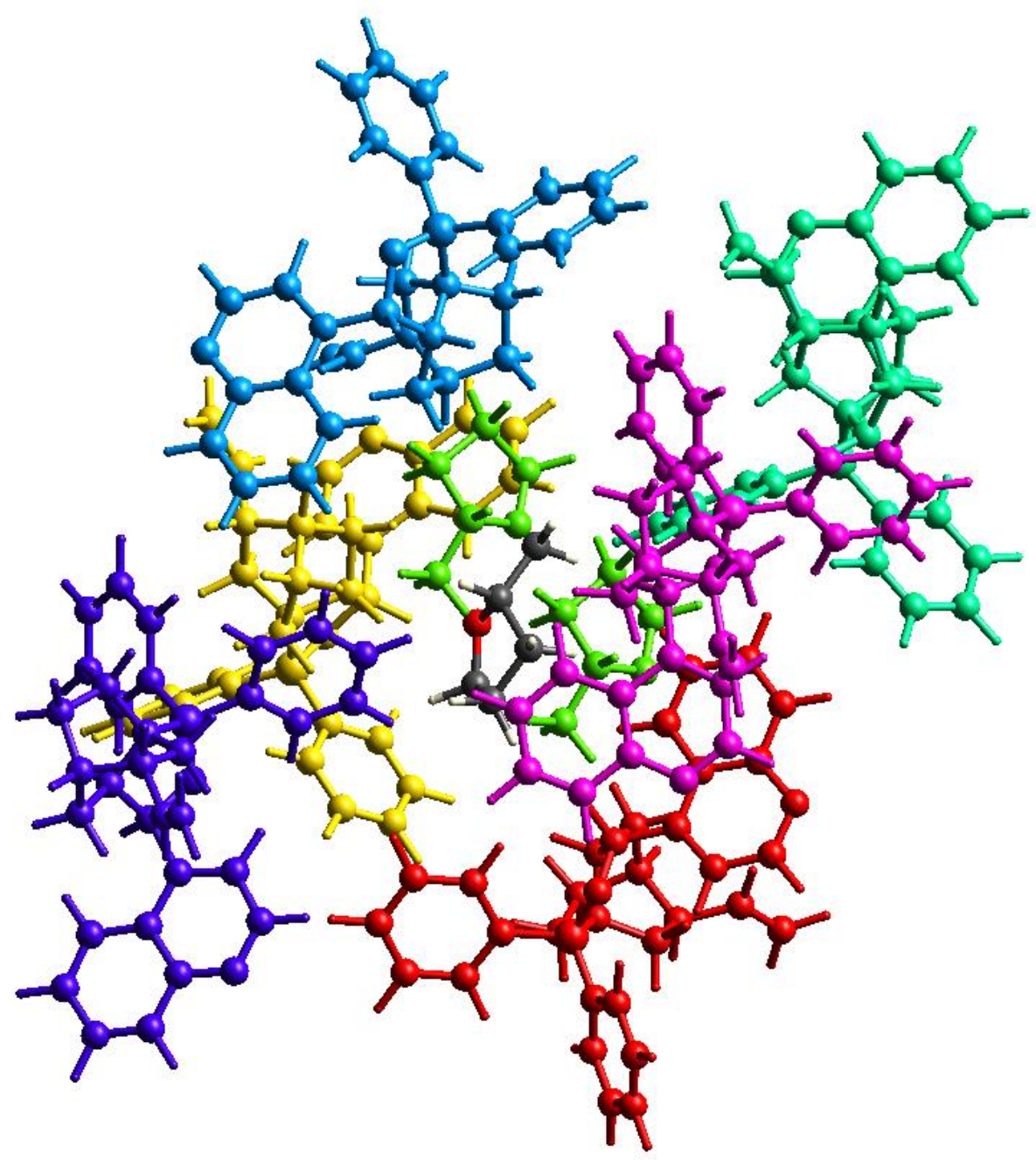

Table S16. Calculated host–guest interaction energies for the model A^R (**2**·0.5MeTHF) with individual neighboring molecules, as well as the total interaction energy, decomposed into electrostatic (E_{ele}), polarization (E_{pol}), dispersion (E_{dis}), and exchange-repulsion (E_{rep}) components. Energies are given in kJ mol^{-1}.

	R [Å]	HS area [Å^2]	E_{ele}	E_{pol}	E_{dis}	E_{rep}	E_{tot}
	5,35	39.9	-20,8	-6,4	-42,1	35	-41,7
	6,67	30.1	-3,5	-1,2	-25,1	19,6	-14,3
	7,08	18.9	-3,5	-0,4	-19,3	16	-10,9
	8,42	9.4	-0,3	-0,3	-6,4	1	-5,5
	5,98	9.7	-1,2	-0,2	-4,6	1,2	-4,8
	5,98	8.7	-1,2	-0,2	-4,6	1,2	-4,8
	9,87	11.9	-8,3	-0,2	-14	32,8	-0,8
	9,13	14.8	-4,9	-0,5	-10,5	23,7	-0,1
		Total:	-43,70	-9,40	-126,60	130,50	**-82,90**

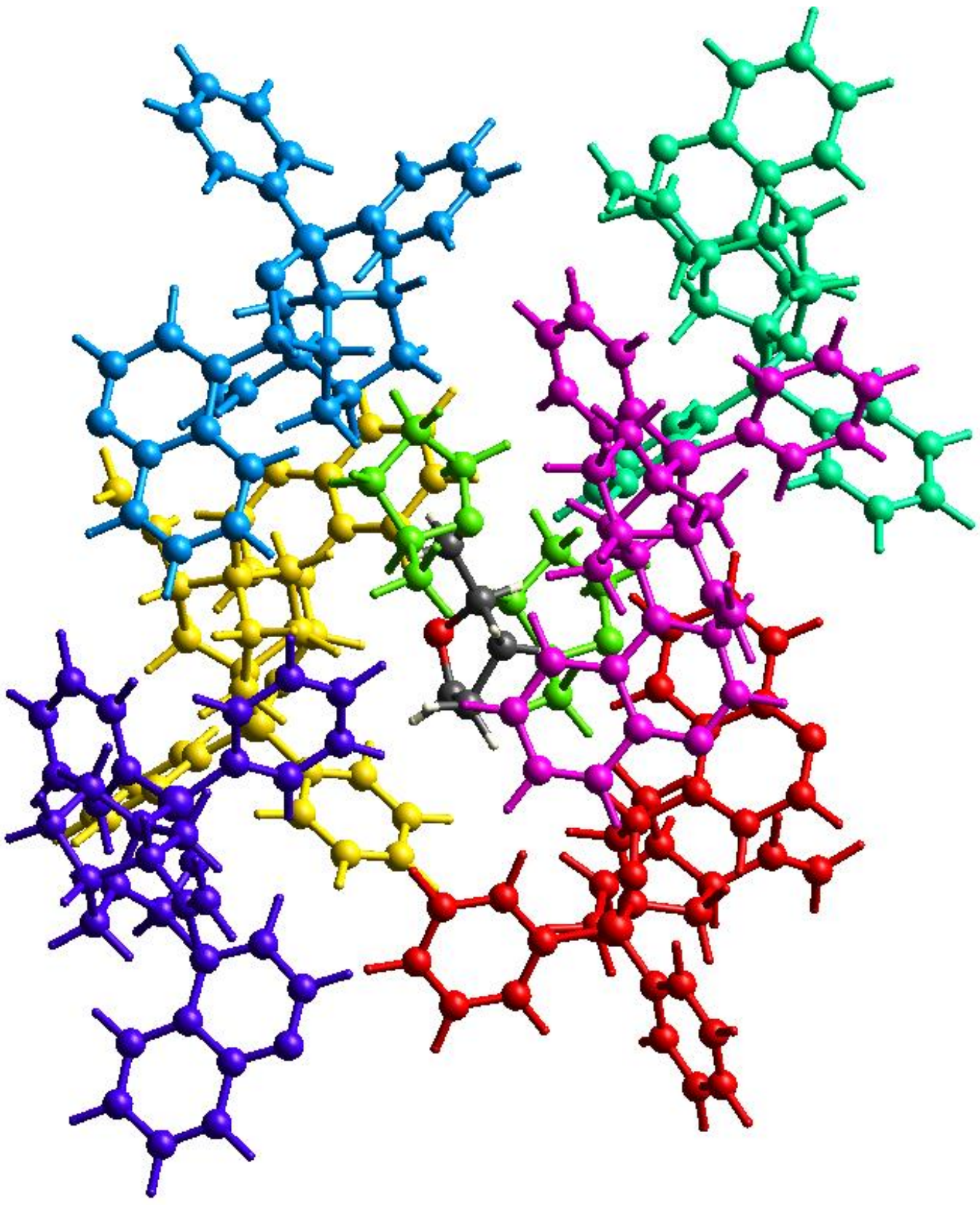

Table S17. Calculated host–guest interaction energies for the model A^S (**2**·0.5MeTHF) with individual neighboring molecules, as well as the total interaction energy, decomposed into electrostatic (E_{ele}), polarization (E_{pol}), dispersion (E_{dis}), and exchange-repulsion (E_{rep}) components. Energies are given in kJ mol^{-1}.

	**R [Å]**	**HS area [Å^2]**	E_{ele}	E_{pol}	E_{dis}	E_{rep}	E_{tot}
(yellow)	5,33	38.2	-21,1	-6,3	-41,3	34,9	-41,5
(magenta)	7,08	18.8	-2,2	-0,3	-18,4	11,2	-11,7
(red)	6,71	28.6	-5,1	-1,2	-23,4	25,6	-10,8
(blue)	8,38	10.9	-0,9	-0,4	-8,4	2,8	-6,9
(teal)	9,92	10.9	-1	-0,1	-8,3	4,3	-5,7
(green)	6.00	11.2	-1,5	-0,2	-6,3	3,3	-5,2
(green)	6.00	10.8	-1,5	-0,2	-6,3	3,3	-5,2
(violet)	9,06	13.9	-7	-0,5	-11,4	31	1,5
		Total:	-40,30	-9,20	-123,80	116,40	**-85,50**

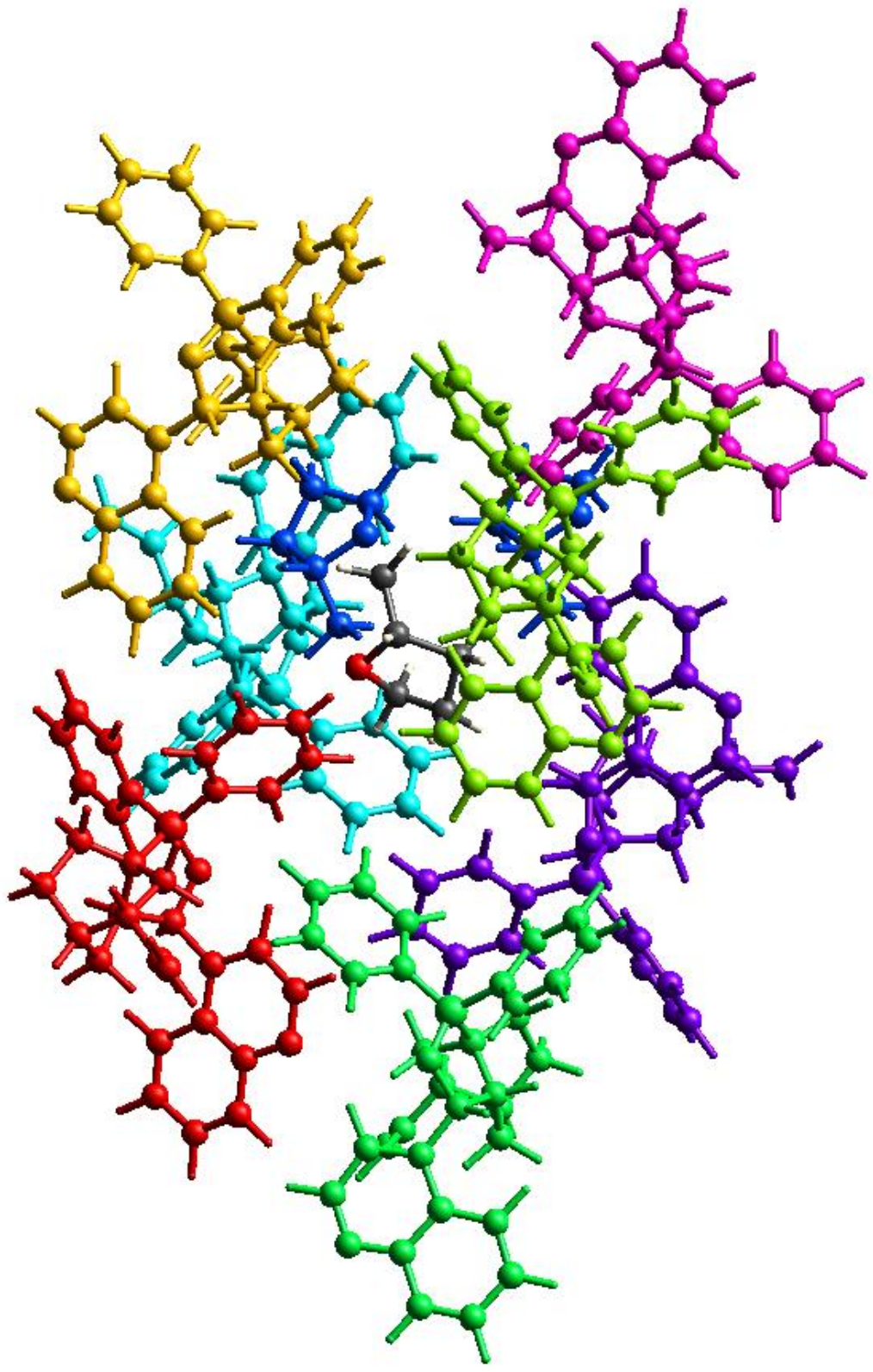

Table S18. Calculated host–guest interaction energies for the model B^R^ (**2**·0.5MeTHF) with individual neighboring molecules, as well as the total interaction energy, decomposed into electrostatic (E_{ele}), polarization (E_{pol}), dispersion (E_{dis}), and exchange-repulsion (E_{rep}) components. Energies are given in kJ mol^{-1}.

	**R [Å]**	**HS area [Å^2]**	E_{ele}	E_{pol}	E_{dis}	E_{rep}	E_{tot}
	5,40	41.6	-23,70	-7,00	-48,60	50,10	-41,60
	6,61	31.2	-4,30	-1,30	-29,40	23,50	-16,60
	7,12	21.4	-5,20	-0,60	-21,70	17,30	-14,10
	8,57	10.6	-0,40	-0,30	-6,80	2,00	-5,40
	10,69	8.1	-0,20	0,00	-3,20	0,20	-2,90
	6,55	9.0	-0,20	-0,10	-2,70	0,10	-2,50
	6,55	8.7	-0,20	-0,10	-2,70	0,10	-2,50
	8,22	16.6	-5,10	-3,60	-24,40	46,10	-0,80
		Total:	-39,30	-13,00	-139,50	139,40	**-86,40**

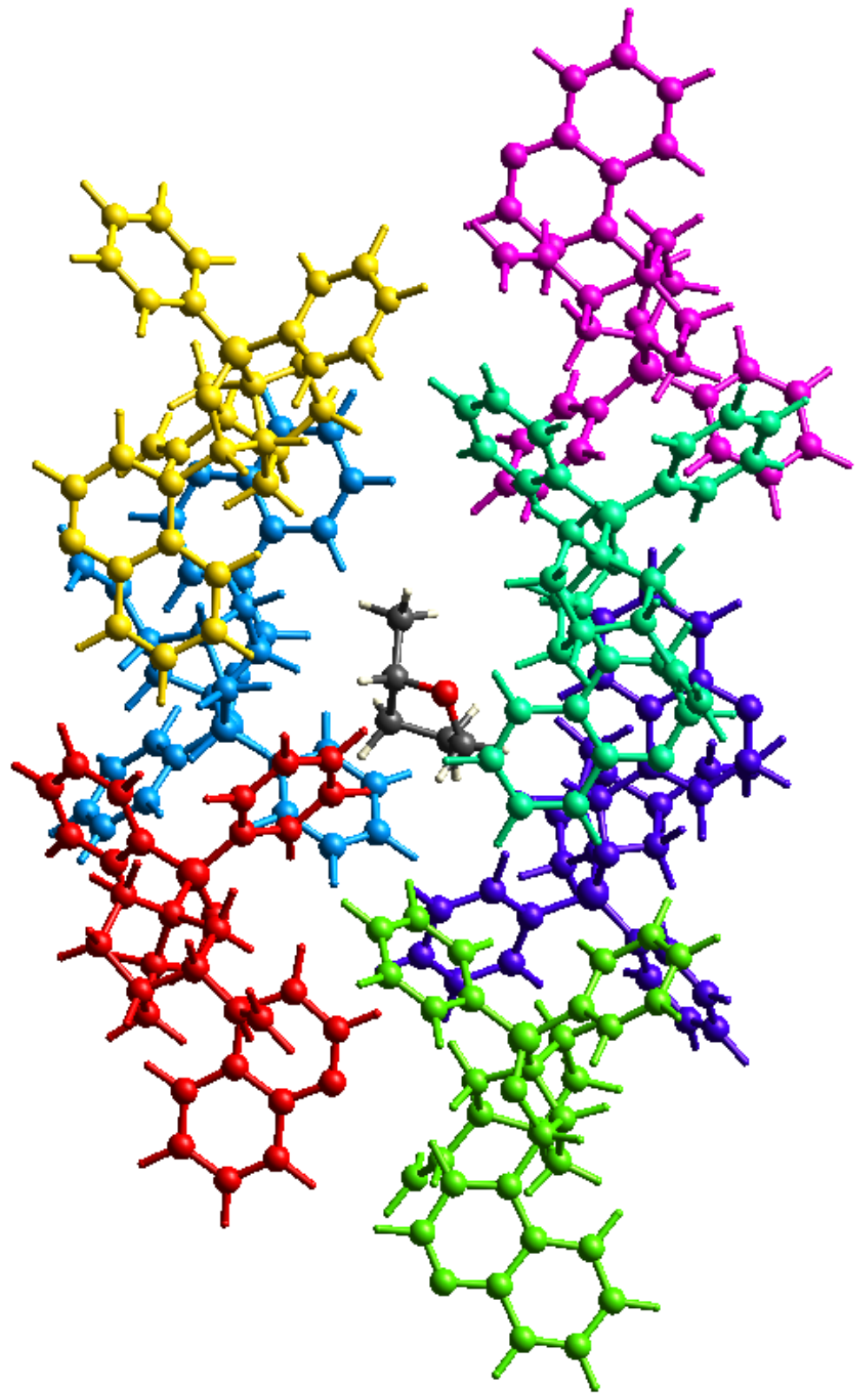

Table S19. Calculated host–guest interaction energies for the model B[S] (**2**·0.5MeTHF) with individual neighboring molecules, as well as the total interaction energy, decomposed into electrostatic (E_{ele}), polarization (E_{pol}), dispersion (E_{dis}), and exchange-repulsion (E_{rep}) components. Energies are given in kJ mol^{-1}.

	R [Å]	HS area [Å²]	E_{ele}	E_{pol}	E_{dis}	E_{rep}	E_{tot}
	5,60	48.3	-17,1	-4,6	-57,5	66,7	-30,4
	5,95	37.0	-10	-2,6	-39,3	45,8	-18,4
	7,24	17.3	-7,8	-1,7	-22,8	30,4	-10,7
	8,33	15.1	-9,2	-0,7	-20,4	30,8	-8,9
	10,79	10.1	-0,6	-0,1	-3,4	0,6	-3,2
	9,24	5.3	-0,3	-0,1	-3,1	0,1	-3,1
	10.76	6.8	-0,20	-0,10	-1,80	0,00	-1,90
	10.76	6.5	-0,20	-0,10	-1,80	0,00	-1,90
		Total:	-45,40	-10,00	-150,10	174,40	**-78,50**

Results for 4·(3-MCH).

In this case, the experimental crystal structure is best represented by Model A^S, including (S)-guest enantiomer. Similarly to the **4**·MeTHF system, HS analysis using the fragment patch maps shows that the guest molecules are in contact mainly with eight neighboring molecules: six host molecules and two other guest molecules, which were therefore included in the interaction energy calculations (Fig. S32a). The contact maps and interaction energy decomposition reveal a similar dominance of H···H and H···C/C···H contacts and a major contribution from dispersion forces (Fig. S32bc). However, compared with **4**·MeTHF, the contribution of the polarization energy is significantly higher, particularly for the interaction with main host molecule (Table S21). This may be attributed to the more strongly polarized and sterically accessible carbonyl group, which promotes directional O···H and electrostatic interactions with the host. At the same time, the guest molecules in **4**·(3-MCH) are more tightly packed than those in **4**·MeTHF, as reflected by the lower mean d_{norm} value of 0.564 Å, respectively. This tighter packing also results in substantially larger differences in the repulsion energy between the two enantiomers than observed for MeTHF (65.2 vs 44.0 kJ mol^{-1}) (Table S12-S13 and S20-21).

The poses obtained from the docking simulations for both enantiomers cluster mainly around a single position that deviate significantly from corresponding Model A (Fig. S34). The optimal poses in Models B tend to orient the carbonyl group of the guest toward the Ph_2B cavity, characterized by a negative ESP, likely maximizing interactions with the aromatic rings (Fig. S33). This arrangement is inconsistent with the electrostatic complementarity observed in the experimental structure and results in a reduced electrostatic contribution to the interaction energy (Tables S20-23). At the same time, it facilitates dispersion interactions, maintaining the total attractive binding energy at a level comparable to that of the corresponding Models A. Nevertheless, Model B poses exhibit substantially higher repulsion energies due to steric hindrance, leading to significantly less favorable total interaction energies. Thus, the simulated positions within models B further highlights the limitations of CCDC GOLD docking for this system, particularly in accounting for weak, directional O···H interactions that appear to play an important role in stabilizing the experimentally observed host–guest arrangement.

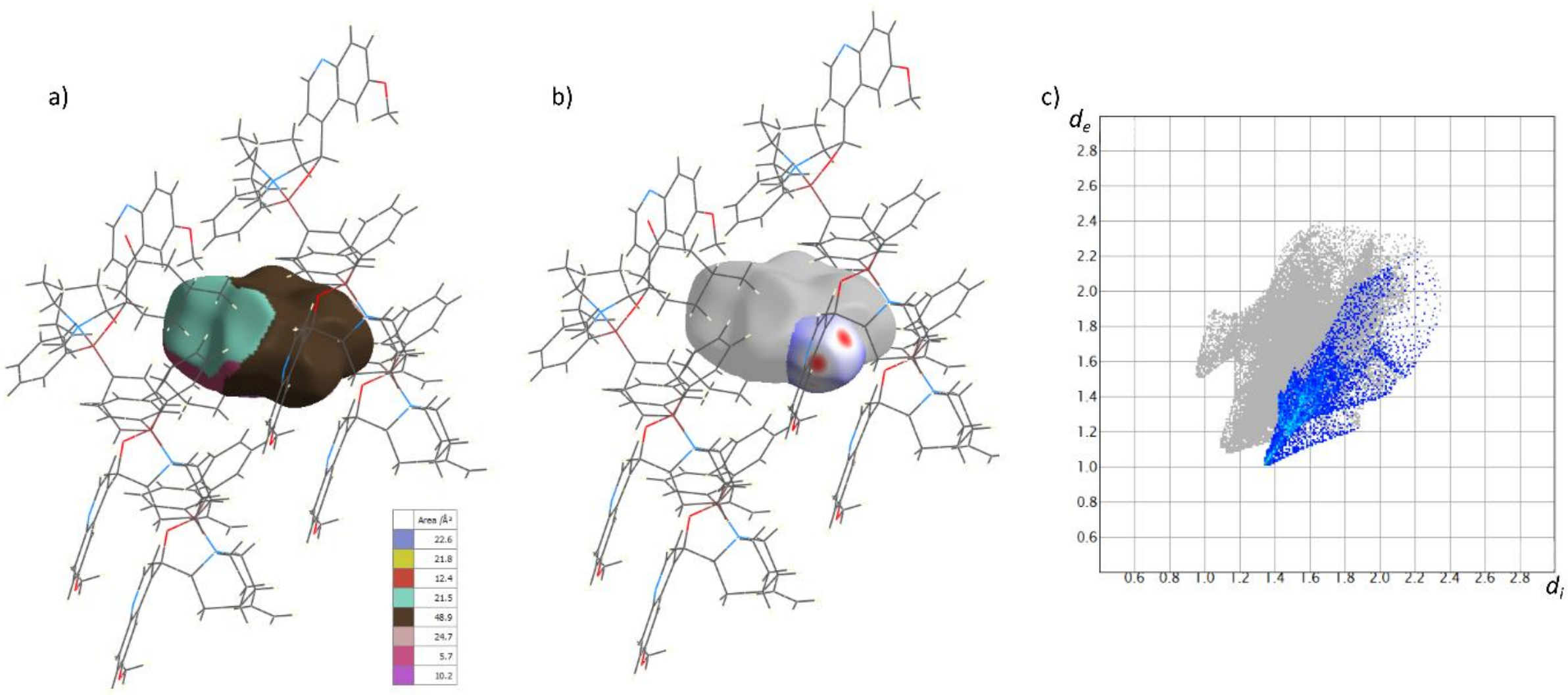

Fig. S32. Hirshfeld surface analysis of the guest molecule in Model A^R of the **4**·(3-MCH) system: (a) fragment patch map; (b) d_{norm} map highlighting the CH···O contacts; (c) fingerprint plot of d_e/d_i distances highlighting the CH···O contacts.

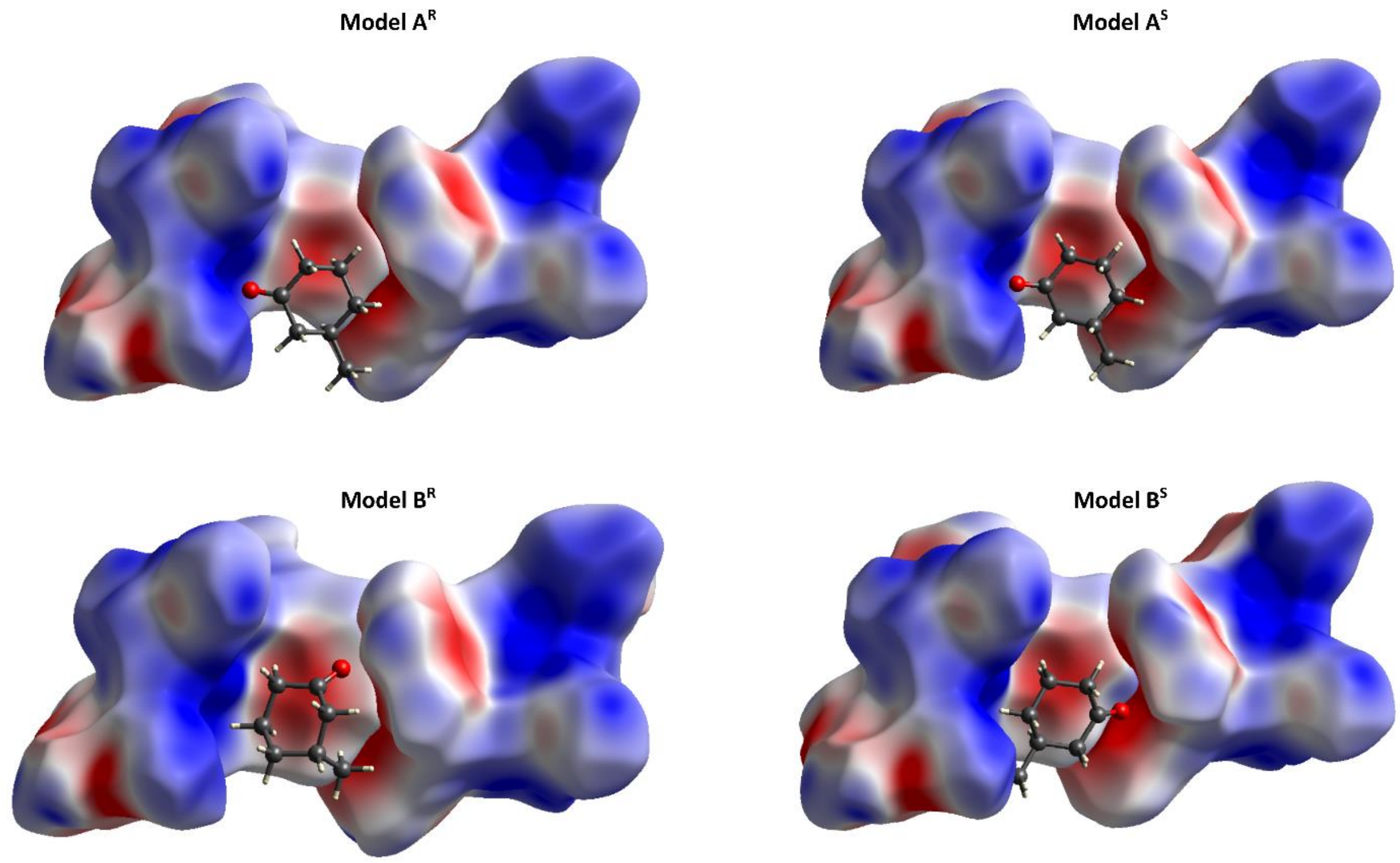

Fig. S33. Positions of the guest molecules within the voids in Models A and B of the **4**·(3-MCH) system. The Hirshfeld surfaces of the host molecules are mapped with the ESP over the range from -0.05 au (red) to +0.05 au (blue).

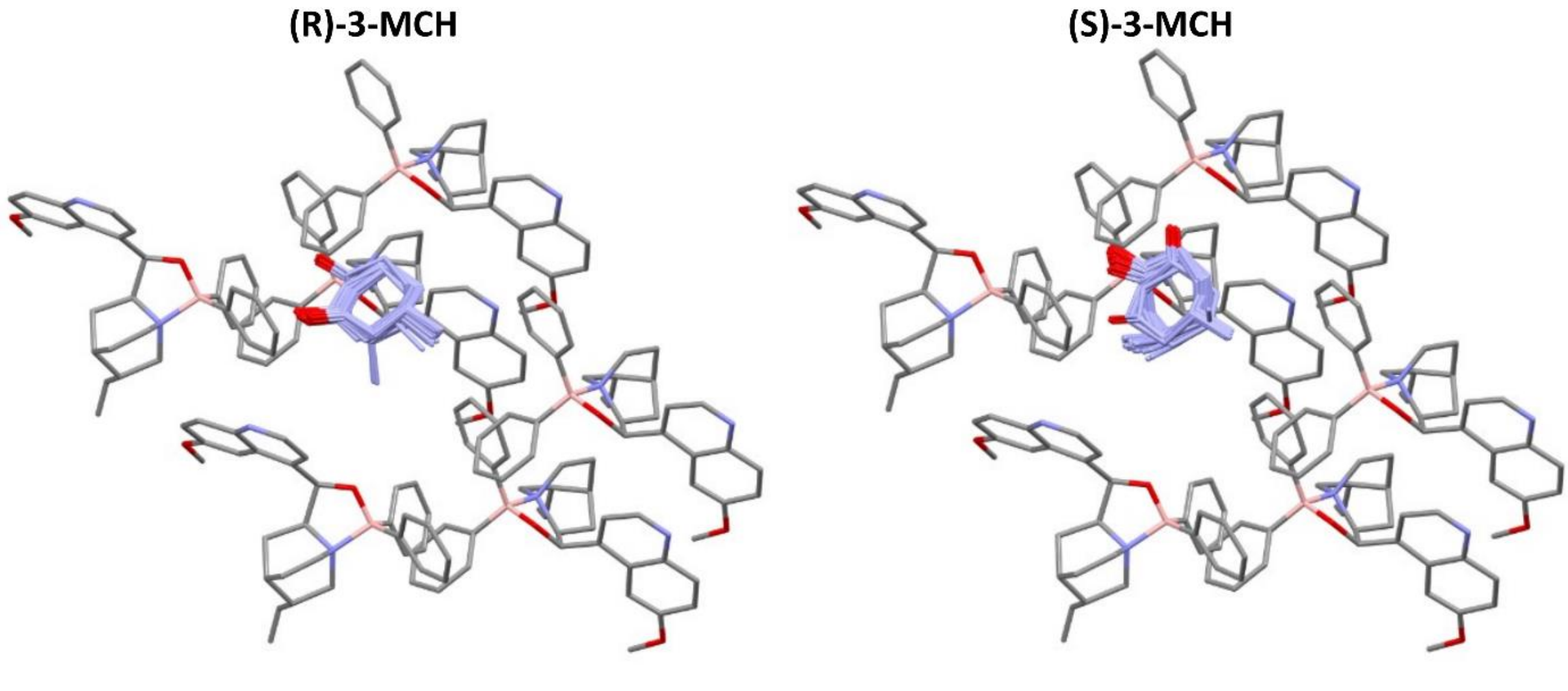

Fig. S34. Simulated poses of (R)- and (S)-MeTHF within the voids of **4**·(3-MCH), obtained from molecular docking calculations.

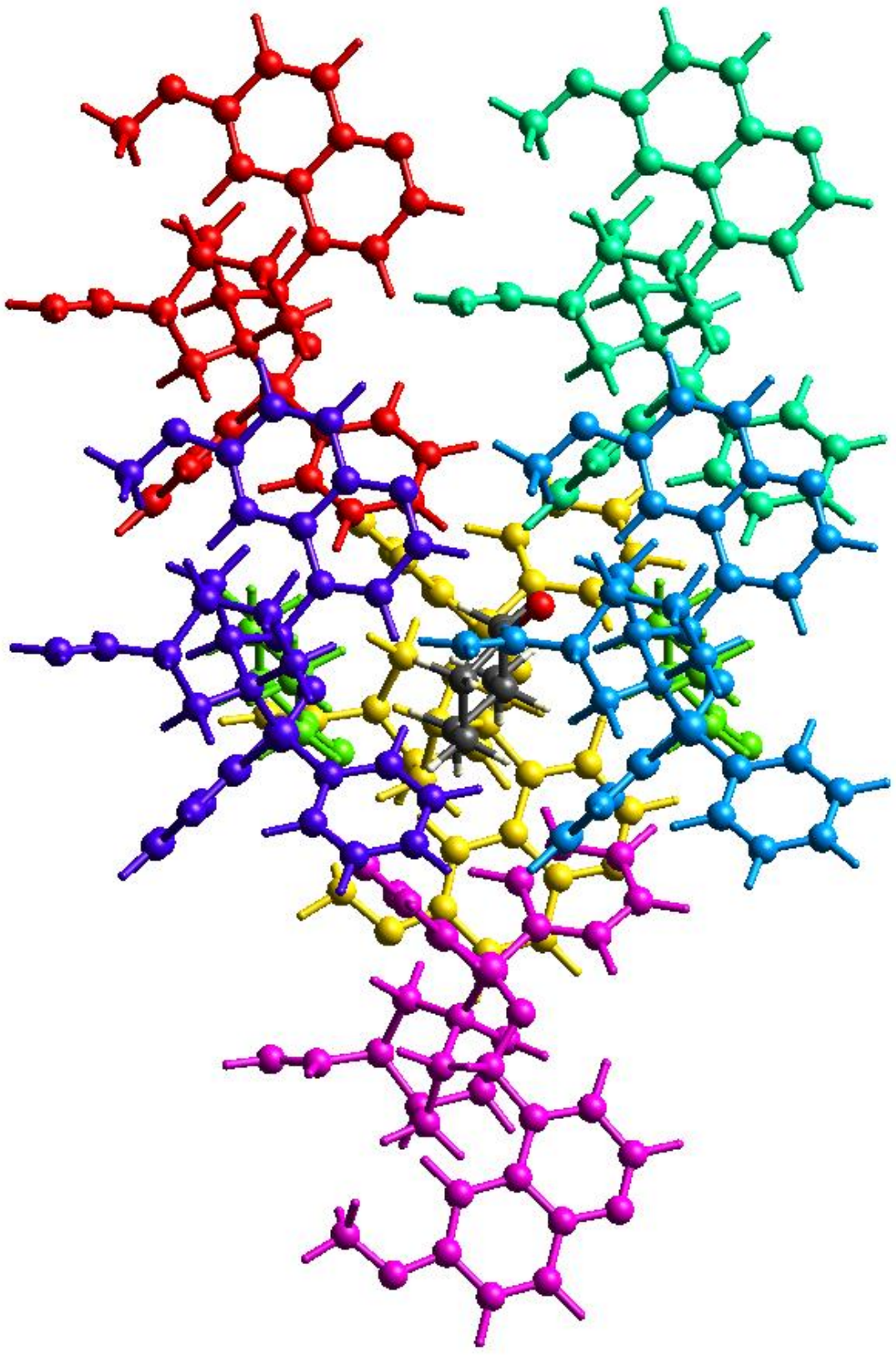

Table S20. Calculated host–guest interaction energies for the model A[R] (**4**·(3-MCH)) with individual neighboring molecules, as well as the total interaction energy, decomposed into electrostatic (E_{ele}), polarization (E_{pol}), dispersion (E_{dis}), and exchange-repulsion (E_{rep}) components. Energies are given in kJ mol^{-1}.

	R [Å]	HS area [Å^2]	E_{ele}	E_{pol}	E_{dis}	E_{rep}	E_{tot}
	5,58	46.2	-38,5	-10,9	-46,8	72,5	-44,7
	6,39	24.4	-17,4	-2,3	-35,5	52,3	-18,8
	8,71	23.4	-5,9	-1,9	-23,3	16	-18,1
	8.00	15.2	-1,4	-0,3	-13,4	6,3	-9,5
	6,26	19.4	-2,4	-0,4	-12,3	12,5	-5,9
	6,26	19.1	-2,4	-0,4	-12,3	12,5	-5,9
	9,87	6.5	-1,6	-0,3	-4,5	0,4	-5,6
	10,35	12.0	-6	-0,7	-11,1	20,4	-3,9
		Total:	-75,60	-17,20	-159,20	192,90	**-112,40**

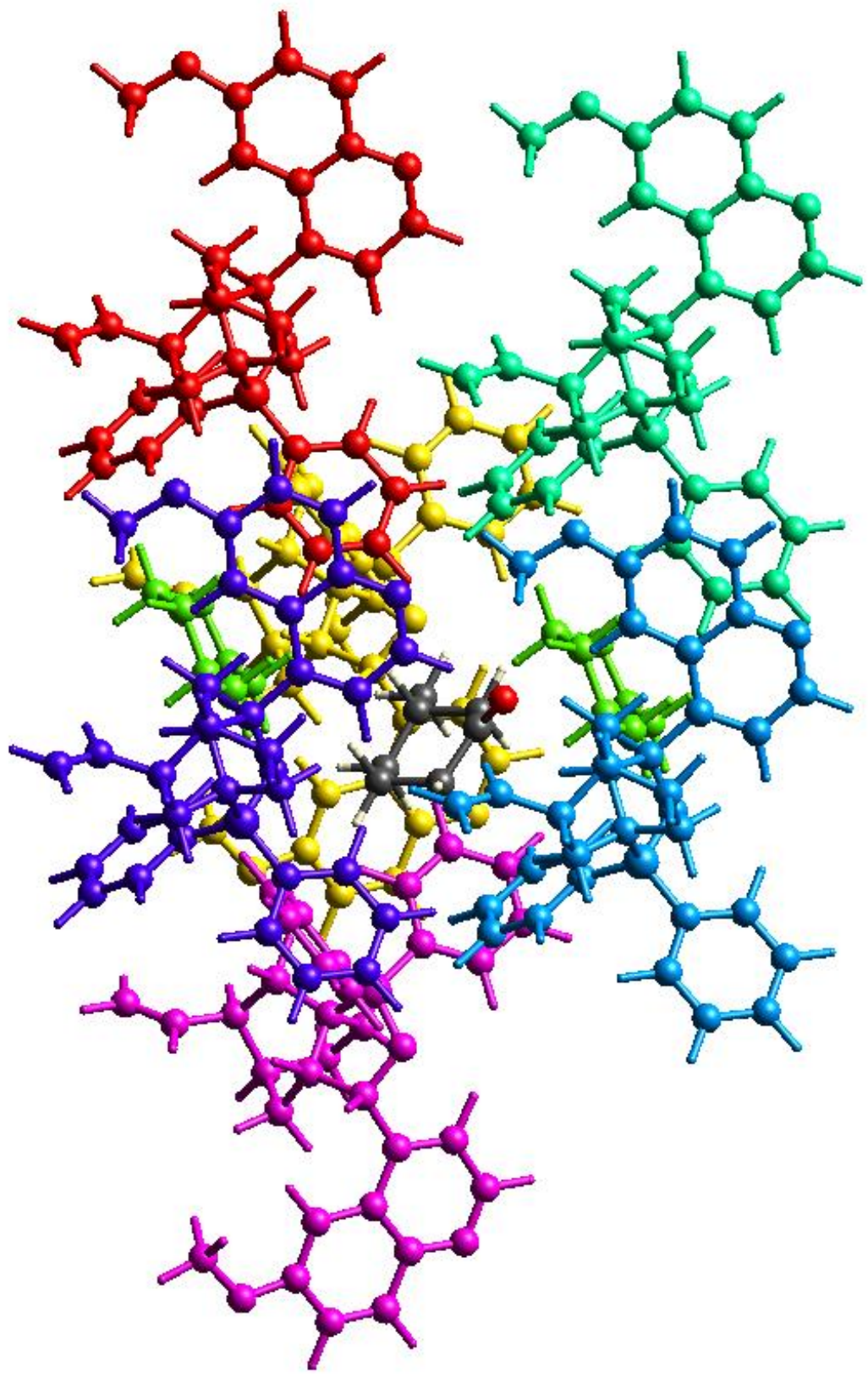

Table S21. Calculated host–guest interaction energies for the model A^{S} (**4**•(3-MCH)) with individual neighboring molecules, as well as the total interaction energy, decomposed into electrostatic (E_{ele}), polarization (E_{pol}), dispersion (E_{dis}), and exchange-repulsion (E_{rep}) components. Energies are given in kJ mol^{-1}.

	**R** [Å]	**HS area** [Å^2]	E_{ele}	E_{pol}	E_{dis}	E_{rep}	E_{tot}
	5,49	48.9	-38,2	-11,6	-55,3	63,6	-57,8
	6,50	24.7	-6,4	-2,3	-27,9	16,3	-22,7
	8,62	22.6	-6,7	-1,8	-22,6	15,1	-18,8
	6,27	21.5	-2,4	-0,4	-16,9	12,5	-9,8
	6,27	21.8	-2,4	-0,4	-16,9	12,5	-9,8
	8.00	12.4	-1,2	-0,3	-12,6	5,5	-9
	10,50	10.2	-1,4	-0,4	-6,6	1,6	-6,5
	9,91	5.7	-2	-0,3	-4,4	0,6	-5,8
		Total:	-60,70	-17,50	-163,20	127,70	-140,20

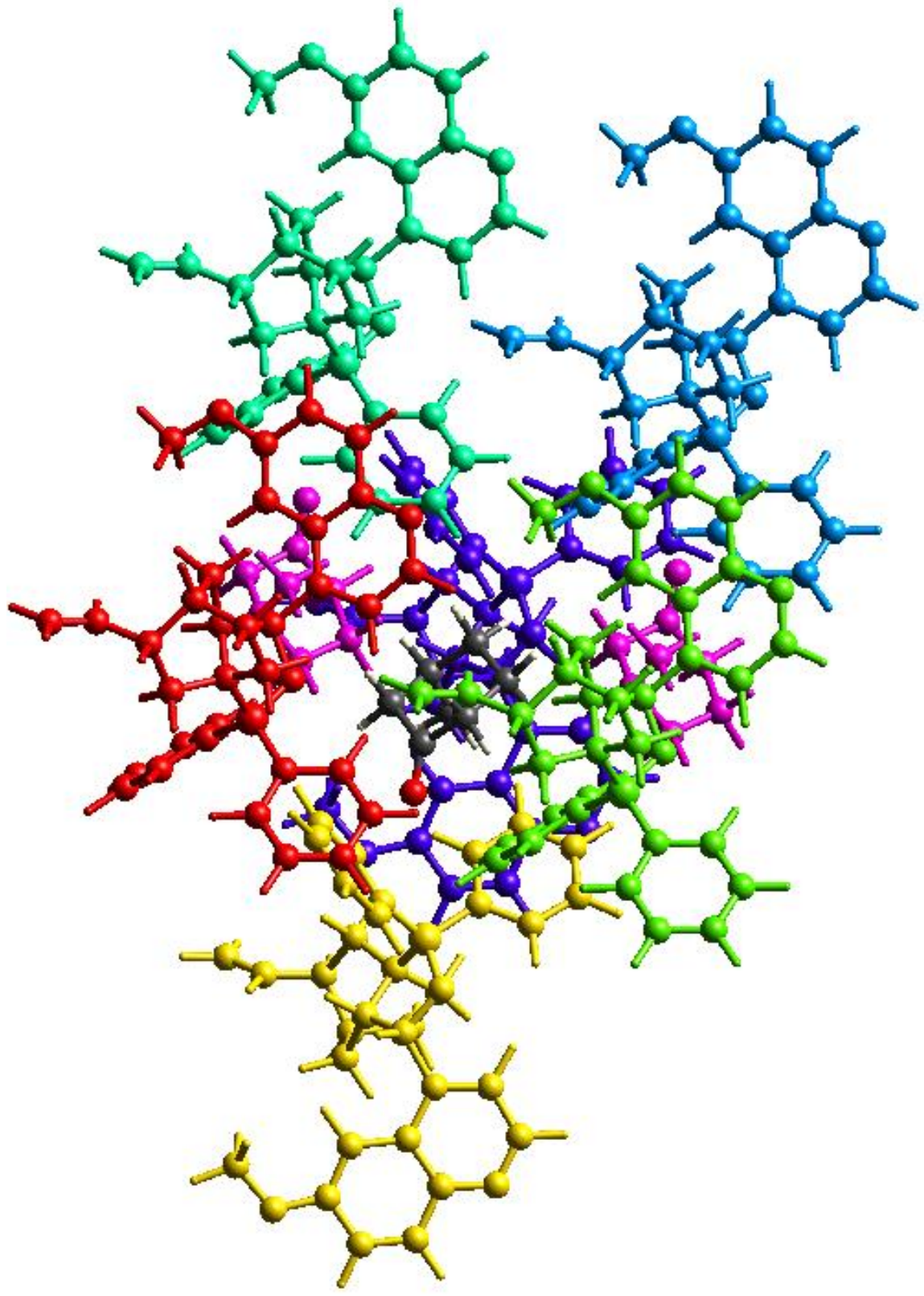

Table S22. Calculated host–guest interaction energies for the model B^R (**4**•(3-MCH)) with individual neighboring molecules, as well as the total interaction energy, decomposed into electrostatic (E_{ele}), polarization (E_{pol}), dispersion (E_{dis}), and exchange-repulsion (E_{rep}) components. Energies are given in kJ mol^{-1}.

	R [Å]	HS area [Å^2]	E_{ele}	E_{pol}	E_{dis}	E_{rep}	E_{tot}
	5.59	52.8	-21,90	-11,10	-71,10	115,70	-21,90
	5.65	32.8	-13,50	-5,40	-54,10	77,50	-17,60
	10.97	14.7	-3,80	-0,30	-11,10	12,30	-6,30
	8.75	10.6	-0,40	-0,10	-4,10	0,10	-4,00
	7.53	15.1	0,00	-0,10	-4,00	0,40	-3,30
	7.53	14.5	0,00	-0,10	-4,00	0,40	-3,30
	10.94	2.8	0,40	-0,10	-2,20	0,00	-1,50
	8.14	27.2	2,30	-6,70	-25,80	44,30	2,30
		Total:	-36,90	-23,90	-176,40	250,70	-55,60

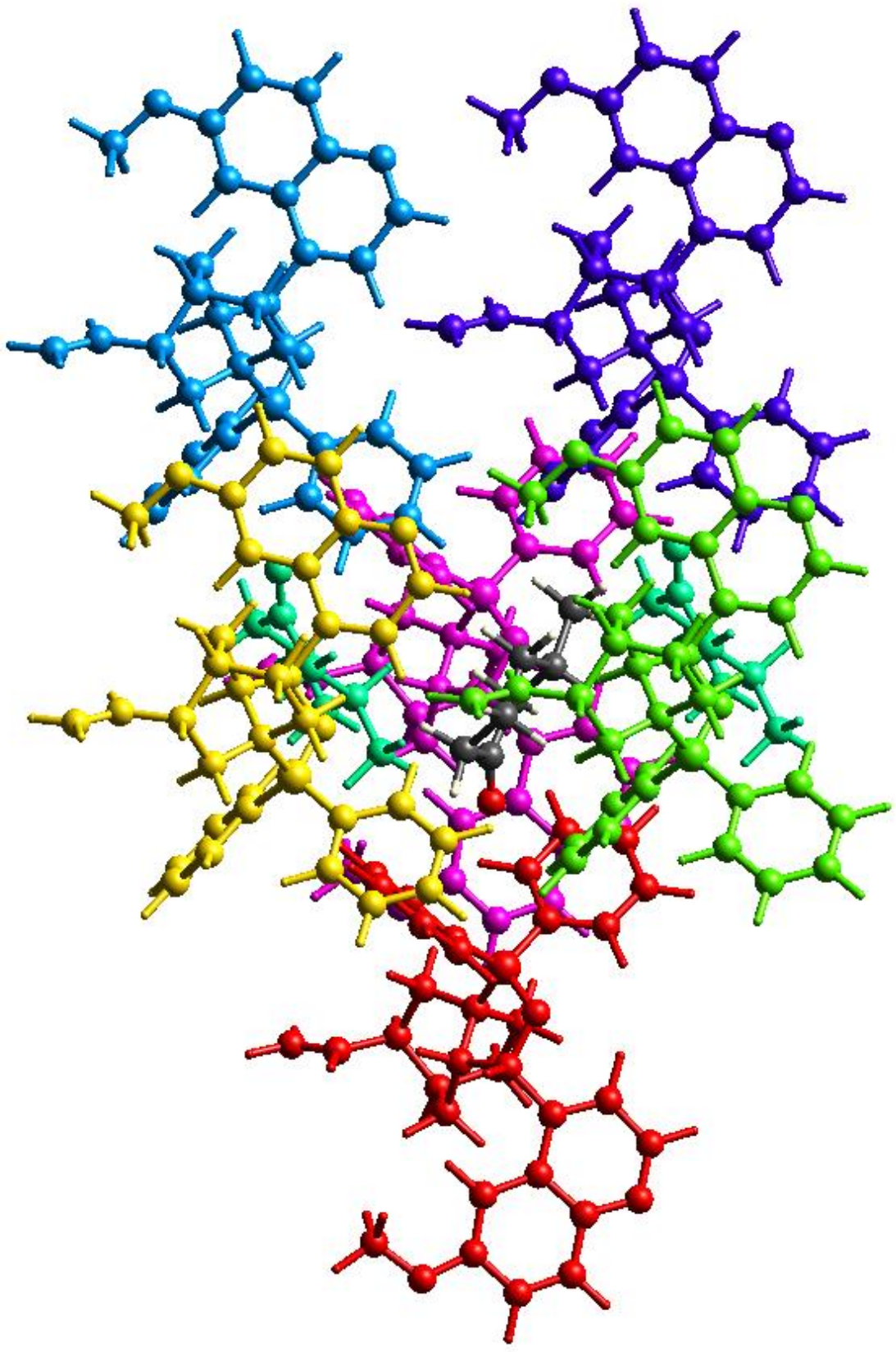

Table S23. Calculated host–guest interaction energies for the model B^S (**4**•(3-MCH)) with individual neighboring molecules, as well as the total interaction energy, decomposed into electrostatic (E_{ele}), polarization (E_{pol}), dispersion (E_{dis}), and exchange-repulsion (E_{rep}) components. Energies are given in kJ mol^{-1}.

	R [Å]	HS area [Å^2]	E_{ele}	E_{pol}	E_{dis}	E_{rep}	E_{tot}
(green)	5,09	59.9	-34,20	-10,20	-90,30	135,40	-38,70
(yellow)	6,58	27.7	-8,60	-1,40	-36,40	33,60	-21,20
(violet)	10,22	15.2	0,30	-0,20	-7,60	1,20	-5,70
(aquamarine)	6,82	14.4	-1,00	-0,50	-5,30	1,10	-5,30
(aquamarine)	6,82	15.4	-1,00	-0,50	-5,30	1,10	-5,30
(blue)	11,04	12.9	-0,60	-0,20	-6,60	2,90	-4,80
(magenta)	8,38	5.9	-0,40	-0,30	-3,10	0,00	-3,40
(red)	8,35	21.0	9,70	-7,20	-23,20	23,80	-0,50
		Total:	-35,80	-20,50	-177,80	199,10	-84,90

References

(1) CrysAlisPro, Data Collection and Processing Software for Agilent X-Ray Diffractometers, Ver. 1.171.35.21b, Agilent Technologies, 2012.

(2) Sheldrick, G. M. Phase Annealing in SHELX-90: Direct Methods for Larger Structures. *Acta Crystallogr. A* **1990**, *46* (6), 467–473. https://doi.org/10.1107/S0108767390000277.

(3) Sheldrick, G. M. SHELX-97 (Program for the Refinement of Crystal Structures), University of Göttingen, Göttingen (Germany), 1997.

(4) Dolomanov, O. V.; Bourhis, L. J.; Gildea, R. J.; Howard, J. A. K.; Puschmann, H. *OLEX2* : A Complete Structure Solution, Refinement and Analysis Program. *J. Appl. Crystallogr.* **2009**, *42* (2), 339–341. https://doi.org/10.1107/S0021889808042726.

(5) Farrugia, L. J. *WinGX* Suite for Small-Molecule Single-Crystal Crystallography. *J. Appl. Crystallogr.* **1999**, *32* (4), 837–838. https://doi.org/10.1107/S0021889899006020.

(6) Zhao, Y. H.; Abraham, M. H.; Zissimos, A. M. Fast Calculation of van Der Waals Volume as a Sum of Atomic and Bond Contributions and Its Application to Drug Compounds. *J. Org. Chem.* **2003**, *68* (19), 7368–7373. https://doi.org/10.1021/jo034808o.

(7) Kaczorowski, T.; Justyniak, I.; Lipińska, T.; Lipkowski, J.; Lewiński, J. Metal Complexes of Cinchonine as Chiral Building Blocks: A Strategy for the Construction of Nanotubular Architectures and Helical Coordination Polymers. *J. Am. Chem. Soc.* **2009**, *131* (15), 5393–5395. https://doi.org/10.1021/ja8098867.

(8) Jones, G.; Willett, P.; Glen, R. C.; Leach, A. R.; Taylor, R. Development and Validation of a Genetic Algorithm for Flexible Docking 1 1Edited by F. E. Cohen. *J. Mol. Biol.* **1997**, *267* (3), 727–748. https://doi.org/10.1006/jmbi.1996.0897.

(9) Spackman, P. R.; Turner, M. J.; McKinnon, J. J.; Wolff, S. K.; Grimwood, D. J.; Jayatilaka, D.; Spackman, M. A. *CrystalExplorer* : A Program for Hirshfeld Surface Analysis, Visualization and Quantitative Analysis of Molecular Crystals. *J. Appl. Crystallogr.* **2021**, *54* (3), 1006–1011. https://doi.org/10.1107/S1600576721002910.

(10) Mackenzie, C. F.; Spackman, P. R.; Jayatilaka, D.; Spackman, M. A. *CrystalExplorer* Model Energies and Energy Frameworks: Extension to Metal Coordination Compounds, Organic Salts, Solvates and Open-Shell Systems. *IUCrJ* **2017**, *4* (5), 575–587. https://doi.org/10.1107/S205225251700848X.

(11) Korb, O.; Wood, P. A. Prediction of Framework–Guest Systems Using Molecular Docking. *Chem. Commun.* **2010**, *46* (19), 3318. https://doi.org/10.1039/c003080e.
